# Sugar silanes: versatile reagents for stereocontrolled glycosylation *via* intramolecular aglycone delivery†
†Electronic supplementary information (ESI) available: Experimental details and copies of NMR spectra. See DOI: 10.1039/c5sc00810g


**DOI:** 10.1039/c5sc00810g

**Published:** 2015-04-14

**Authors:** Jordan T. Walk, Zachary A. Buchan, John Montgomery

**Affiliations:** a Department of Chemistry , University of Michigan , 930 N. University Ave. , Ann Arbor , MI 48109-1055 , USA . Email: jmontg@umich.edu

## Abstract

A new method for the intramolecular glycosylation of alcohols is described. Utilizing carbohydrate-derived silanes, the catalytic dehydrogenative silylation of alcohols is followed by intramolecular glycosylation. Appropriate combinations of silane position and protecting groups allow highly selective access to β-manno, α-gluco, or β-gluco stereochemical relationships as well as 2-azido-2-deoxy-β-gluco- and 2-deoxy-β-glucosides. Intramolecular aglycone delivery from the C-2 or C-6 position provides 1,2-*cis* or 1,2-*trans* glycosides, respectively. Multifunctional acceptor substrates such as hydroxyketones and diols are tolerated and are glycosylated in a site-selective manner.

## Abstract 2

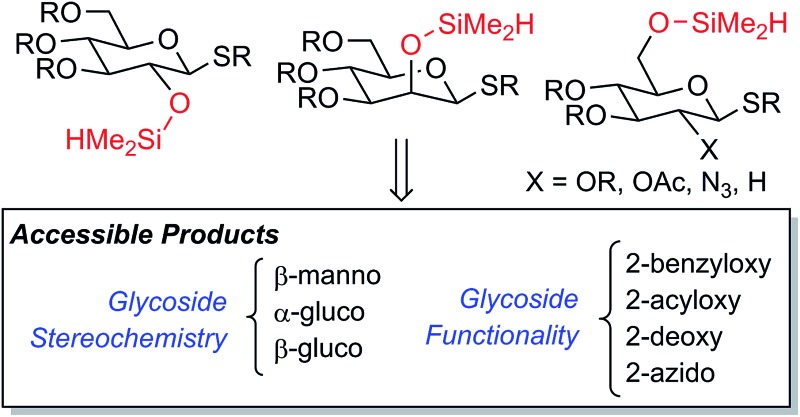
A new method for the intramolecular glycosylation of alcohols is described.

## Introduction

Despite the enormous progress that has been made in the development of chemical glycosylation methods, challenges remain in the efficiency and stereoselectivity of carbohydrate installation.^1^ Access to the various 1,2-stereochemical arrangements from a common carbohydrate donor is challenging, as careful matching of the anomeric leaving group, protecting groups that influence stereochemistry and reactivity, and reaction conditions is often required.^2^ The vast majority of methods require that only a single hydroxyl group is unmasked in the acceptor substrate. Furthermore, utilization of acceptor substrates other than alcohols and reactive electrophiles are virtually unexplored.^3^ Creative developments in intramolecular aglycone delivery,^4^ including the silicon-tethered version pioneered and developed by Stork^5^ and Bols,^6^ have served an important role in modern glycosylation technology. However, improved routes to the requisite tethered substrates and expansion of the range of accessible classes of glycoside products would significantly broaden the appeal and utility of these methods.

**Fig. 1:**
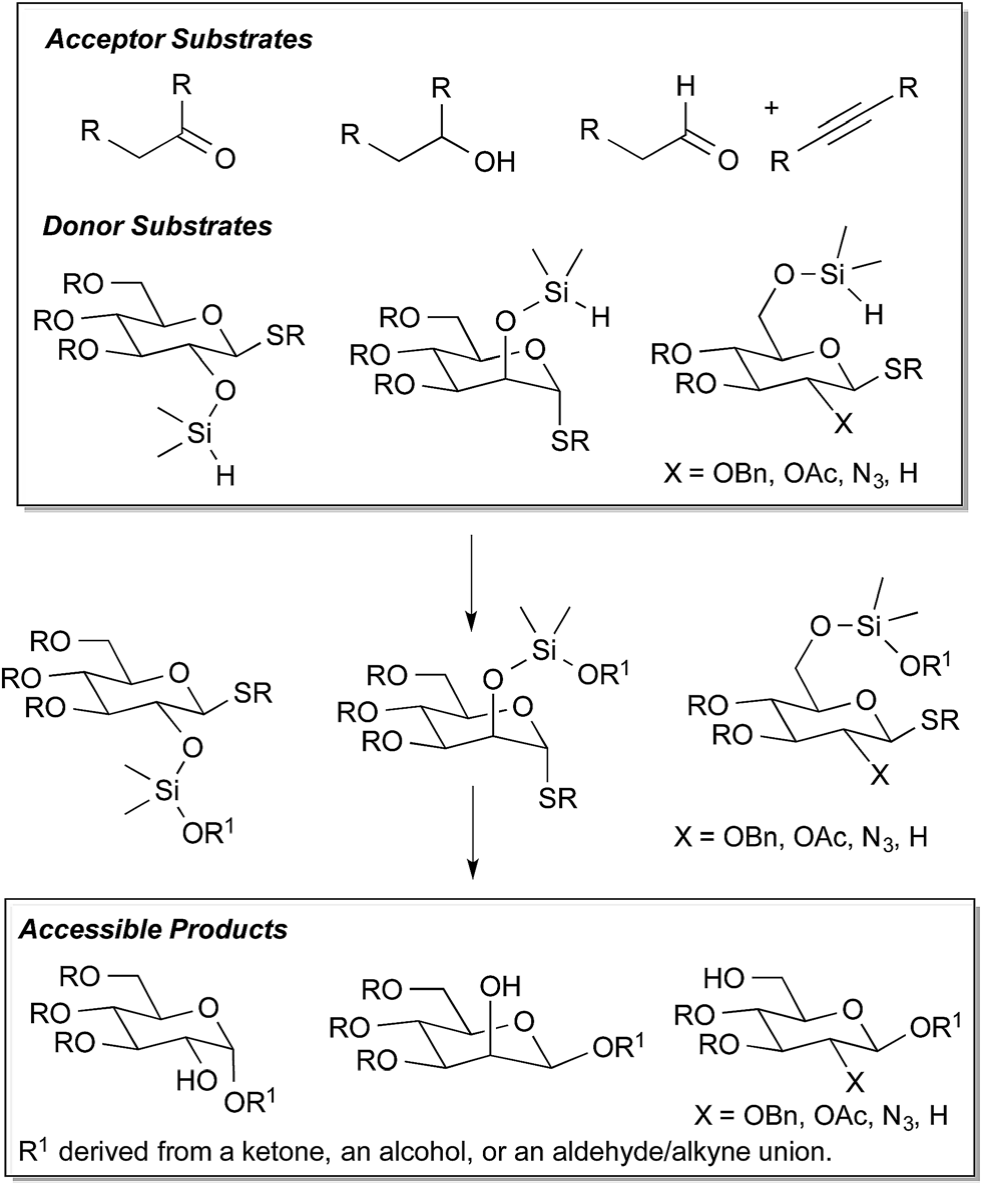
Donor–acceptor combinations for sugar silane-based glycosylations.

To address the above challenges and limitations, our laboratory has focused on the development of “sugar silanes” as a versatile reagent class that enables an array of glycosylation processes, providing access to numerous 1,2-stereochemical relationships and utilizing several different types of donor substrates (Fig. 1). Our prior efforts have described the direct reductive glycosylation of carbonyl substrates and the three-component assembly of glycosylated products *via* the catalytic union of aldehydes, alkynes, and sugar silanes.^7^ In order to provide a more complete toolbox of glycosylation procedures from sugar silanes, and to address the essential issue of site-selectivity among various reactive functional groups, we now report the direct glycosylation of alcohol substrates by the dehydrogenative condensation with sugar silanes. In addition to identifying effective catalysts to promote this new transformation, utilization of C-2^5^,^6^ or C-6^8^ intramolecular delivery of the glycosyl acceptor enables highly stereoselective access to β-manno, α-gluco, or β-gluco configurations. The work overcomes challenges that plagued previous strategies for intramolecular aglycone delivery from the C-6 position. Stereocontrolled access to β-glucosides with benzyloxy or acyloxy C-2 substituents as well as 2-azido and 2-deoxyglycosides is made possible by the new procedures described herein.

## Results and discussion

**Fig. 2:**
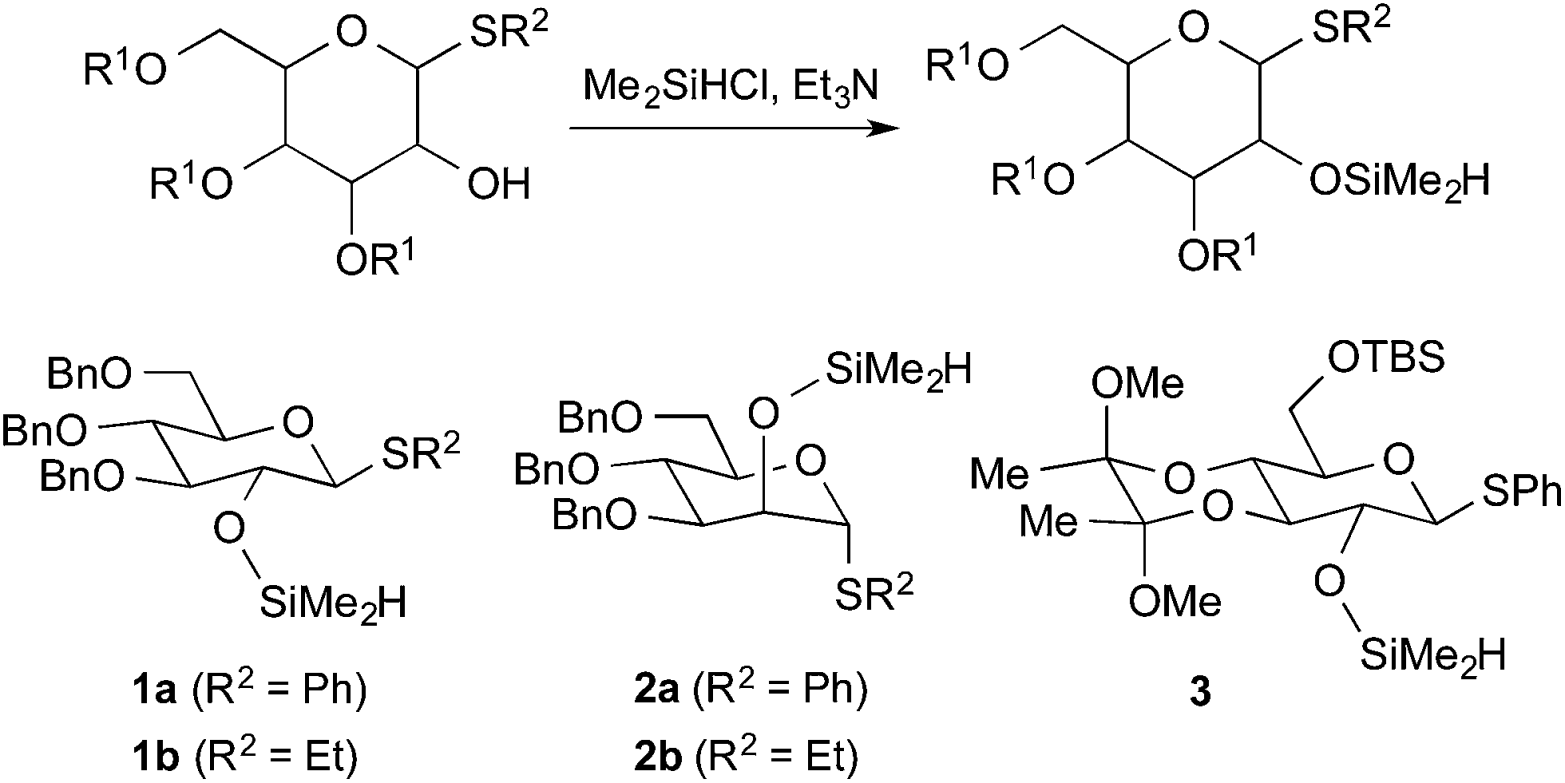
Synthesis of thioglycosides with a C-2 silane.

Given the precedent from Stork and Bols on intramolecular aglycone delivery from the C-2 position, our efforts first focused on the utility of mannose- and glucose-derived glycosyl donors bearing the requisite silane functionality at the C-2 position. These reagents are easily accessed in quantitative yield by C-2 protection with commercially available Me_2_SiHCl (Fig. 2). While bis-electrophilic reagents are most commonly used in the assembly of silyl linkages between two hydroxyls,^5^,^6^ the use of Me_2_SiHCl enjoys the advantage of high heterocoupling efficiency across a range of alcohol substrates.^9^ Following chloride displacement by the initially added alcohol, a second alcohol then condenses with the resulting silyl hydride (losing H_2_) in the presence of a transition metal or Lewis acid catalyst, thus effectively preventing homocoupling across a range of substrate combinations.

Upon screening numerous catalyst systems to promote the dehydrogenative coupling with alcohol acceptors, two catalyst systems were identified as most robust and exhibiting complementary behavior. While the methods were often interchangeable with similar results, the use of B(C_6_F_5_)_3_ was most effective with more hindered 2° and 3° alcohol substrates,^10^ whereas a copper–IMes catalyst was most effective with 1° alcohols.^11^ As the following examples illustrate, a range of hindered and unhindered glycosidic linkages may be created by this method (Table 1). Couplings of menthol with glucose-derived silane **1a** are effective using either CuCl–IMes or B(C_6_F_5_)_3_ as catalyst, with the latter allowing dehydrogenative coupling to afford silane intermediate **4a** in near quantitative yield. The silyl-linked intermediates were stable to silica gel chromatography and were purified prior to glycosylation. Intramolecular glycosylation with *N*-iodosuccinimide, trimethylsilyl triflate, and 2,6-di-(*t*-butyl)-4-methylpyridine cleanly afforded α-glucoside **5a** as a single stereoisomer in 98% isolated yield.3*b* Alternatively, mannose-derived silane **2a** allowed the production of β-mannoside **5b** in excellent overall yield as a single stereoisomer using either CuCl–IMes or B(C_6_F_5_)_3_ in the silylation step. Tolerance of acetal and silyl protecting groups was demonstrated through the formation of α-glucoside **5c** as a single stereoisomer. The method may be applied to the synthesis of disaccharides as demonstrated by the formation of α-glucoside **5d** as a single stereoisomer. Additionally, the iterative potential of the method is demonstrated by the synthesis of β-mannoside **5e**. In this example, the product **5b** is directly converted with sugar silane **1b** to intermediate **4e**. Intramolecular glycosylation then directly affords product **5e** as a single stereoisomer. Given that the C-2 hydroxyl is deprotected during glycosylation, this method may be especially attractive for the synthesis of oligosaccharides that possess repeating C-2 glycosidic linkages. While this method allows glycosylations of the C-2 hydroxyl of mannose as example **5e** illustrates, glycosylations of more hindered secondary hydroxyl sugar acceptors were not effective. While the above method builds upon the seminal contributions from Stork and Bols, the streamlined catalytic access to the silicon-tethered intermediates using readily accessible sugar silane reagents advances the practicality of the approach.

**Table 1:** β-Mannosides and α-glucosides by C-2 delivery

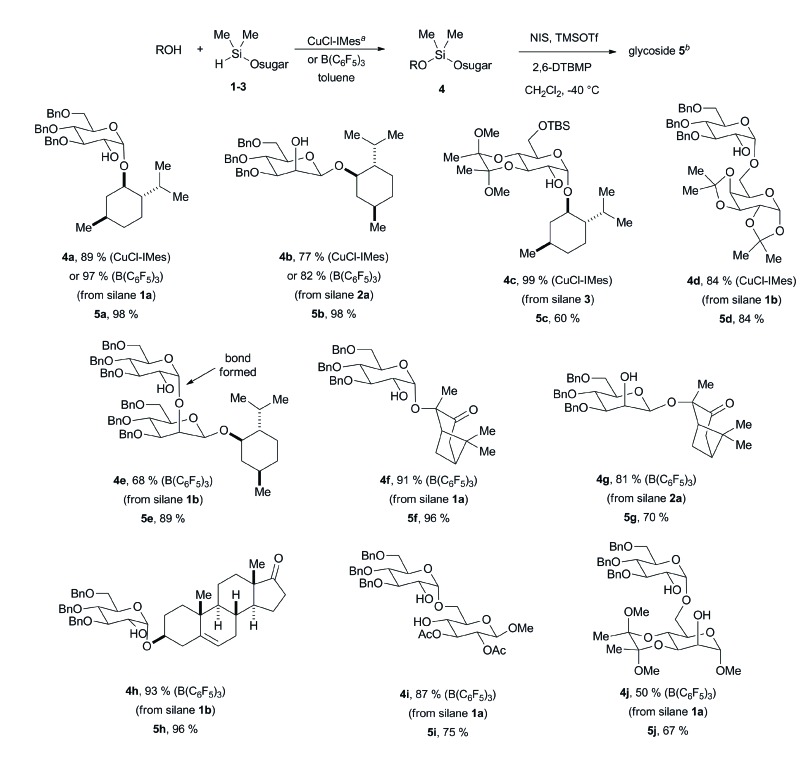

^*a*^IMes = 1,3-bis(mesityl)imidazol-2-ylidene.

^*b*^Diastereoselectivities of glycosylations are >97 : 3 as judged by ^1^H NMR.

Given the range of acceptor substrates that are amenable to glycosylation with sugar silanes (Fig. 1), the chemoselective or regioselective derivatization of a single functional group in a multifunctional substrate becomes a significant question to address.^12^ As entropic factors govern the intramolecular glycosylation, the chemoselectivity of the initial attachment of the sugar silane to a single functional group introduces a strategy for site-selective glycosylation of complex multifunctional substrates. Since both ketones and alcohols are competent substrates for the catalytic addition of sugar silanes, chemo- and regioselective additions to both hydroxyketones and to diols would provide important advances towards site-selective glycosylation. To address this question, the dehydrogenative silylation of 2-hydroxypinanone was examined. For this substrate, highly chemoselective dehydrogenative silylation of the hydroxyl group was observed with both the glucose and mannose-derived sugar silane to enable the production of α-glucoside **5f** or β-mannoside **5g** as single stereoisomers through the intermediacy of **4f** and **4g**. To examine a spatial separation of the ketone and hydroxyl functionalities, the dehydrogenative silylation of a steroid framework was examined, with both the silylation and glycosylation proceeding in excellent yield. In this case, an A-ring hydroxyl was silylated without affecting the D-ring ketone, enabling the production of β-mannoside **5h** as a single stereoisomer through the intermediacy of **4h**. Finally, two examples involving the C-6 selective glycosylation of a sugar diol were illustrated. The reaction of a 4,6-diol derived from glucose underwent selective C-6 silylation and α-glucosylation to afford **5i**. Similarly, reaction of a 2,6-diol derived from mannose underwent selective C-6 silylation and α-glucosylation to afford **5j**.

**Fig. 3:**
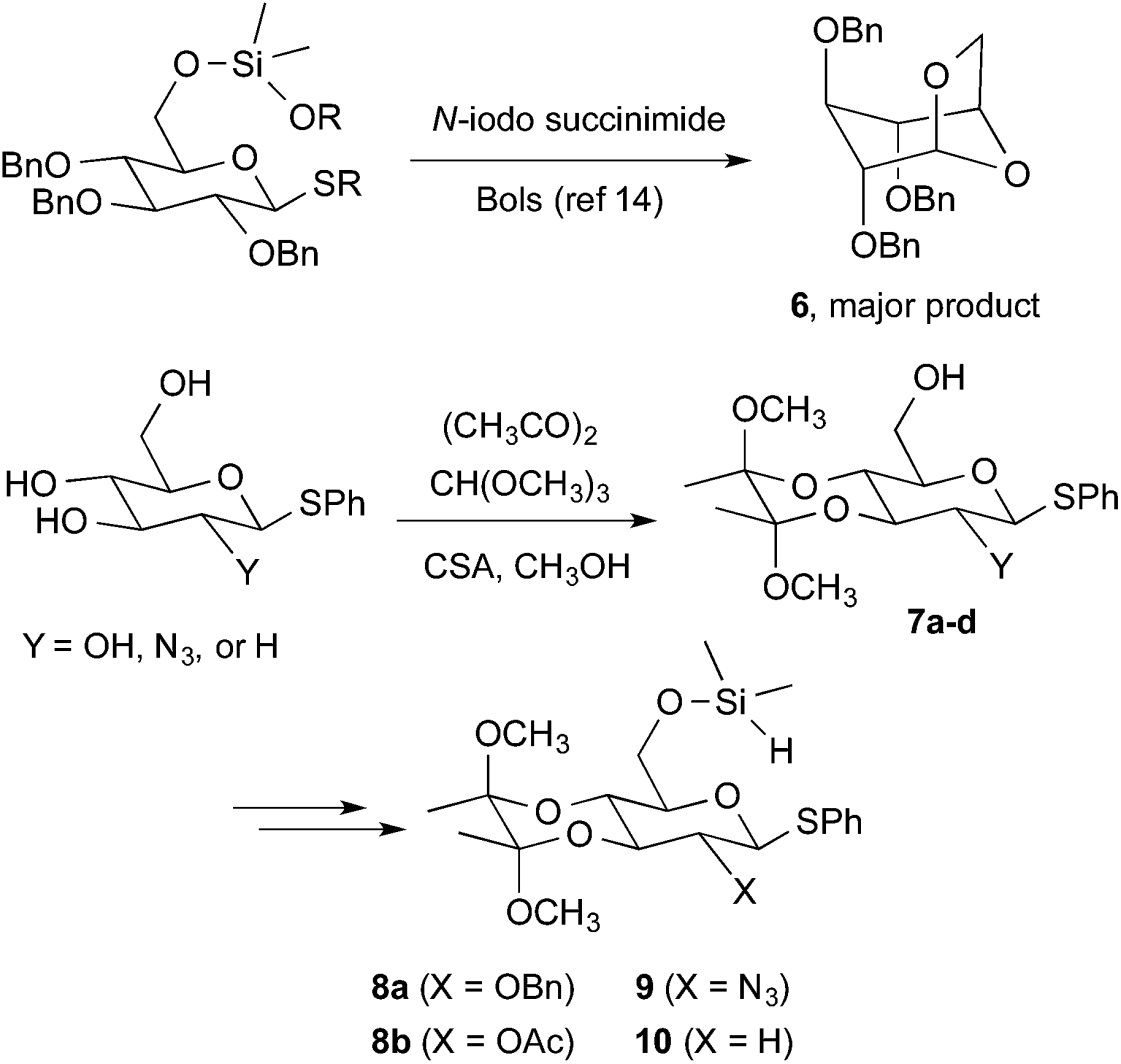
Synthesis of thioglycosides with a C-6 silane utilizing the Ley bis-acetal protection.

Whereas the synthesis of β-manno and α-gluco stereochemical relationships has been achieved through alternative intramolecular methods, an efficient strategy for β-gluco stereochemical relationships by intramolecular aglycone delivery has not been previously developed.^13^ Early efforts from Bols demonstrated that C-6 delivery is unsuccessful since bridged bicyclic product **6** is the major product *via* intramolecular delivery of the internal C-6 oxygen rather than the desired tethered aglycone (Fig. 3).^14^ While successes were seen with ribose frameworks,^14^ long-range intramolecular delivery with pyranosides remains an unsolved problem. We reasoned that installation of a conformational bias that prevents chair–chair interconversion should inhibit delivery of the undesired C-6 oxygenation. To evaluate this hypothesis, we utilized the strategy pioneered by Ley to protect the C-3/C-4 *trans*-diol *via* the cyclic bisacetal **7** (Fig. 3).^15^ This protecting group serves the two useful purposes of (1) providing streamlined access to the desired protecting group array, wherein late-stage installation of the C-6 silane is easily allowed, and (2) removing the conformational flexibility that allows the undesired [3.2.1]-oxabicyclic product **6** to form. This strategy allows straightforward access to a range of thiophenyl sugar silanes **8–10** bearing a C-6 silane, a C-3/C-4 *trans*-diol protected as the bis-acetal, and a range of C-2 substituents including benzyloxy, acetoxy, azido, and hydrogen substituents.

Silylations of alcohols using sugar silanes **8–10** were typically conducted with a CuCl–IPr catalyst derived from a 1 : 2 Cu : IPr ratio (Table 2).^16^ Reactions of these silanes were most efficiently accomplished with this hindered catalyst, compared with the CuCl–IMes catalyst that was utilized with silylations of the more hindered C-2 sugar silanes **1–3** (Table 1). As a first pair of examples of the C-6 delivery strategy, the intramolecular glycosylations of butanol and phenethyl alcohol were conducted using sugar silane **8a**. In both cases, β-glucoside products **12a** and **12b** were obtained as single stereoisomers (Table 2). Simple secondary alcohols such as cyclohexanol were effective participants as evidenced by the production of **12c** in good yield as a single stereoisomer. Unlike the C-2 delivery procedures that tolerate hindered alcohols, secondary alcohols more hindered than cyclohexanol were poor substrates for C-6 delivery. More hindered substrates such as menthol and C-2 hydroxyl acceptors derived from glucose underwent glycosylation in moderate to low yield. Given this limitation, the CuCl–IPr catalyst system was used in the majority of the C-6 delivery examples as this method is most effective with the primary acceptor alcohols employed.

**Table 2:** β-Glucosides by C-6 delivery

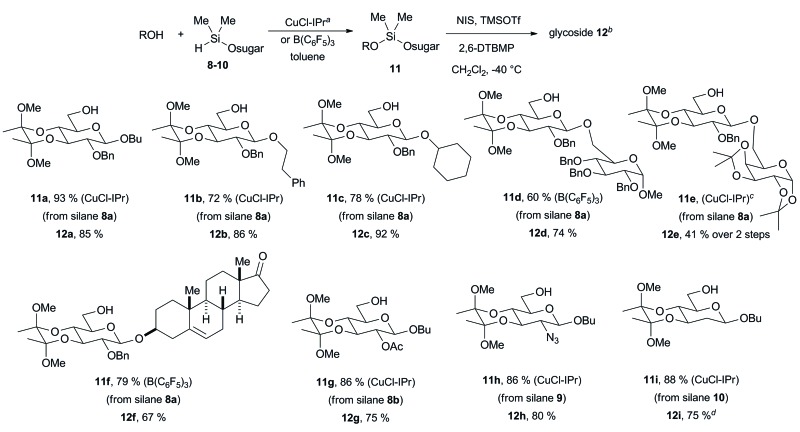

^*a*^IPr = 1,3-bis(2,6-diisopropylphenyl)imidazol-2-ylidene.

^*b*^Diastereoselectivities of glycosylations are >97 : 3 as judged by ^1^H NMR.

^*c*^Product **11e** was contaminated with a homodimer of sugar silane **8a** that was easily removed after conversion to **12e**.

^*d*^Glycosylation was conducted at –78 °C.

The formation of C-6 linked disaccharides proceeded efficiently, as evidenced by the formation of products **12d** and **12e**. In the former case (**12d**), coupling using B(C_6_F_5_)_3_ avoided the formation of a homocoupling product derived from partial hydrolysis of the sugar silane reagent. In the latter case (**12e**), a small amount of an impurity derived from dimerization of the sugar donor **8a** was inseparable from intermediate **11e**. Nonetheless, subjecting the mixture to glycosylation conditions led to the clean production of product **12e**, which was easily purified. Additionally, an example demonstrated the chemoselectivity for hydroxyls over ketones in the production of **12f**. In this case, the site-selective alcohol silylation is best accomplished with B(C_6_F_5_)_3_ as the catalyst.

In addition to C-2 benzyloxy examples **12a–f**, sugar silanes possessing C-2 acetoxy substituents, C-2 azido substituents, and those lacking C-2 substitution were cleanly tolerated in the production of **12g–i**. It should be noted that the directing influence of C-2 acetyl and C-2 benzoyl protecting groups is commonly employed in the facile synthesis of β-glucosides. However, β-selective glycosylation of donors lacking C-2 acyloxy substituents, such as benzyloxy, 2-azido-2-deoxy,^17^ and 2-deoxyglycosides,2a,^18^ present much more challenging substrates for controlled β-selective glycosylation. Interestingly, recent studies have shown that 3,4-*trans*-cyclic protecting groups with 2-deoxyglycosides favor α-selective intermolecular glycosylations, which are thus fully complementary to the β-selective intramolecular process illustrated herein.18*b* With the exception of one example (**12g**), each of the examples in Table 2 involves the more challenging classes of substrates that lack a stereochemistry-directing C-2 substituent.

**Fig. 4:**
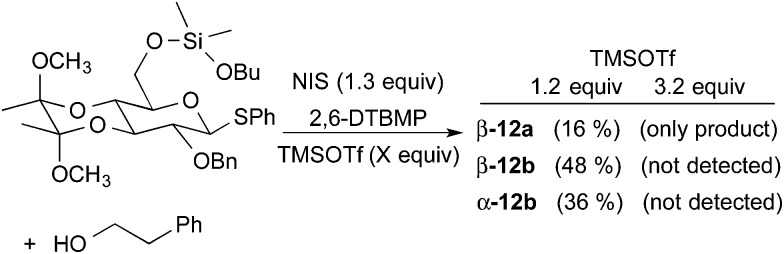
Evidence for intramolecularity of glycosylation.

Given the difficulties noted above in prior strategies for C-6 delivery with alternate protecting groups (Fig. 3), control experiments were conducted to ensure that glycoside formation proceeds by an intramolecular process. Repeating the conversion of silyl intermediate **11a** to glycoside **12a** in the presence of exogenous phenethyl alcohol resulted in the formation of **12a** (16%), **12b** (48%), and the α-anomer of **12b** (36%) as judged by analysis of the crude reaction mixture. However, increasing the amount of TMSOTf (3.2 equiv.) to enable complete silylation of the exogenous alcohol produced only **12a** (Fig. 4). These results illustrate that silyl cleavage followed by intermolecular glycosylation is not occurring under the standard method reported (Table 2) since only the intramolecular delivery is β-selective. Furthermore, addition of excess TMSOTf effectively suppresses the glycosylation of free alcohols and enables the β-selective intramolecular delivery to exclusively proceed when an exogenous alcohol is present.

## Conclusions

In summary, this work demonstrates a versatile new method for stereoselective glycosylation, utilizing the catalytic dehydrogenative coupling of sugar silane reagents with simple alcohols followed by silicon-tethered intramolecular aglycone delivery. The current work builds upon the known intramolecular glycosylation by C-2 delivery, and a new strategy enabling the stereochemically complementary and previously inaccessible delivery from C-6 has been developed by utilizing conformational constraints placed in the sugar donor. Put together, these methods provide great flexibility in the construction of glycosidic bonds with the available linkages including those of the β-manno, α-gluco, or β-gluco type. Additionally, challenging substrate classes including 2-benzyloxy, 2-azido, and 2-deoxy sugars are tolerated by the method, with the current procedure allowing glycosylation of primary hydroxyls. The site-selective glycosylation of hydroxyketones and sugar diols is enabled through this approach with proper selection of the dehydrogenative silylation catalyst. Future work will focus on the utilization of this work in combination with previously developed methods^7^ in increasingly complex illustrations of site-selective glycosylation of polyfunctional substrates.

## Supplementary Material

Supplementary information
