# Supplementary material for: Sugar silanes: versatile reagents for stereocontrolled glycosylation via intramolecular aglycone delivery
Source: Chem Sci. 2015 Apr 14;6(6):3448–53. doi: 10.1039/c5sc00810g (PMC4435934; doi:10.1039/c5sc00810g)

Electronic Supplementary Material (ESI) for Chemical Science.
This journal is © The Royal Society of Chemistry 2015

Supplementary Information

Sugar Silanes: Versatile Reagents for Stereocontrolled Glycosylation via Intramolecular Aglycone Delivery

Jordan T. Walk, Zachary A. Buchan, and John Montgomery*

Department of Chemistry, University of Michigan, 930 N. University Avenue, Ann Arbor, MI 48109-1055, United States, jmontg@umich.edu

All reagents were used as received unless otherwise noted. Solvents were purified under nitrogen using a solvent purification system (Innovative Technology, Inc., Model # SPS-400-3 and PS-400-3). Copper (I) chloride (CuCl, Strem, used as received), 1,3-bis-(2,6-diisopropylphenyl)-imidazolium chloride (IPr•HCl, Aldrich, used as received), chloro[1,3-bis(2,6-diisopropylphenyl)imidazol-2-ylidene]copper (CuCl•IPr, Aldrich, used a received), 1,3-dimesitylimidazolium chloride (IMes•HCl, Strem, used as received), potassium *tert*-butoxide (KO*t*Bu, Strem, used as received), and sodium *tert*-butoxide (NaO*t*Bu, Aldrich, used as received) were stored and weighed in an inert atmosphere glovebox. Tris(pentafluorophenyl)borane ($B(C_6F_5)_3$, Aldrich, used as received) was stored and weighed in an inert atmosphere glovebox but was also found to perform equally well when stored in a vial kept in a dissicator and weighed on the benchtop. Chlorodimethylsilane (Me_2SiHCl, Aldrich) was distilled under N_2 and transferred to a Schlenk flask at 0 °C for storage. Triethylamine (NEt_3, Aldrich) was freshly distilled under N_2 prior to use. Trimethylsilyltrifluoromethylsulfonate (TMSOTf, Aldrich) was distilled under vacuum and transferred to a Schlenk tube for storage. All $B(C_6F_5)_3$ and Cu/NHC reactions were conducted in flame-dried glassware under a nitrogen atmosphere. Powdered 4 Å molecular sieves were dried overnight before use at 150 °C at less than 1 torr and stored in an oven at 130 °C. ^1H and ^{13}C spectra were obtained in $CDCl_3$ at rt, unless otherwise noted, on a Varian Mercury 400, Varian Unity 500, Varian vnmrs 500, or Varian vnmrs 700 MHz instrument. Chemical shifts of ^1H NMR spectra were recorded in ppm from the central peak of $CDCl_3$ (7.25 ppm) or CD_3OD (3.31 ppm) on the δ scale. Chemical shifts of ^{13}C NMR spectra were recorded in ppm from the central peak of $CDCl_3$ (77.0 ppm) or CD_3OD (49.0 ppm) on the δ scale. NMR spectra are described using first order analysis. High resolution mass spectra (HRMS) were obtained on a VG-70-250-S spectrometer manufactured by Micromass Corp. (Manchester UK) at the University of Michigan Mass Spectrometry Laboratory.

General Procedure A – Preparation of Sugar Silanes[1]

The respective 2-OH or 6-OH sugar (1.0 equiv) was dissolved in dry CH_2Cl_2 (0.2 M) and cooled to 0 °C in an ice bath. Freshly distilled NEt_3 (2.0 equiv) was added and stirred for 3 min, then Me_2SiHCl (1.5 equiv) was added. The reaction was allowed to stir for 4 h. Volatiles were removed by rotary evaporation. The resulting oil was extracted from $NaHCO_3$ (aq.) (diluted over ice) 3 times with CH_2Cl_2. The combined organic extracts were dried quickly over $MgSO_4$, filtered, concentrated, and the resulting solid or oil was stored under vacuum or frozen in C_6H_6.

Note – the sugar silanes are stable for months when stored frozen in benzene or under high vacuum. Alternatively, the corresponding 2-OH or 6-OH sugars are very stable to be stored for long periods of time on the bench top.

General Procedure B – $B(C_6F_5)_3$ Promoted Coupling of Alcohols and Sugar Silanes

A mixture of sugar silane (1.0-1.5 equiv) and alcohol (1.0 equiv) was dissolved in dry toluene (0.1 M) at rt under an inert atmosphere (N_2) and stirred until both substrates were completely dissolved. $B(C_6F_5)_3$ (2-4 mol%) was added as a solid under a gentle stream of nitrogen followed by re-attachment of the septum and nitrogen line. Alternatively, a mixture of alcohol (1.0 equiv) and $B(C_6F_5)_3$ (5 mol%) were dissolved in dry toluene (0.1 M) at rt under an inert atmosphere (N_2) with 4 Å MS (400 mg/mmol). Sugar silane (1.5 equiv) was dissolved in toluene (0.5 – 0.75 M) and added slowly over 1 h. Upon completion of the reaction as monitored by TLC, the reaction mixture was either loaded directly onto a column or the volatiles were removed by rotary evaporation and then loaded onto a column for purification by flash chromatography (SiO_2) to afford the desired product. Note – dry CH_2Cl_2 was found to be the optimal co-solvent if a substrate is marginally soluble in toluene.

General Procedure C – CuCl/NHC•HCl and CuCl•NHC Promoted Dehydrogenative Silylation of Alcohols with Sugar Silanes

A solid mixture of CuCl (5 mol%), NHC•HCl (5 – 10 mol%), and KO*t*Bu or NaO*t*Bu (5 – 10 mol%) was dissolved in dry $PhCH_3$ (0.015 M) at rt under an inert atmosphere (N_2) and stirred for 15 min. A mixture of alcohol (1.0 equiv), silane (1.1 equiv), and 4 Å MS (0 – 400 mg/mmol) was dissolved in dry $PhCH_3$ (0.1 – 0.2 M) and the catalyst was added to this mixture as a solution in dry $PhCH_3$. Upon completion of the reaction as monitored by TLC, the reaction mixture was filtered through a short plug of silica gel with 50% EtOAc/hex and concentrated by rotary evaporation. The resulting residue was purified via flash chromatography (SiO_2) to afford the desired product.

Alternatively, a solid mixture of CuCl•NHC (5 mol%) and KO*t*Bu or NaO*t*Bu (5 mol%) can be used in the above procedure.

General Procedure D – NIS/TMSOTf Promoted Glycosylation of Silyl-linked Compounds

The respective silyl-linked compound (1.0 equiv) was dissolved in dry CH_2Cl_2 (0.02 M) and cooled to -78 or -40 °C. *N*-iodosuccinimide (1.3 – 1.4 equiv) and 2,6-DTBMP (2.0 – 4.0 equiv) were added and stirred for 3 – 5 min. To this solution was added TMSOTf (1.2 – 2.4 equiv) and the reaction was stirred for 5 – 20 min followed by warming to 0 °C unless otherwise noted. Upon disappearance of the silyl-linked compound as monitored by TLC, TBAF (5 equiv, 1 M in THF) was added and the reaction was warmed to rt and stirred overnight. The reaction mixture was quenched with $Na_2S_2O_3$ (sat. aq.) and extracted three times from NH_4Cl (sat. aq.) with CH_2Cl_2. The combined organic extracts were dried over $MgSO_4$, filtered, and concentrated by rotary evaporation. The resulting residue was purified by flash chromatography (SiO_2) to afford the desired product. Note – All experimentals include a bolded diagnostic ^{1}H NMR peak for the assignment of anomeric stereochemistry.

Phenyl 6-*O*-*tert*-butyldimethylsilyl-3,4-*O*-(2',3'-dimethoxybutane-2',3'-diyl)-thio-β-D-glucopyranoside (13)

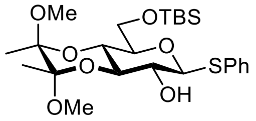

To a solution of 3,4-*O*-(2',3'-dimethoxybutane-2',3'-diyl)-thio-β-D-glycoside[1] (6.3 g, 16 mmol) in pyridine (82 mL, 0.2 M) was added TBSCl (2.7 g, 18 mmol). The solution was stirred for 4 h and then quenched with MeOH, diluted with Et_2O, washed with H_2O (x3), dried over $MgSO_4$, filtered, and concentrated to give the product (6.4 g, 13 mmol, 78%) as a white foam. ^{1}H NMR (700 MHz, $CDCl_3$) δ 7.58 (dd, J = 7.7, 1.4 Hz, 2H), 7.25-7.32 (m, 3H), 4.53 (d, J = 9.1 Hz), 3.89 (dd, J = 11.2, 1.4 Hz), 3.84 (dd, J = 11.2, 3.5 Hz, 1H), 3.69-3.77 (m, 2H), 3.50 (t, J = 9.1 Hz), 3.31 (s, 3H), 3.26 (s, 3H), 2.40 (s, 3H), 1.34 (s, 3H), 1.30 (s, 3H), 0.91 (s, 9H), 0.09 (s, 3H), 0.08 (s, 3H); ^{13}C NMR (175 MHz, $CDCl_3$) δ 133.2, 131.6, 128.9, 128.1, 99.7, 99.4, 88.4, 78.9, 73.7, 69.2, 64.7, 61.2, 48.1, 47.9, 25.9, 18.4, 17.7, 16.6, -5.1, -5.4; IR (film, cm^{-1}) 3350, 2947, 2040, 1472, 1252, 1117, 1074, 1022; HRMS (ES) *m/z* calcd for $C_{24}H_{40}O_7SSi$ $[M+Na]^+$ 523.2156, found 523.2155.

Sugar Silane (3)

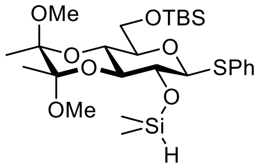

Following general procedure A, **13** (0.58 g, 1.2 mmol), NEt_3 (323 μL, 2.32 mmol), and Me_2SiHCl (193 μL, 1.74 mmol) were stirred for 4 h at 0 °C. The product (0.62 g, 1.1 mmol, 96%) was obtained as a red oil after aqueous work-up. ^{1}H NMR (500 MHz, $CDCl_3$) δ 7.54-7.57 (m, 2H), 7.22-7.29 (m, 3H), 4.70 (sept, J = 2.0 Hz, 1H), 4.56 (d, J = 9.5 Hz, 1H), 3.86 (dd, J = 11.5, 2.0 Hz, 1H), 3.82 (dd, J = 11.5, 4.0 Hz, 1H), 3.72 (t, J = 9.5 Hz, 1H), 3.67 (t, J = 9.5 Hz, 1H), 3.59 (t, J = 9.0 Hz, 1H), 3.45 (ddd, J = 9.0, 3.5, 1.5 Hz, 1H), 3.28 (s, 3H), 3.27 (s, 3H), 1.31 (s, 3H), 1.30 (s, 3H), 0.91 (s, 9H), 0.27 (d, J = 2.5 Hz, 3H), 0.24 (d, J = 2.5 Hz, 3H), 0.09 (s, 3H), 0.06 (s, 3H); ^{13}C NMR (100 MHz, $CDCl_3$) δ 133.9, 131.9, 128.7, 127.2, 99.7, 99.4, 89.1, 78.6, 74.5, 72.4, 64.9, 61.4, 48.0, 47.8, 25.9, 18.4, 17.6, 17.6, -0.4, -0.8, -5.1, -5.4; IR (film, cm^{-1}) 2992, 2954, 2930, 2857, 2131, 1472, 1375, 1252, 1139, 1113, 1075, 1042; HRMS (ES) *m/z* calcd for $C_{26}H_{46}O_7SSi_2$ $[M+NH_4]^+$ 576.2841, found 576.2839.

Sugar Silane 1a and (-)-Menthol Silyl-Linked (4a)

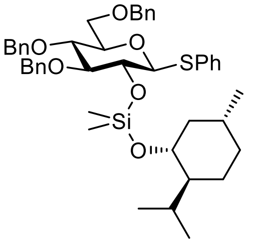

Following general procedure B, **1a** (60 mg, 0.10 mmol), (-)-menthol (16 mg, 0.10 mmol), and $B(C_6F_5)_3$ (2 mg, 0.004 mmol) were stirred for 85 min at rt. The product (73 mg, 0.097 mmol, 97%) was obtained as a colorless oil upon purification by flash chromatography (5 to 8% EtOAc/hex on SiO_2. ^{1}H NMR (500 MHz, $CDCl_3$) δ 7.53-7.56 (m, 2H), 7.38-7.41 (m, 2H), 7.21-7.35 (m, 14H), 7.14-7.17 (m, 2H), 5.02 (d, *J* = 11.0 Hz, 1H), 4.86 (d, *J* = 11.0 Hz, 1H), 4.78 (d, *J* = 10.5 Hz, 1H), 4.65 (d, *J* = 9.5 Hz, 1H), 4.60 (d, *J* = 12.0 Hz, 1H), 4.57 (d, *J* = 10.5 Hz, 1H), 4.54 (d, *J* = 12.0 Hz, 1H), 3.76-3.81 (m, 2H), 3.71 (dd, *J* = 11.0, 5.0 Hz, 1H), 3.53-3.66 (m, 4H), 2.21 (septd, *J* = 7.0, 2.5 Hz, 1H), 2.01 (d, *J* = 12.0 Hz, 1H), 1.53-1.63 (m, 2H), 1.28-1.37 (m, 1H), 1.12 (dt, *J* = 12.5, 3.0 Hz, 1H), 1.05 (q, *J* = 12.5 Hz, 1H), 0.86-0.97 (m, 1H), 0.86 (d, *J* = 7.0 Hz, 3H), 0.85 (d, *J* = 7.0 Hz, 3H), 0.72-0.82 (m, 1H), 0.74 (d, *J* = 7.0 Hz, 3H), 0.25 (s, 3H), 0.13 (s, 3H); ^{13}C NMR (100 MHz, $CDCl_3$) δ 138.7, 138.2, 138.0, 134.5, 131.1, 128.9, 128.4, 128.3, 128.2, 127.8, 127.73, 127.66, 127.52, 127.50, 127.3, 127.0, 88.8, 87.0, 79.0, 78.2, 75.2, 74.9, 73.9, 73.4, 72.7, 69.0, 49.7, 45.4, 34.5, 31.6, 25.2, 22.8, 22.2, 21.2, 15.9, -1.2,-1.4; IR (film, cm^{-1}) 3030, 2953, 2919, 2868, 1453, 1365, 1254, 1067; HRMS (ES) *m/z* calcd for $C_{45}H_{58}O_6SSi$ $[M+Na]^+$ 777.3621, found 777.3622.

Sugar Silane 1a and (-)-Menthol Glycoside (5a)

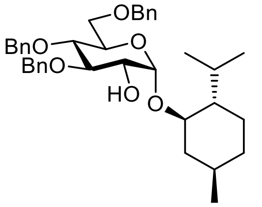

Following general procedure D, compound **4a** (148 mg, 0.197 mmol), NIS (57 mg, 0.25 mmol), TMSOTf (43 µL, 0.24 mmol), and 2,6-DTBMP (80 mg, 0.39 mmol) were stirred at -40 °C for 10 min, warmed to 0 °C and stirred for 30 min, and quenched with TBAF. The product (113 mg, 0.192 mmol, 98%) was obtained as a colorless oil upon purification by flash chromatography (10% EtOAc/hex) on SiO_2. ^{1}H NMR (500 MHz, $CDCl_3$) δ 7.39-7.42 (m, 2H), 7.25-7.37 (m, 11H), 7.14-7.17 (m, 2H), **4.99 (d, *J* = 4.5 Hz, 1H)**, 4.97 (d, *J* = 11.5 Hz, 1H), 4.85 (d, *J* = 11.5 Hz, 1H), 4.84 (d, *J* = 10.5 Hz, 1H), 4.66 (d, *J* = 12.0 Hz, 1H), 4.50 (d, *J* = 11.0 Hz, 1H), 4.48 (d, *J* = 10.0 Hz, 1H), 3.94 (ddd, *J* = 10.0, 3.5, 2.0 Hz, 1H), 3.77 (dd, *J* = 10.75, 4.25 Hz, 1H), 3.60-3.74 (m, 4H), 3.40 (td, *J* = 10.5, 4.0 Hz, 1H), 2.19 (d, *J* = 12.5 Hz, 1H), 2.14 (septd, *J* = 6.5, 2.5 Hz, 1H), 1.96 (d, *J* = 9.0 Hz, 1H), 1.61-1.68 (m, 2H), 1.34-1.45 (m, 1H), 1.25-1.33 (m, 1H), 0.94-1.06 (m, 2H), 0.92 (d, *J* = 7.0 Hz, 3H), 0.87 (d, *J* = 6.5 Hz, 3H), 0.78-0.86 (m, 1H), 0.78 (d, *J* = 7.0 Hz, 3H); ^{13}C NMR (100 MHz, $CDCl_3$) δ 138.8, 138.2, 138.0, 128.4, 128.3, 128.0, 127.92,

127.86, 127.7, 127.64, 127.60, 100.0, 83.5, 81.1, 77.4, 75.2, 75.0, 73.6, 73.5, 70.6, 68.6, 48.7, 42.9, 34.1, 31.6, 25.5, 22.8, 22.2, 21.2, 15.7; IR (film, cm^{-1}) 3566, 3062, 3030, 2922, 2868, 1454, 1362, 1133, 1067, 1028; HRMS (ES) *m/z* calcd for $C_{37}H_{48}O_6$ $[M+Na]^+$ 611.3349, found 611.3354.

Sugar Silane 2a and (-)-Menthol Silyl-Linked (4b)

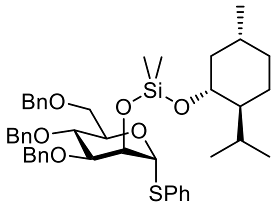

Following general procedure B, **2a** (60 mg, 0.10 mmol), (-)-menthol (16 mg, 0.10 mmol), and $B(C_6F_5)_3$ (2 mg, 0.004 mmol) were stirred at rt for 1.5 h. The product (62 mg, 0.082 mmol, 92%) was obtained as a colorless oil upon purification by flash chromatography (5 to 8% EtOAc/hex) on SiO_2. ^{1}H NMR (500 MHz, $CDCl_3$) δ 7.48-7.51 (m, 2H), 7.38-7.42 (m, 2H), 7.20-7.36 (m, 16H), 5.53 (s, 1H), 4.88 (d, *J* = 10.5 Hz, 1H), 4.78 (d, *J* = 11.5 Hz, 1H), 4.67 (d, *J* = 12.0 Hz, 2H), 4.55 (d, *J* = 11.0 Hz, 1H), 4.50 (d, *J* = 12.0 Hz, 1H), 4.47 (s, 1H), 4.30 (dd, *J* = 9.5, 5.0 Hz, 1H), 4.00 (t, *J* = 9.5 Hz, 1H), 3.85 (dd, *J* = 11.0, 5.0 Hz, 1H), 3.80 (dd, *J* = 9.0, 2.5 Hz, 1H), 3.76 (d, *J* = 11.0 Hz, 1H), 3.54 (td, *J* = 10.0, 4.5 Hz, 1H), 2.09-2.19 (m, 1H), 1.95 (d, *J* = 12.0 Hz, 1H), 1.52-1.63 (m, 2H), 1.25-1.35 (m, 1H), 1.08-1.16 (m, 1H), 1.01 (q, *J* = 12.0 Hz, 1H), 0.75-0.87 (m, 7H), 0.72 (d, *J* = 7.0 Hz, 3H), 0.17 (s, 3H), 0.15 (s, 3H); ^{13}C NMR (100 MHz, $CDCl_3$) δ 138.5, 138.4, 138.3, 134.7, 131.4, 128.9, 128.3, 128.2, 127.92, 127.86, 127.63, 127.57, 127.3, 127.1, 89.0, 80.3, 75.0, 74.9, 73.2, 73.0, 72.6, 72.2, 70.8, 69.3, 49.8, 45.3, 34.5, 31.6, 25.3, 22.8, 22.2, 21.1, 15.9, -1.35, -1.40; IR and HRMS match previously reported data for a mixture of diastereomers at the (-)-menthol carbinol.[1]

Sugar Silane 2a and (-)-Menthol Glycoside (5b)

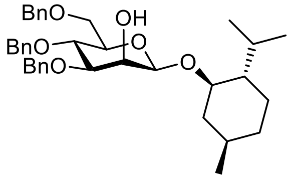

Following general procedure D, compound **4b** (130 mg, 0.17 mmol), NIS (50 mg, 0.2 mmol), TMSOTf (37 µL, 0.21 mmol), and 2,6-DTBMP (70 mg, 0.3 mmol) were stirred at -40 °C for 10 min, warmed to 0 °C and stirred for 90 min, and quenched with TBAF. The product (100 mg, 0.2 mmol, 98%) was obtained as a white solid upon purification by flash chromatography (10 to 20% EtOAc/hex) on SiO_2. All spectral data matches previously reported data.[2]

Sugar Silane 3 and (-)-Menthol Silyl-Linked (4c)

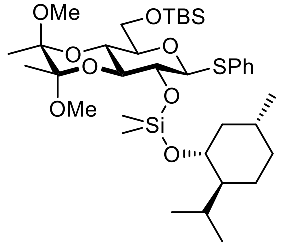

Following general procedure C, **3** (61 mg, 0.11 mmol), (-)-menthol (16 mg, 0.10 mmol), CuCl (0.5 mg, 0.005 mmol), IMes•HCl (1.7 mg, 0.0050 mmol), and NaO*t*Bu (1 mg, 0.01 mmol) were stirred at rt for 1 h. The product (72 mg, 0.010 mmol, 99%) was obtained as a colorless oil upon purification by flash chromatography (5 to 8% EtOAc/hex) on SiO_2. ^{1}H NMR (500 MHz, $CDCl_3$) δ 7.50-7.54 (m, 2H), 7.20-7.29 (m, 3H), 4.63 (d, *J* = 8.5 Hz, 1H), 3.87 (dd, *J* = 11.5, 1.5 Hz, 1H), 3.81 (dd, *J* = 11.5, 4.5 Hz, 1H), 3.66-3.75 (m, 3H), 3.62 (td, *J* = 10.5, 4.5 Hz, 1H), 3.46 (ddd, *J* = 9.0, 4.5, 2.0 Hz, 1H), 3.29 (s, 3H), 3.27 (s, 3H), 2.21 (septd, *J* = 7.5, 2.0 Hz, 1H), 2.03-2.09 (m, 1H), 1.54-1.64 (m, 2H), 1.24-1.36 (m, 1H), 1.31 (s, 3H), 1.30 (s, 3H), 1.15 (ddt, *J* = 12.0, 9.5, 2.5 Hz, 1H), 0.76-1.02 (m, 3H), 0.89 (s, 9H), 0.87 (d, *J* = 7.0 Hz, 3H), 0.83 (d, *J* = 6.5 Hz, 3H), 0.73 (d, *J* = 7.0 Hz, 3H), 0.21 (s, 3H), 0.20 (s, 3H), 0.08 (s, 3H), 0.03 (s, 3H); ^{13}C NMR (100 MHz, $CDCl_3$) δ 134.8, 130.9, 128.7, 126.8, 99.6, 99.3, 89.2, 78.5, 74.5, 72.5, 70.7, 65.1, 61.5, 49.9, 48.0, 48.0, 45.2, 34.5, 31.6, 25.9, 25.1, 22.9, 22.2, 21.3, 18.3, 17.6, 17.5, 16.0, -0.3, -1.8, -5.1, -5.5; IR (film, cm^{-1}) 2955, 2929, 2870, 1462, 1371, 1255, 1139, 1072, 1042; HRMS (ES) *m/z* calcd for $C_{26}H_{46}O_7SSi_2$ $[M+NH_4]^+$ 730.4199, found 730.4195.

Sugar Silane 3 and (-)-Menthol Glycoside (5c)

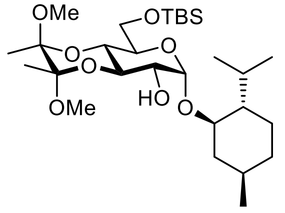

Following general procedure D, compound **4c** (67 mg, 0.094 mmol), NIS (27 mg, 0.12 mmol), TMSOTf (20 µL, 0.11 mmol), and 2,6-DTBMP (39 mg, 0.19 mmol) were stirred for 10 min at -40 °C, warmed to 0 °C and stirred for 65 min, and quenched with TBAF. The product (33 mg, 0.060 mmol, 60%) was obtained as a colorless oil upon purification by flash chromatography (10 to 15% EtOAc/hex) on SiO_2. ^{1}H NMR (500 MHz, $CDCl_3$) δ **4.96 (d, *J* = 4.0 Hz, 1H)**, 3.76-3.89 (m, 4H), 3.61-3.68 (m, 3H), 3.40 (dt, *J* = 10.5, 4.0 Hz, 1H), 3.32 (s, 3H), 3.27 (s, 3H), 2.08-2.21 (m, 2H), 1.91 (d, *J* = 10.5 Hz, 1H), 1.56-1.67 (m, 2H), 1.34-1.42 (m, 1H), 1.36 (s, 3H), 1.24-1.32 (m, 1H), 1.31 (s, 3H), 0.76-1.05 (m, 6H), 0.91 (d, *J* = 7.0 Hz, 3H), 0.90 (d, *J* = 6.5 Hz, 3H), 0.89 (s, 9H), 0.79 (d, *J* = 7.0, 3H), 0.06 (s, 3H), 0.05 (s, 3H); ^{13}C NMR (100 MHz, $CDCl_3$) δ 99.65, 99.60, 99.4, 80.6, 70.74, 70.68, 70.4, 65.7, 61.5, 48.8, 48.0, 47.9, 42.8, 34.2, 31.6, 25.9, 25.7, 23.0, 22.3, 21.1, 17.8, 17.7, 15.8, -5.1, -5.4; IR (film, cm^{-1}) 3490, 2954, 2930, 2871, 1638, 1458, 1376, 1252, 1138, 1035; HRMS (ES) *m/z* calcd for $C_{28}H_{40}O_8Si$ $[M+Na]^+$ 569.3480, found 569.3481.

Sugar Silane 1b and 1,2:3,4-*O*-Diisopropylidene-D-Galactopyranose Silyl-Linked (4d)

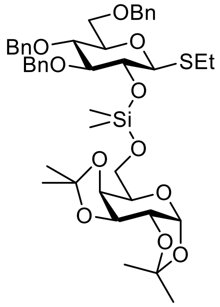

Following general procedure C, **1b** (61 mg, 0.11 mmol), 1,2:3,4-di-*O*-isopropylidene-galactopyranose (26 mg, 0.10 mmol), CuCl (0.5 mg, 0.005 mmol), IMes•HCl (1.7 mg, 0.0050 mmol), and NaO*t*Bu (1 mg, 0.01 mmol) were stirred at rt for 15 min. The product (68 mg, 0.084 mmol, 84%) was obtained as an oil upon purification by flash chromatography (10 to 15% EtOAc/hex) on SiO_2. ^{1}H NMR (500 MHz, $CDCl_3$) δ 7.23-7.38 (m, 13H), 7.08-7.12 (m, 2H), 5.50 (d, *J* = 5.0 Hz, 1H), 4.96 (d, *J* = 11.5 Hz, 1H), 4.85 (d, *J* = 11.0 Hz, 1H), 4.74 (d, *J* = 10.5 Hz, 1H), 4.60 (d, *J* = 12.0 Hz, 1H), 4.50-4.58 (m, 3H), 4.38 (d, *J* = 9.5 Hz, 1H), 4.27 (dd, *J* = 4.5, 2.0 Hz, 1H), 4.19 (d, *J* = 8.0 Hz, 1H), 3.83-3.92 (m, 3H), 3.66-3.77 (m, 3H), 3.60 (t, *J* = 9.5 Hz, 1H), 3.54 (t, *J* = 8.75 Hz, 1H), 3.49 (ddd, *J* = 9.5, 5.0, 1.5 Hz, 1H), 2.77 (dq, *J* = 13.0, 7.5 Hz, 1H), 2.71 (dq, *J* = 12.5, 7.5 Hz, 1H), 1.49 (s, 3H), 1.42 (s, 3H), 1.29-1.34 (m, 6H), 1.29 (s, 3H), 0.24 (s, 3H), 0.20 (s, 3H); ^{13}C NMR (100 MHz, $CDCl_3$) δ 138.8, 138.2, 138.0, 128.33, 128.31, 128.2, 127.92, 127.7, 127.5, 127.2, 108.9, 108.4, 96.2, 87.1, 86.2, 79.1, 78.1, 74.9, 74.5, 73.4, 70.8, 70.54, 70.48, 69.1, 68.0, 61.5, 26.1, 26.0, 24.9, 24.7, 24.3, 15.1, -1.9, -2.0; IR (film, cm^{-1}) 3063, 3030, 2978, 2930, 1454, 1382, 1256, 1211, 1070, 1000; HRMS (ES) *m/z* calcd for $C_{43}H_{58}O_{11}SSi$ $[M+Na]^+$ 833.3367, found 833.3408.

Sugar Silane 1b and 1,2:3,4-*O*-Diisopropylidene-D-Galactopyranose Glycoside (5d)

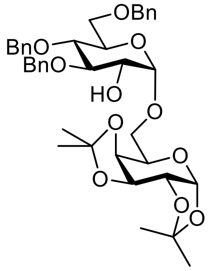

Following general procedure D, compound **4d** (58 mg, 0.072 mmol), NIS (21 mg, 0.093 mmol), TMSOTf (16 µL, 0.086 mmol), and 2,6-DTBMP (29 mg, 0.14 mmol) were stirred at -40 °C for 5 min, warmed to 0 °C and stirred for 1 h, and quenched with TBAF. The product (42 mg, 0.061 mmol, 84%) was obtained as a colorless amorphous solid upon purification by flash chromatography (30 to 45% EtOAc/hex) on SiO_2. ^{1}H NMR (500 MHz, $CDCl_3$) δ 7.25-7.42 (m, 13H), 7.13-7.17 (m, 2H), 5.53 (d, *J* = 5.0 Hz, 1H), 4.99 (d, *J* = 11.0 Hz, 1H), **4.94 (d, *J* = 3.5 Hz, 1H),** 4.83 (d, *J* = 11.0 Hz, 1H), 4.82 (d, *J* = 11.0 Hz, 1H), 4.62-4.67 (m, 2H), 4.50 (d, *J* = 12.0 Hz, 1H), 4.49 (d, *J* = 10.5 Hz, 1H), 4.34 (dd, *J* = 5.0, 2.5 Hz, 1H), 4.25 (dd, *J* = 8.0, 1.5 Hz, 1H), 4.00 (td, *J* = 6.5, 1.5 Hz, 1H), 3.92 (dd, *J* = 10.0, 6.5 Hz, 1H), 3.86 (dt, *J* = 10.0, 2.0 Hz, 1H),

3.63-3.79 (m, 6H), 2.46 (d, J = 9.0 Hz, 1H), 1.54 (s, 3H), 1.46 (s, 3H), 1.36 (s, 3H), 1.34 (s, 3H); ^{13}C NMR (100 MHz, $CDCl_3$) δ 138.8, 138.3, 138.0, 128.4, 128.3, 127.93, 127.90, 127.86, 127.65, 127.62, 127.54, 109.48, 108.7, 99.2, 96.2, 83.4, 77.1, 75.2, 75.0, 73.5, 73.2, 71.0, 70.8, 70.7, 70.5, 68.4, 67.1, 65.7, 26.1, 25.9, 24.9, 24.5; IR (film, cm^{-1}) 3458, 3064, 3031, 2987, 2932, 1383, 1256, 1211, 1070; HRMS (ES) *m/z* calcd for $C_{39}H_{48}O_{11}$ $[M+Na]^+$ 715.3094, found 715.3124.

Sugar Silane 1b and Glycoside 5b Silyl-Linked (4e)

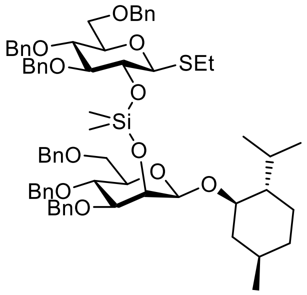

Following general procedure C, **1b** (27 mg, 0.049 mmol), **5b** (29 mg, 0.049 mmol) and $B(C_6F_5)_3$ (2 mg, 0.004 mmol) were stirred at rt overnight. The product (38 mg, 0.033 mmol, 68%) was obtained as an oil upon purification by flash chromatography (10% EtOAc/hex) on SiO_2. ^{1}H NMR (500 MHz, $CDCl_3$) δ 7.39-7.42 (m, 2H), 7.35-7.38 (m, 2H), 7.18-7.34 (m, 22H), 7.12-7.15 (m, 2H), 7.06-7.10 (m, 2H), 5.22 (d, J = 11.0 Hz, 1H), 4.80 (d, J = 12.0 Hz, 1H), 4.73 (d, J = 11.0 Hz, 1H), 4.70 (d, J = 11.0 Hz, 1H), 4.69 (d, J = 11.0 Hz, 1H), 4.60 (d, J = 12.0 Hz, 1H), 4.55 (d, J = 11.0 Hz, 1H), 4.53 (d, J = 12.0 Hz, 1H), 4.46 (d, J = 12.0 Hz, 1H), 4.423 (d, J = 12.0 Hz, 1H), 4.421 (d, J = 10.0 Hz, 1H), 4.39 (s, 1H), 4.36 (d, J = 9.5 Hz, 1H), 4.23 (d, J = 11.0 Hz, 1H), 4.17 (d, J = 2.5 Hz, 1H), 3.96 (dd, J = 9.5, 8.5 Hz, 1H), 3.73 (dd, J = 10.5, 1.5 Hz, 1H), 3.44-3.66 (m, 8H), 3.32-3.38 (m, 1H), 3.28 (dd, J = 10.5, 7.0 Hz, 1H), 2.73 (dq, J = 13.0, 7.5 Hz, 1H), 2.68 (dq, J = 12.5, 7.5 Hz, 1H), 2.57 (septd, J = 6.5, 2.0 Hz, 1H), 1.96 (d, J = 12.5 Hz, 1H), 1.60-1.68 (m, 2H), 1.27-1.40 (m, 1H), 1.28 (t, J = 7.5 Hz, 3H), 1.16-1.24 (m, 1H), 0.77-1.03 (m, 3H), 0.904 (d, J = 7.0 Hz, 3H), 0.900 (d, J = 6.5 Hz, 3H), 0.84 (d, J = 7.0 Hz, 3H), 0.31 (s, 3H), 0.28 (s, 3H); ^{13}C NMR (100 MHz, $CDCl_3$) δ 139.3, 138.60, 138.56, 138.5, 138.32, 138.31, 128.3, 128.2, 128.1, 128.0, 127.8, 127.7, 127.6, 127.53, 127.46, 127.42, 127.36, 127.2, 96.7, 87.4, 86.2, 82.4, 79.1, 78.0, 76.3, 75.6, 75.4, 75.1, 75.0, 74.8, 74.6, 73.3, 71.2, 70.7, 70.1, 69.3, 47.9, 40.9, 34.4, 31.3, 24.7, 24.5, 22.8, 22.3, 21.6, 15.8, 15.2, -0.7, -1.1; IR (film, cm^{-1}) 3062, 3030, 2953, 2918, 2850, 1652, 1454, 1368, 1254, 1095, 1067, 872, 849, 792, 735, 697; HRMS (ES) *m/z* calcd for $C_{68}H_{86}O_{11}SSi$ $[M+NH_4]^+$ 1156.5998, found 1156.5991.

Sugar Silane 1b and Glycoside 5b Glycoside (5e)

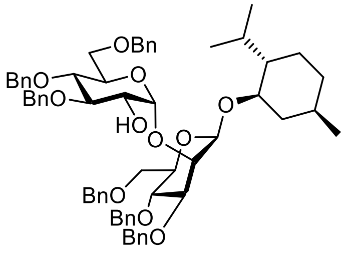

Following general procedure D, compound **4e** (37 mg, 0.032 mmol), NIS (9.5 mg, 0.042 mmol), TMSOTf (7.0 µL, 0.039 mmol), and 2,6-DTBMP (13 mg, 0.065 mmol) were stirred at -40 °C for 10 min, warmed to 0 °C and stirred for 1.5 h, and quenched with TBAF. The product (29 mg, 0.028 mmol, 89%) was obtained as a colorless oil upon purification by flash chromatography (20% EtOAc/hex) on SiO_2. ^{1}H NMR (500 MHz, $CDCl_3$) δ 7.23-7.39 (m, 27H), 7.16-7.21 (m, 3H), **5.12 (d, *J* = 3.0 Hz, 1H)**, 4.90 (d, *J* = 11.0 Hz, 1H), 4.85 (d, *J* = 10.5 Hz, 1H), 4.83 (d, *J* = 10.5 Hz, 1H), 4.76 (d, *J* = 11.5 Hz, 1H), 4.72 (d, *J* = 11.5 Hz, 1H), 4.61-4.70 (m, 4H), 4.56 (d, *J* = 12.0 Hz, 1H), 4.54 (d, *J* = 11.0 Hz, 1H), 4.49 (d, *J* = 12.0 Hz, 1H), 4.41 (s, 1H), 4.27 (d, *J* = 9.5 Hz, 1H), 3.92 (d, *J* = 2.5 Hz, 1H), 3.83 (t, *J* = 9.75 Hz, 1H), 3.70-3.81 (m, 6H), 3.58-3.64 (m, 2H), 3.44 (td, *J* = 11.0, 4.0 Hz, 1H), 3.38 (dd, *J* = 9.5, 4.5 Hz, 1H), 3.15 (d, *J* = 7.5 Hz, 1H), 2.30 (sept, *J* = 6.5 Hz, 1H), 1.91 (d, *J* = 12.5 Hz, 1H), 1.58-1.70 (m, 2H), 1.28-1.40 (m, 1H), 1.05-1.12 (m, 1H) 0.73-1.00 (m, 3H), 0.91 (d, *J* = 6.5 Hz, 3H), 0.82 (d, *J* = 7.5 Hz, 3H), 0.77 (d, *J* = 6.5 Hz, 3H); ^{13}C NMR (100 MHz, $CDCl_3$) δ 139.3, 138.9, 138.5, 138.1, 138.0, 137.3, 128.6, 128.4, 128.32, 128.28, 128.25, 128.17, 128.1, 128.0, 127.90, 127.88, 127.87, 127.8, 127.60, 127.56, 127.4, 127.34, 127.26, 100.9, 96.9, 83.9, 82.3, 76.9, 75.6, 75.2, 75.1, 74.9, 74.7, 74.1, 73.6, 73.5, 72.8, 70.5, 69.7, 68.5, 48.0, 41.0, 34.3, 31.4, 24.8, 22.7, 22.2, 21.1, 15.7; IR (film, cm^{-1}) 3428, 3031, 2923, 2867, 1727, 1453, 1273, 1095, 1054; HRMS (ES) *m/z* calcd for $C_{64}H_{76}O_{11}$ $[M+Na]^+$ 1043.5285, found 1043.5297.

Sugar Silane 1a and (1*S*,2*S*,5*S*)-(-)-2-Hydroxy-3-Pinanone (4f)

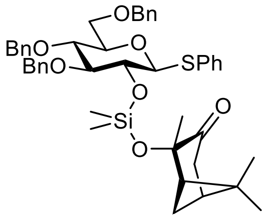

Following general procedure B, **1a** (60 mg, 0.10 mmol), (1*S*,2*S*,5*S*)-(-)-2-hydroxy-3-pinanone (17 mg, 0.10 mmol), and $B(C_6F_5)_3$ (1 mg, 0.002 mmol) were stirred for 80 min at rt. The product (70 mg, 0.091 mmol, 91%) was obtained as a colorless oil upon purification by flash chromatography (8% EtOAc/hex) on SiO_2. ^{1}H NMR (500 MHz, $CDCl_3$) δ 7.53-7.56 (m, 2H), 7.21-7.36 (m, 16H), 7.08-7.13 (m, 2H), 4.95 (d, *J* = 11.5 Hz, 1H), 4.86 (d, *J* = 11.5 Hz, 1H), 4.73 (d, *J* = 11.0 Hz, 1H), 4.63 (d, *J* = 9.5 Hz, 1H), 4.59 (d, *J* = 12.0 Hz, 1H), 4.537 (d, *J* = 10.5 Hz, 1H), 4.536 (d, *J* = 11.5 Hz, 1H), 3.82 (t, *J* = 9.0 Hz, 1H), 3.78 (dd, *J* = 11.0, 1.5 Hz, 1H), 3.61 (t, *J* = 9.0 Hz, 1H), 3.57 (t, *J* = 8.5 Hz, 1H), 3.52 (ddd, *J* = 9.0, 5.0, 1.5 Hz, 1H), 2.55 (dt, *J* = 19.0, 3.0 Hz, 1H), 2.48 (dd, *J* = 19.0, 2.0 Hz, 1H), 2.32-2.38 (m, 1H), 2.14 (t, *J* = 6.5 Hz, 1H), 2.02-2.06 (m, 1H), 1.80 (d, *J* = 10.5 Hz, 1H), 1.43 (s, 3H), 1.31 (s, 3H), 0.83 (s, 3H), 0.28 (s, 3H), 0.12 (s, 3H); ^{13}C NMR (100 MHz, $CDCl_3$) δ 210.8, 138.9, 138.3, 138.0, 134.7, 131.3, 128.8, 128.33, 128.31, 128.2, 128.0, 127.7, 127.5, 127.1, 127.0, 126.9, 89.0, 87.1, 80.2, 79.1, 78.1, 75.1, 74.9, 73.8, 73.4, 69.2, 51.7, 43.5, 38.9, 38.4, 29.0, 27.5, 25.1, 22.7, 1.2, 0.7; HRMS (ES) *m/z* calcd for $C_{45}H_{54}O_7SSi$ $[M+Na]^+$ 789.3252, found 789.3245.

Sugar Silane 1a and (1*S*,2*S*,5*S*)-(-)-2-Hydroxy-3-Pinanone (5f)

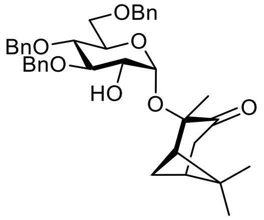

Following general procedure D, compound **4f** (58 mg, 0.076 mmol), NIS (24 mg, 0.11 mmol), TMSOTf (33 μL, 0.18 mmol), and 2,6-DTBMP (62 mg, 0.30 mmol) were stirred for 5 min at -40 °C, warmed to 0 °C and stirred for 1 h, and quenched with TBAF. The product (44 mg, 0.073 mmol, 96%) was obtained as a viscous oil upon purification by flash chromatography (20 to 30% EtOAc/hex) on SiO_2. ^{1}H NMR (500 MHz, $CDCl_3$) δ 7.25-7.39 (m, 13H), 7.16-7.19 (m, 2H), **5.27 (s, 1H)**, 4.91 (d, *J* = 11.0 Hz, 1H), 4.84 (d, *J* = 11.0 Hz, 1H), 4.82 (d, *J* = 10.5 Hz, 1H), 4.60 (d, *J* = 12.5 Hz, 1H), 4.51 (d, *J* = 10.5 Hz, 1H), 4.49 (d, *J* = 12.0 Hz, 1H), 3.98-4.02 (m, 2H), 3.63-3.72 (m, 4H), 3.52-3.57 (m, 1H), 2.69 (dd, *J* = 19.0, 1.5 Hz, 1H), 2.57 (dt, *J* = 19.0, 3.0 Hz, 1H), 2.40-2.47 (m, 1H), 2.28 (t, *J* = 6.0 Hz, 1H), 2.04-2.08 (m, 1H), 1.78-1.84 (m, 2H), 1.42 (s, 3H), 1.37 (s, 3H), 0.87 (s, 3H); ^{13}C NMR (100MHz, $CDCl_3$) δ 209.2, 138.7, 138.1, 138.0, 128.40, 128.39, 128.3, 127.9, 127.85, 127.84, 127.7, 127.64, 127.57, 92.8, 83.3, 81.8, 77.7, 75.3, 75.0, 73.5, 72.5, 71.4, 68.8, 49.9, 43.9, 39.2, 38.7, 28.8, 27.5, 22.7, 22.1; IR (film, cm^{-1}) 3443, 3064, 3031, 2925, 1722, 1453, 1273, 1070, 1027; HRMS (ES) *m/z* calcd for $C_{37}H_{44}O_7$ $[M+Na]^+$ 623.2985, found 623.2974.

Sugar Silane 2a and (1*S*,2*S*,5*S*)-(-)-2-Hydroxy-3-Pinanone (4g)

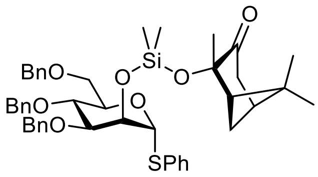

Following general procedure B, **2a** (60 mg, 0.10 mmol), (1*S*,2*S*,5*S*)-(-)-2-hydroxy-3-pinanone (17 mg, 0.10 mmol), and $B(C_6F_5)_3$ (1.0 mg, 0.0020 mmol) were stirred at rt for 85 min. The product (62 mg, 0.081 mmol, 81%) was obtained as a colorless oil upon purification by flash chromatography (8% EtOAc/hex) on SiO_2. ^{1}H NMR (500 MHz, $CDCl_3$) δ 7.50-7.53 (m, 2H), 7.38-7.41 (m, 2H), 7.20-7.37 (m, 16H), 5.54 (s, 1H), 4.88 (d, *J* = 10.5 Hz, 1H), 4.76 (d, *J* = 11.5 Hz, 1H), 4.663 (d, *J* = 11.5 Hz, 1H), 4.661 (d, *J* = 9.5 Hz, 1H), 4.56 (d, *J* = 11.0 Hz, 1H), 4.50 (d, *J* = 12.0 Hz, 1H), 4.46 (s, 1H), 4.29 (dd, *J* = 10.0, 5.0 Hz, 1H), 3.99 (t, *J* = 9.5 Hz, 1H), 3.85 (dd, *J* = 11.0, 5.0 Hz, 1H), 3.79 (dd, *J* = 9.5, 3.0 Hz, 1H), 3.76 (d, *J* = 11.0 Hz, 1H), 2.46-2.56 (m, 2H), 2.20-2.26 (m, 1H), 2.04 (t, *J* = 6.0 Hz, 1H), 1.97-2.20 (m, 1H), 1.63 (d, *J* = 11.0 Hz, 1H), 1.40 (s, 3H), 1.27 (s, 3H), 0.81 (s, 3H), 0.22 (s, 3H), 0.13 (s, 3H); ^{13}C NMR (100 MHz, $CDCl_3$) δ 210.9, 138.54, 138.47, 138.3, 134.8, 131.6, 128.9, 128.3, 128.2, 127.9, 127.8, 127.63, 127.56, 127.3, 127.2, 89.2, 80.3, 80.1, 75.0, 74.8, 73.1, 73.0, 72.0, 70.8, 69.3, 51.7, 43.4, 38.9, 28.9, 27.4, 25.2, 22.7, 0.29, 0.27; HRMS (ES) *m/z* calcd for $C_{45}H_{54}O_7SSi$ $[M+Na]^+$ 789.3257, found 789.3282.

Sugar Silane 2a and (1*S*,2*S*,5*S*)-(-)-2-Hydroxy-3-Pinanone (5g)

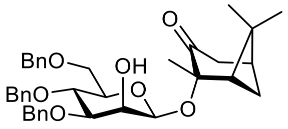

Following general procedure D, compound **4g** (53 mg, 0.069 mmol), NIS (22 mg, 0.097 mmol), TMSOTf (30 µL, 0.17 mmol), and 2,6-DTBMP (57 mg, 0.28 mmol) were stirred for 10 min at -40 °C, warmed to 0 °C and stirred for 65 min, and quenched with TBAF. The product (29 mg, 0.048 mmol, 70%) was obtained as a colorless oil upon purification by flash chromatography (20 to 30% EtOAc/hex) on SiO_2. ^{1}H NMR (500 MHz, $CDCl_3$) δ 7.25-7.39 (m, 13H), 7.20-7.23 (m, 2H), **4.96 (s, 1H),** 4.89 (d, *J* = 11.0 Hz, 1H), 4.76 (d, *J* = 11.5 Hz, 1H), 4.63 (d, *J* = 11.5 Hz, 1H), 4.61 (d, *J*, = 12.5 Hz, 1H), 4.53 (d, *J* = 11.5 Hz, 2H), 3.93 (d, *J* = 2.5 Hz, 1H), 3.81 (t, *J* = 9.5 Hz, 1H), 3.73 (dd, *J* = 11.0, 2.0 Hz, 1H), 3.68 (dd, *J* = 10.5, 5.0 Hz, 1H), 3.59 (dd, *J* = 9.0, 3.0 Hz, 1H), 3.46 (ddd, *J* = 9.5, 5.0, 2.0 Hz, 1H), 2.57-2.67 (m, 2H), 2.40-2.47 (m, 1H), 2.36 (br s, 1H), 2.31 (t, *J* = 6.0 Hz, 1H), 2.10 (sept, *J* = 3.0 Hz, 1H), 1.85 (d, *J* = 11.0 Hz, 1H), 1.48 (s, 3H), 1.37 (s, 3H), 0.88 (s, 3H); ^{13}C NMR (100 MHz, $CDCl_3$) δ 210.6, 138.39, 138.36, 137.8, 94.1, 82.0, 81.9, 75.04, 74.99, 74.1, 73.4, 71.4, 69.4, 69.2, 50.5, 43.6, 39.3, 38.5, 28.6, 27.3, 22.8, 20.6; IR (film, cm^{-1}) 3440, 3064, 3032, 2925, 1720, 1453, 1274, 1109, 1072, 1028; HRMS (ES) *m/z* calcd for $C_{37}H_{44}O_7$ $[M+Na]^+$ 623.2985, found 623.2979. The β-anomeric stereochemistry was confirmed by observation of positive NOE's at the C-2 (δ 3.93), C-3 (δ 3.59), and C-5 (δ 3.46) protons upon irradiation of the anomeric proton (δ 4.96)

Sugar Silane 1b and Ketosteroid Silyl-Linked (4h)

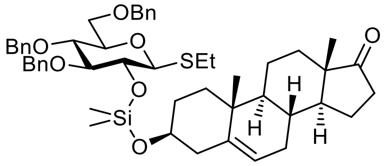

Following general procedure C, **1b** (28 mg, 0.050 mmol), steroid (14 mg, 0.050 mmol), and $B(C_6F_5)_3$ (0.5 mg, 0.001 mmol) were stirred at rt overnight. The product (39 mg, 0.046 mmol, 93%) was obtained as a colorless amorphous solid upon purification by flash chromatography (20% EtOAc/hex) on SiO_2. ^{1}H NMR (500 MHz, $CDCl_3$) δ 7.24-7.40 (m, 13H), 7.08-7.12 (m, 2H), 5.32 (d, *J* = 4.5 Hz, 1H), 4.97 (d, *J* = 11.0 Hz, 1H), 4.89 (d, *J* = 11.0 Hz, 1H), 4.73 (d, *J* = 11.0 Hz, 1H), 4.61 (d, *J* = 12.0 Hz, 1H), 4.56 (d, *J* = 12.5 Hz, 1H), 4.53 (d, *J* = 10.5 Hz, 1H), 4.40 (d, *J* = 9.5 Hz, 1H), 3.66-3.78 (m, 4H), 3.61 (t, *J* = 9.0 Hz, 1H), 3.56 (t, *J* = 8.5 Hz, 1H), 3.50 (ddd, *J* = 9.5, 5.0, 2.0 Hz, 1H), 2.77 (dq, *J* = 13.0, 7.5 Hz, 1H), 2.72 (dq, *J* = 12.5, 7.5 Hz, 1H), 2.47 (dd, *J* = 19.5, 8.5 Hz, 1H), 2.29 (d, *J* = 7.5 Hz, 2H), 2.04-2.15 (m, 2H), 1.91-1.99 (m, 1H), 1.82-1.88 (m, 1H), 1.72-1.81 (m, 2H), 1.42-1.70 (m, 6H), 1.32 (t, *J* = 7.5 Hz, 3H), 1.22-1.35 (m, 3H), 0.92-1.05 (m, 1H), 0.99 (s, 3H), 0.89 (s, 3H), 0.22 (s, 3H), 0.19 (s, 3H); ^{13}C NMR (100 MHz, $CDCl_3$) δ 221.2, 141.6, 138.8, 138.2, 137.9, 128.4, 128.3, 128.2, 128.0, 127.7, 127.6, 127.2, 127.1, 120.5, 87.0, 86.2, 79.1, 78.2, 75.1, 74.9, 74.5, 73.4, 72.0, 69.1, 51.8, 50.2, 47.5, 42.4, 37.2, 36.6, 35.8, 31.7, 31.5, 31.4, 30.8, 24.7, 21.9, 20.3, 19.4, 15.1, 13.5, -0.9, -1.2; IR

(film, cm^{-1}) 3064, 3031, 2933, 2863, 1739, 1454, 1372, 1256, 1086, 1030; HRMS (ES) *m/z* calcd for $C_{50}H_{66}O_7SSi$ $[M+Na]^+$ 861.4191, found 861.4193.

Sugar Silane 1b and Ketosteroid Glycoside (5h)

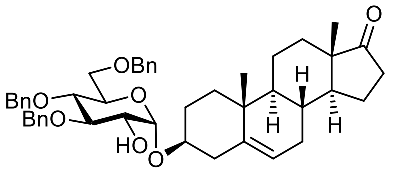

Following general procedure D, **4h** (38 mg, 0.045 mmol), NIS (14 mg, 0.063 mmol), TMSOTf (20 µL, 0.11 mmol), and 2,6-DTBMP (37 mg, 0.18 mmol) were stirred at -40 °C for 10 min, warmed to 0 °C and stirred for 1.5 h, and quenched with TBAF. The product (31 mg, 0.043 mmol, 96%) was obtained as a foamy solid upon purification by flash chromatography (30% EtOAc/hex) on SiO_2. ^{1}H NMR (500 MHz, $CDCl_3$) δ 7.38-7.42 (m, 2H), 7.26-7.37 (m, 11H), 7.15-7.18 (m, 2H), 5.34 (d, *J* = 5.0 Hz, 1H), **5.05 (d, *J* = 3.5 Hz, 1H)**, 4.98 (d, *J* = 11.0 Hz, 1H), 4.86 (d, *J* = 11.0 Hz, 1H) 4.84 (d, *J* = 10.5 Hz, 1H), 4.65 (d, *J* = 12.0 Hz, 1H), 4.51 (d, *J* = 12.0 Hz, 1H), 4.50 (d, *J* = 10.5 Hz, 1H), 3.91 (ddd, *J* = 11.5, 3.5, 2.0 Hz, 1H), 3.67-3.81 (m, 4H), 3.64 (t, *J* = 9.0 Hz, 1H), 3.54 (tt, *J* = 11.5, 5.0 Hz, 1H), 2.32- 2.52 (m, 3H), 2.04-2.18 (m, 3H), 1.83-2.00 (m, 4H), 1.44-1.72 (m, 6H), 1.22-1.34 (m, 3H), 0.97-1.11 (m, 1H), 1.04 (s, 3H), 0.90 (s, 3H). ^{13}C NMR (100 MHz, $CDCl_3$) δ 221.0, 140.7, 138.8, 138.2, 138.0, 128.4, 128.3, 127.93, 127.86, 127.83, 127.7, 127.64, 127.58, 121.3, 96.9, 83.7, 77.6, 77.5, 75.3, 75.0, 73.5, 72.9, 70.6, 68.6, 51.8, 50.2, 47.5, 40.0, 36.9, 36.8, 35.8, 31.5, 31.4, 30.8, 27.9, 21.9, 20.3, 19.4, 13.5; IR (film, cm^{-1}) 3390, 3031, 2933, 2865, 1737, 1454, 1384, 1274, 1135, 1068, 1029; HRMS (ES) *m/z* calcd for $C_{46}H_{56}O_7$ $[M+Na]^+$ 743.3924, found 743.3928.

Sugar Silane 1a and Methyl 2,3-Di-*O*-Acetyl-Glucoside Silyl-Linked (4i)

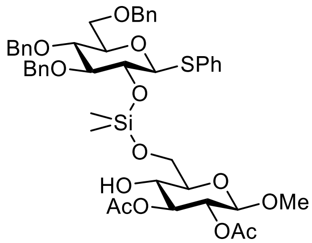

Following general procedure C, methyl 2,3-di-*O*-acetyl-glucoside[3] (28 mg, 0.10 mmol) was dissolved in a 2:1 mixture of dry CH_2Cl_2 and PhMe (0.066 M). To this solution was added $B(C_6F_5)_3$ (2.0 mg, 0.0040 mmol) followed by dropwise addition of **1a** (78 mg, 0.13 mmol) over 20 min as a solution in 1.5 mL PhMe and the reaction was stirred at rt for 10 min. The product (76 mg, 0.087 mmol, 87%, 14:1 r.r.) was obtained upon purification by flash chromatography (30 to 45% EtOAc/hex) on SiO_2. ^{1}H NMR (500 MHz, $CDCl_3$) δ 7.57-7.61 (m, 2H), 7.26-7.40 (m, 16 H), 7.12-7.16 (m, 2H), 5.04 (t, *J* = 9.5 Hz, 1H), 4.96 (d, *J* = 11.5 Hz, 1H), 4.86-4.94 (m, 2H), 4.75 (d, *J* = 10.5 Hz, 1H), 4.54-4.68 (m, 4H), 4.35 (d, *J* = 7.5 Hz, 1H), 4.08 (dd, *J* = 11.5, 4.5 Hz, 1H), 4.00 (dd, *J* = 11.5, 4.0 Hz, 1H), 3.67-3.77 (m, 3H), 3.52-3.62 (m, 2H), 3.46 (s, 3H), 3.33 (dt, *J* = 9.5, 4.5 Hz, 1H), 2.81 (br s, 1H), 2.09 (s, 3H), 2.07 (s, 3H), 0.25 (s, 3H), 0.22 (s,

3H); ^{13}C NMR (100 MHz, $CDCl_3$) δ 171.0, 169.7, 138.6, 138.2, 137.8, 133.9, 131.4, 128.9, 128.4, 128.33, 128.31, 127.9, 127.8, 127.7, 127.6, 127.4, 127.3, 126.9, 101.4, 88.7, 86.8, 79.1, 78.1, 75.4, 75.2, 74.9, 74.7, 73.8, 73.44, 71.4, 70.1, 68.9, 62.9, 56.7, 20.84, 20.78, -2.0, -2.5; IR (film, cm^{-1}) 3746, 2919, 2361, 1750, 1363, 1247, 1064, 873, 800, 739, 698; HRMS (ES) *m/z* calcd for $C_{46}H_{56}O_{13}SSi$ [M+NH_4] 894.3549, found 894.3543.

Sugar Silane 1a and Methyl 2,3-Di-*O*-Acetyl-Glucoside Glycoside (5i)

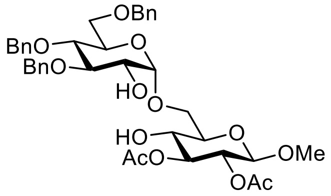

Following general procedure D, **4i** (49 mg, 0.056 mmol), NIS (18 mg, 0.078 mmol), TMSOTf (24 μL, 0.13 mmol), and 2,6-DTBMP (46 mg, 0.22 mmol) were stirred for 20 min at -40 °C, warmed to 0 °C and stirred for 40 min, and quenched with TBAF. The product (30 mg, 0.042 mmol, 75%) was obtained as a white foam upon purification by flash chromatography (50 to 70% EtOAc/hex) on SiO_2. ^{1}H NMR (500 MHz, $CDCl_3$) δ 7.26-7.40 (m, 13H), 7.12-7.15 (m, 2H), 5.06 (t, J = 9.5 Hz, 1H), 4.90-4.96 (m, 2H), **4.94 (d, J = 3.0 Hz, 1H)**, 4.86 (d, J = 11.0 Hz, 1H), 4.82 (d, J = 11.0 Hz, 1H), 4.61 (d, J = 12.0 Hz, 1H), 4.54 (d, J = 12.0 Hz, 1H), 4.49 (d, J = 11.0 Hz, 1H), 4.41 (d, J = 8.0 Hz, 1H), 4.10 (dd, J = 10.5, 4.0 Hz, 1H), 3.86 (ddd, J = 10.5, 5.0, 2.0 Hz, 1H), 3.71-3.81 (m, 4H), 3.69 (dd, J = 10.5, 2.0 Hz, 1H), 3.65 (dd, J = 10.5, 4.5 Hz, 1H), 3.50-3.59 (m, 3H), 2.55 (br s, 1H), 2.09 (s, 3H), 2.07 (s, 3H); ^{13}C NMR (100 MHz, $CDCl_3$) δ 171.3, 169.7, 138.6, 137.9, 137.6, 128.43, 128.39, 127.93, 127.91, 127.86, 127.81, 127.79, 127.7, 101.7, 98.8, 83.3, 75.7, 75.4, 75.0, 74.1, 73.4, 72.8, 71.3, 71.0, 69.8, 68.4, 67.6, 57.0, 20.9, 20.8; IR (film, cm^{-1}) 3437, 3064, 3031, 2927, 1753, 1454, 1364, 1243, 1046; HRMS (ES) *m/z* calcd for $C_{38}H_{46}O_{13}$ $[M+Na]^+$ 733.2831, found 733.2830.

Sugar Silane 1a and Methyl-3,4-*O*-(2',3'-Dimethoxybutane-2',3'-Diyl)-Mannoside Silyl-Linked (4j)

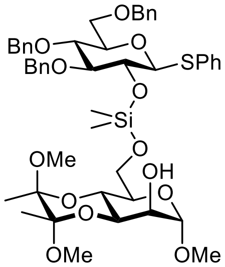

Following general procedure C, methyl-3,4-*O*-(2',3'-dimethoxybutane-2',3'-diyl)-mannoside[4] (31 mg, 0.10 mmol) was dissolved in PhMe (0.1 M). To this solution was added $B(C_6F_5)_3$ (2.0 mg, 0.0040 mmol) followed by dropwise addition of **1a** (78 mg, 0.13 mmol) over 5 min as a solution in 1 mL PhMe and the reaction was stirred at rt for 10 min. The product (45 mg, 0.050 mmol, 50%) was obtained as a white foam upon purification by flash chromatography (30 to 45% EtOAc/hex) on SiO_2. ^{1}H NMR (700 MHz, $CDCl_3$) δ 7.56-7.60 (m, 2H), 7.22-7.40

(m, 16H), 7.08-7.12 (m, 2H), 4.95 (d, *J* = 11.0 Hz, 1H), 4.89 (d, *J* = 11.5 Hz, 1H), 4.72 (d, *J* = 11.0 Hz, 1H), 4.68 (s, 1H), 4.51-4.66 (m, 4H), 3.84-4.10 (m, 5H), 3.49-3.80 (m, 7H), 3.30 (s, 3H), 3.27 (s, 3H), 3.19 (s, 3H), 2.35 (br s, 1H), 1.32 (s, 3H), 1.26 (s, 3H), 0.21 (s, 6H); ^{13}C NMR (175 MHz, $CDCl_3$) δ 138.7, 138.2, 137.9, 134.4, 131.5, 128.8, 128.4, 128.3, 128.2, 127.9, 127.74, 127.71, 127.6, 127.3, 127.2, 127.1, 100.8, 100.2, 99.7, 89.0, 87.1, 79.0, 78.0, 75.2, 74.9, 73.7, 73.4, 71.1, 69.7, 69.0, 68.2, 62.8, 61.5, 54.6, 48.0, 47.9, 17.8, 17.7, -1.6, -1.9; IR (film, cm^{-1}) 3393, 2925, 1652, 1456, 1384, 1256, 1083; HRMS (ES) *m/z* clacd for $C_{48}H_{62}O_{13}SSi$ [M+NH_4] 924.4019, found 924.4029.

Sugar Silane 1a and Methyl-3,4-*O*-(2',3'-Dimethoxybutane-2',3'-Diyl)-Mannoside Glycoside (5j)

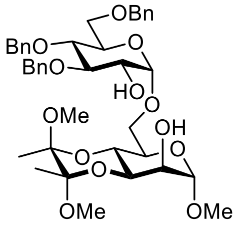

Following general procedure D, **4j** (45mg, 0.05 mmol), NIS (16 mg, 0.07 mmol), TMSOTf (22 μL, 0.12 mmol), and 2,6-DTBMP (41 mg, 0.20 mmol) were stirred at -40 °C for 5 min, warmed to 0 °C and stirred for 2.5 h, and quenched with TBAF. The product (25 mg, 0.034 mmol, 67%) was obtained as a white foam upon purification by flash chromatography (60 to 75% EtOAc/hex) on SiO_2. ^{1}H NMR (500 MHz, $CDCl_3$) δ 7.37-7.40 (m, 2H), 7.25-7.36 (m, 11H), 7.13-7.17 (m, 2H), **5.07 (d, *J* = 2.5 Hz, 1H)**, 4.96 (d, *J* = 11.5 Hz, 1H), 4.84 (d, *J* = 11.0 Hz, 1H), 4.83 (d, *J* = 11.0 Hz, 1H), 4.72 (d, *J* = 1.0 Hz, 1H), 4.63 (d, *J* = 12.5 Hz, 1H), 4.51 (d, *J* = 12.5 Hz, 1H), 4.49 (d, *J* = 11.0 Hz, 1H), 4.16 (t, *J* = 10.0 Hz, 1H), 3.95-4.03 (m, 2H), 3.81-3.92 (m, 3H), 3.70-3.80 (m, 4H), 3.68 (dd, *J* = 11.0, 2.0 Hz, 1H), 3.59-3.66 (m, 1H), 3.35 (s, 3H), 3.28 (s, 3H), 3.24 (s, 3H), 2.52 (br s, 2H), 1.33 (s, 3H), 1.29 (s, 3H); ^{13}C NMR (100 MHz, $CDCl_3$) δ 138.7, 138.4, 138.0, 128.4, 128.3, 128.0, 127.9, 127.8, 127.65, 127.59, 127.56, 101.0, 100.3, 99.9, 99.8, 83.1, 77.2, 75.1, 74.9, 73.5, 73.4, 70.7, 70.0, 69.6, 68.6, 68.0, 66.5, 63.0, 55.0, 48.1, 48.0, 17.7; IR (film, cm^{-1}) 3438, 3029, 2928, 1453, 1378, 1129, 1046; HRMS (ES) *m/z* calcd for $C_{40}H_{52}O_{13}$ [M+Na]$^+$ 763.3300, found 763.3299.

Phenyl 2-*O*-benzyl-6-*O*-*tert*-butyldimethylsilyl-3,4-*O*-(2',3'-dimethoxybutane-2',3'-diyl)-thio-β-D-glucopyranoside (14)

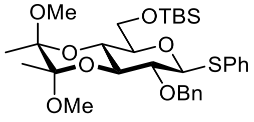

A solution of **13** (4.8 g, 9.6 mmol) and BnBr (1.37 mL, 11.5 mmol) in DMF (24 mL, 0.40 M) was cooled to 0 °C and NaH (500 mg, 21 mmol) was added. The solution was stirred at rt for 1 h and quenched with MeOH, diluted with EtOAc (100 mL), washed with H_2O (5 x 70 mL), dried over $MgSO_4$, filtered, and concentrated. The product (5.2 g, 8.9 mmol, 92%) was obtained without further purification as a white foam. ^{1}H NMR (700 MHz, $CDCl_3$) δ 7.54-7.58 (m, 2H),

7.39-7.44 (d, J = 7.0 Hz, 2H), 7.30-7.35 (t, J = 7.0 Hz, 2H), 7.23-7.29 (m, 4H), 4.80 (d, J = 10.5 Hz, 1H), 4.69 (d, J = 10.5 Hz, 1H), 4.61 (d, J = 9.1 Hz, 1H), 3.80-3.90 (m, 3H), 3.76 (t, J = 9.8 Hz, 1H), 3.46 (t, J = 9.8 Hz, 1H), 3.42 (d, J = 9.1 Hz, 1H), 3.28 (s, 3H), 3.26 (s, 3H), 1.34 (s, 3H), 1.29 (s, 3H), 0.90 (s, 9H), 0.09 (s, 3H), 0.05 (s, 3H); ^{13}C NMR (175 MHz, $CDCl_3$) δ 138.5, 133.4, 132.5, 128.8, 128.3, 128.1, 127.7, 127.5, 99.6, 99.4, 87.4, 78.5, 77.4, 75.4, 75.0, 64.9, 61.2, 48.1, 47.9, 25.9, 18.4, 17.8, 17.6, -5.1, -5.45; IR (film, cm^{-1}) 3440, 2927, 1641, 1471, 1376, 1252, 1133, 1048; HRMS (ES) *m/z* calcd for $C_{31}H_{46}O_7SSi$ $[M+NH_4]^+$ 608.3072, found 608.3074.

Phenyl 2-*O*-benzyl-3,4-*O*-(2',3'-dimethoxybutane-2',3'-diyl)-thio-β-D-glucopyranoside (7a)

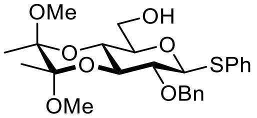

To a solution of **14** (5.1 g, 8.7 mmol) in CH_2Cl_2 (35 mL, 0.25 M) was added TBAF (1 M in THF, 13.0 mL, 13.0 mmol). The reaction was stirred at rt for 3 h and quenched with H_2O, extracted with CH_2Cl_2 (x3), the organic layers combined, dried over $MgSO_4$, filtered, and concentrated. The crude mixture was subjected to flash chromatography (20 to 40% EtOAc/hex) and recrystallized (H_2O/EtOH) to give the product (3.7 g, 7.7 mmol, 89%) as white crystals. ^{1}H NMR (500 MHz, $CDCl_3$) δ 7.50-7.54 (m, 2H), 7.43-7.47 (m, 2H), 7.34-7.39 (m, 2H), 7.29-7.34 (m, 2H), 4.87 (d, J = 10.5 Hz, 1H), 4.77 (d, J = 11.0 Hz, 1H), 4.70 (d, J = 9.5 Hz, 1H), 3.87-3.94 (m, 2H), 3.74 (dd, J = 12.0, 5.0 Hz, 1H), 3.71 (t, J = 10 Hz, 1H), 3.55 (ddd, J = 5.0, 4.5, 3.0 Hz, 1H), 3.49 (t, J = 9.5 Hz, 1H), 3.29 (s, 3H), 3.29 (s, 3H), 1.89 (s, 1H), 1.37 (s, 3H), 1.32 (s, 3H); ^{13}C NMR (100 MHz, $CDCl_3$) δ 138.2, 132.7, 132.6, 129.0, 128.3, 128.2, 127.9, 127.8, 99.7, 99.5, 87.2, 77.6, 77.5, 75.5, 74.6, 65.8, 61.5, 48.02, 47.95, 17.8, 17.6; IR (film, cm^{-1}) 3502, 2948, 2360, 1652, 1456, 1377, 1132; HRMS (ES) *m/z* calcd for $C_{25}H_{32}O_7S$ $[M+NH_4]^+$ 494.2207, found 494.2213.

Sugar Silane (8a)

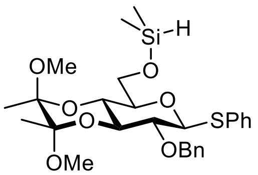

Following general procedure A, **7a** (1.04 g, 2.18 mmol), NEt_3 (0.61 mL, 4.3 mmol), and Me_2SiHCl (0.36 mL, 3.3 mmol) were stirred at 0 °C for 3 h. The product (1.13 g, 2.11 mmol, 98%) was obtained as a white solid after aqueous workup. ^{1}H NMR (700 MHz, $CDCl_3$) δ 7.55-7.59 (m, 2H), 7.42 (d, J = 7.0 Hz, 2H), 7.33 (t, J = 7.0 Hz, 2H), 7.23-7.30 (m, 4H), 4.80 (d, J = 10.5 Hz, 1H), 4.71 (d, J = 10.5 Hz, 1H), 4.67 (sep, J = 9.8 Hz, 1H), 4.61 (d, J = 9.1 Hz, 1H), 3.81-3.92 (m, 3H), 3.73 (t, J = 9.8 Hz, 1H), 3.43-3.50 (m, 2 H), 3.27 (s, 3H), 3.26 (s, 3H), 1.34 (s, 3H), 1.29 (s, 3H), 0.22 (d, J = 2.8 Hz, 3H), 0.21 (d, J = 2.8 Hz, 3H); ^{13}C NMR (175 MHz, $CDCl_3$) δ 138.4, 133.1, 132.7, 128.8, 128.3, 128.1, 127.7, 127.6, 99.6, 99.5, 87.2, 78. 2, 77.4, 75.4, 74.8, 65.1, 62.4, 48.0, 47.9, 17.8, 17.6, -1.37, -1.40; IR (film, cm^{-1}) 2992, 2950, 2835, 2115, 1454, 1376, 1252, 1133, 1077, 1048; HRMS (ES) *m/z* calcd for $C_{27}H_{38}O_7SSi$ $[M+Na]^+$ 557.2000, found 557.2006.

Phenyl 2-*O*-acetyl-6-*O*-*tert*-butyldimethylsilyl-3,4-*O*-(2',3'-dimethoxybutane-2',3'-diyl)-thio-β-D-glucopyranoside (15)

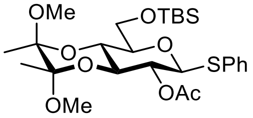

To a solution of **13** (1.66 g, 3.32 mmol) in pyridine (6.6 mL, 0.5 M) was added acetic anhydride (6.6 mL, 0.50 M) and the reaction was stirred overnight. The reaction was diluted with ethyl acetate, washed with H_2O, washed with sat. aq. NH_4Cl (x3), dried over $MgSO_4$, filtered, and concentrated. The crude mixture was subjected to flash chromatography (15 to 30% EtOAc/hex) to give the product (1.69 g, 3.11 mmol, 94%) as a white solid. ^1H NMR (500 MHz, $CDCl_3$) δ 7.49-7.54 (m, 2H), 7.25-7.31 (m, 3H), 4.97 (t, J = 9.5 Hz, 1H), 4.65 (d, J = 9.5 Hz, 1H), 3.89 (dd, J = 11.5, 2 Hz, 1H), 3.74-3.86 (m, 3H), 3.48 (ddd, J = 9.5, 4.5, 2 Hz, 1H), 3.25 (s, 3H), 3.24 (s, 3H), 2.12 (s, 3H), 1.28 (s, 3H), 1.27 (s, 3H), 0.90 (s, 9H), 0.09 (s, 3H), 0.07 (s, 3H); ^{13}C NMR (175 MHz, $CDCl_3$) δ 169.2, 133.1, 132.3, 128.8, 127.7, 99.7, 99.5, 86.4, 78.8, 72.1, 69.3, 65.0, 61.3, 48.0, 47.6, 25.9, 20.9, 18.3, 17.61, 17.57, -5.08, -5.45; IR (film, cm^{-1}) 2987, 2950, 2855, 1744, 1584, 1464, 1367, 1277, 1242, 1071; HRMS (ES) *m/z* calcd for $C_{26}H_{42}O_8SSi$ $[M+NH_4]^+$ 560.2708, found 560.2721.

Phenyl 2-*O*-acetyl-3,4-*O*-(2',3'-dimethoxybutane-2',3'-diyl)-thio-β-D-glucopyranoside (7b)

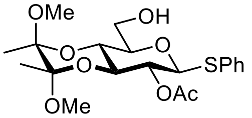

To a solution of **15** (1.69 g, 3.11 mmol) in CH_2Cl_2 (10 mL, 0.31 M) was added TBAF (1.0 M in THF, 9.3 mL, 9.3 mmol) and the reaction was stirred for 3 h. The reaction was poured into H_2O and extracted with CH_2Cl_2 (x3), dried over $MgSO_4$, filtered, and concentrated. The crude mixture was subjected to flash chromatography (20 to 30% EtOAc/hex) to give the product (1.07 g, 2.50 mmol, 80%) as a colorless amorphous solid. ^1H NMR (400 MHz, $CDCl_3$) δ 7.38-7.46 (m, 2H), 7.19-7.29 (m, 3H), 4.92 (t, J = 9.6 Hz, 1H), 4.64 (d, J = 9.6 Hz, 1H), 3.74-3.88 (m, 2H), 3.65 (t, J = 9.6 Hz, 2H), 3.47-3.56 (m, 1H), 3.18 (s, 3H), 3.16 (s, 3H), 2.29 (br s, 1H), 2.06 (s, 3H), 1.207 (s, 3H), 1.205 (s, 3H); ^{13}C NMR (175 MHz, $CDCl_3$) δ 169.1, 132.4, 132.3, 128.9, 127.9, 99.7, 99.5, 86.1, 78.1, 71.6, 69.2, 65.6, 61.2, 47.8, 47.5, 20.8, 17.54, 17.48; IR (film, cm^{-1}) 3509, 2994, 2951, 2836, 2249, 1754, 1585, 1440, 1370, 1224, 1134, 1035; HRMS (ES) *m/z* calcd for $C_{20}H_{28}O_8S$ $[M+NH_4]^+$ 451.1408, found 451.1408.

Sugar Silane (8b)

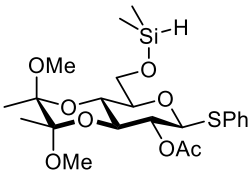

Following general procedure A, **7b** (1.04 g, 2.43 mmol), NEt_3 (0.61 mL, 4.3 mmol), and Me_2SiHCl (0.36 mL, 3.3 mmol) were stirred at 0 °C for 3 h. The product (1.13g, 2.32 mmol, 98%) was obtained as a white solid without further purification. ^1H NMR (700 MHz, $CDCl_3$) δ 7.48-7.52 (m, 2H), 7.25-7.29 (m, 3H), 4.95 (t, *J* = 9.8 Hz, 1H), 4.64 (sep, *J* = 2.8 Hz, 1H), 4.62 (d, *J* = 9.8 Hz, 1H), 3.89 (dd, *J* = 11.2, 1.4 Hz, 1H), 3.78-3.84 (m, 2H), 3.72 (t, *J* = 9.8 Hz, 1H), 3.50 (ddd, *J* = 9.8, 5.6, 1.4 Hz, 1H), 3.22 (s, 3H), 3.21 (s, 3H), 2.10 (s, 3H), 1.25 (s, 3H), 1.24 (s, 3H), 0.20 (d, *J* = 2.8 Hz, 3H), 0.19 (d, *J* = 2.8 Hz, 3H); ^{13}C NMR (175 MHz, $CDCl_3$) δ 169.1, 132.7, 132.6, 128.7, 127.8, 99.8, 99.5, 86.2, 78.6, 71.9, 69.3, 65.2, 62.4, 47.9, 47.6, 20.9, 17.60, 17.55, -1.39, -1.41; IR (film, cm^{-1}) 2992, 2951, 2833, 2113, 1752, 1368, 1223, 1127, 1066; HRMS (ES) *m/z* calcd for $C_{22}H_{34}O_8SSi$ $[M+Na]^+$ 509.1636, found 509.1636.

Phenyl 3,4,6-*O*-triacetyl-2-azido-2-deoxy-thio-β-D-glucopyranoside (16)

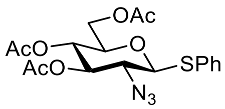

To a solution of the corresponding 2-deoxy-2-amino-thioglycoside[5] (1.03 g, 3.78 mmol), K_2CO_3 (1.04 g, 7.55 mmol), and $CuSO_4•5H_2O$ (10 mg, 0.038 mmol) in MeOH (38 mL, 0.10 M) was added imidazole-1-sulfonyl azide (950 mg, 4.53 mmol) and the reaction was stirred overnight. The reaction was concentrated and azeotroped with toluene (x2), dissolved in pyridine (19 mL, 0.2 M), and acetic anhydride (2.8 mL, 30 mmol) was added and the reaction was stirred overnight. The reaction was diluted with ethyl acetate, washed with H_2O, washed with sat. aq. NH_4Cl (x3), dried over $MgSO_4$, filtered, and concentrated. The crude mixture was subjected to flash chromatography (20 to 30% EtOAc/hex) to give the product (376 mg, 0.888 mmol, 23%) as a white solid. ^1H NMR (500 MHz, $CDCl_3$) δ 7.48-7.53 (m, 2H), 7.23-7.30 (m, 3H), 5.02 (t, *J* = 9.5 Hz, 1H), 4.84 (t, *J* = 9.5 Hz, 1H), 4.48 (d, *J* = 10.5 Hz, 1H), 4.06-4.19 (m, 2H), 3.65 (ddd, *J* = 10.0, 5.0, 2.5 Hz, 1H), 3.33 (t, *J* = 10.0 Hz, 1H), 1.99 (s, 3H), 1.97 (s, 3H), 1.92 (s, 3H); ^{13}C NMR (175 MHz, $CDCl_3$) δ 170.3, 169.7, 169.5, 133.8, 130.3, 129.0, 128.7, 85.4, 75.5, 74.2, 68.0, 62.5, 61.9, 20.6, 20.5, 20.4; IR (film, cm^{-1}) 2110, 1744, 1439, 1365, 1221, 1047; HRMS (ES) *m/z* calcd for $C_{18}H_{21}N_3O_7S$ $[M+NH_4]^+$ 441.1438, found 441.1439.

Phenyl 2-azido-2-deoxy-thio-β-D-glucopyranside (17)

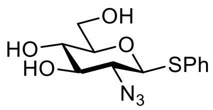

To a solution of **16** (376 mg, 0.888 mmol) in methanol (9 mL, 0.1 M) was added NaOMe (5 mg, 0.09 mmol) and the reaction was stirred overnight. The reaction was concentrated to give the product (256 mg, 0.861 mmol, 97%) without further purification as a white solid. ^1H NMR (400 MHz, CD_3OD) δ 7.55-7.61 (m, 2H), 7.26-7.36 (m, 3H), 4.54 (d, *J* = 10.0 Hz, 1H), 3.86 (dd, *J* = 12.0, 1.6 Hz, 1H), 3.65-3.72 (m, 1H), 3.38-3.45 (m, 1H), 3.26-3.36 (m, 1H), 3.14 (t, 1H); ^{13}C NMR (100 MHz, CD_3OD) δ 136.4, 136.2, 132.6, 131.6, 89.8, 84.7, 81.0, 73.7, 69.6, 65.2; IR (film, cm^{-1}) 3344, 2918, 2110, 1584, 1439, 1352, 1273, 1068, 1024; HRMS (ES) *m/z* calcd for $C_{12}H_{15}N_3O_4S$ $[M+Cl]^-$ 332.0477, found 332.0473.

Phenyl 2-azido-2-deoxy-3,4-*O*-(2',3'-dimethoxybutane-2',3'-diyl)-thio-β-D-glucopyranoside (7c)

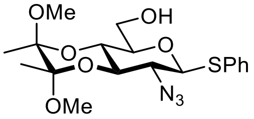

To a solution of **17** (256 mg, 0.861 mmol), 2,3-butadione (83 μL, 0.95 mmol), and $CH(OCH_3)_3$ (310 μL, 2.84 mmol) in methanol (9 mL, 0.1 M) was added camphorsulfonic acid (40 mg, 0.17 mmol) and the reaction was refluxed overnight, quenched with NEt_3, and concentrated. The crude mixture was subjected to flash chromatography (15 to 18% EtOAc/hex) and recrystallized (H_2O/EtOH) to give the product (204 mg, 0.496 mmol, 58%) as a white solid. ^{1}H NMR (400MHz, $CDCl_3$) δ 7.31-7.38 (m, 2H), 7.10-7.17 (m, 3H), 4.25 (d, *J* = 9.6 Hz, 1H), 3.68 (d, *J* = 12.0 Hz, 1H), 3.47-3.58 (m, 2H), 3.44 (t, *J* = 9.6 Hz, 1H), 3.32 (ddd, *J* = 9.6, 4.8, 2.4 Hz, 1H), 3.20 (t, *J* = 10.0 Hz, 1H), 3.12 (s, 3H), 3.04 (s, 3H), 1.90 (s, 1H), 1.13 (s, 3H), 1.08 (s, 3H); ^{13}C NMR (100 MHz, $CDCl_3$) δ 133.7, 130.7, 129.1, 128.6, 100.2, 99.7, 86.1, 78.1, 72.9, 65.6, 61.4, 61.2, 48.0, 17.6, 17.5; IR (film, cm^{-1}) 3500, 2992, 2948, 2833, 2223, 2108, 1582, 1474, 1439, 1368, 1322, 1277, 1220, 1113, 1048, 1030; HRMS (ES) *m/z* calcd for $C_{18}H_{25}N_3O_6S$ $[M+NH_4]^+$ 429.1802, found 429.1800.

Sugar Silane (9)

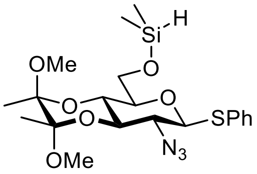

Following general procedure A, **7c** (204 mg, 0.496 mmol), NEt_3 (138 μL, 0.990 mmol), and Me_2SiHCl (83 μL, 0.74 mmol) were stirred at 0 °C for 5 h. The product (222 mg, 0.447 mmol, 90%) was obtained as a white solid after aqueous workup. ^{1}H NMR (400 MHz, $CDCl_3$) δ 7.54-7.62 (m, 2H), 7.24-7.34 (m, 3H), 4.66 (quin, *J* = 2.8 Hz, 1H), 4.37 (d, *J* = 9.6 Hz, 1H), 3.77-3.92 (m, 2H), 3.63-3.76 (m, 2H), 3.42-3.50 (m, 1H), 3.71 (t, *J* = 9.6 Hz, 1H), 3.30 (s, 3H), 3.23 (s, 3H), 1.30 (s, 3H), 1.25 (s, 3H), 0.20 (t, *J* = 2.4 Hz, 6H); ^{13}C NMR (100 MHz, $CDCl_3$) 134.0, 130.9, 128.9, 128.4, 100.1, 99.7, 86.0, 78.5, 73.1, 65.0, 62.2, 61.3, 48.0, 17.6, 17.5, -1.40, -1.44; IR (film, cm^{-1}) 2943, 2837, 2110, 1438, 1375, 1366, 1274, 1250, 1111, 1030, 896; HRMS (ES) *m/z* calcd for $C_{20}H_{31}N_3O_6SSi$ $[M+NH_4]^+$ 487.2041, found 487.2035.

Phenyl 2-deoxy-3,4-*O*-(2',3'-dimethoxybutane-2',3'-diyl)-thio-α,β-D-glucopyranoside (7d)

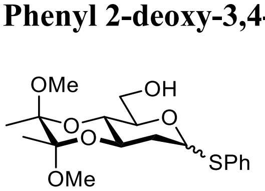

To a solution of 2-deoxy-thioglycoside[6] (2.23 g, 8.70 mmol), 2,3-butadione (840 μL, 9.60 mmol), and $CH(OCH_3)_3$ (3.2 mL, 29 mmol) in methanol (87 mL, 0.10 M) was added

camphorsulfonic acid (400 mg, 1.7 mmol) and the reaction was refluxed overnight, quenched with NEt_3, and concentrated. The crude mixture was subjected to flash chromatography (5 to 35% EtOAc/hex) to give the product (2.26 g, 6.10 mmol, 70%) as a colorless foam. ^{1}H NMR (700 MHz, $CDCl_3$) δ 7.44-7.49 (m, 3.1H), 7.25-7.33 (m, 4.7H), 5.64-5.67 (m, 1H, *α*-anomer), 4.88 (dd, *J* = 11.2, 1.4 Hz,, 0.56H, *β*-anomer), 4.27 (td, *J* = 11.2, 1.4 Hz, 1H, *α*-anomer), 4.13-4.19 (m, 1.2H), 3.83-3.90 (m, 1.2H), 3.72-3.82 (m, 2.8H), 3.66 (t, *J* = 10.5 Hz, 1H, *α*-anomer), 3.58 (t, *J* = 9.8 Hz, 0.56H, *β*-anomer), 3.52 (ddd, *J* = 9.8, 4.9, 2.8 Hz, 0.56H, *β*-anomer), 3.33 (s, 3H, *α*-anomer), 3.28 (s, 3H, *α*-anomer), 3.27 (s, 1.7H, *β*-anomer), 3.25 (s, 1.7H, *β*-anomer), 2.20-3.23 (m, 2H, *α*-anomer), 2.18 (ddd, *J* = 12.6, 4.2, 2.1 Hz, 0.56H, *β*-anomer), 1.96 (dd, *J* = 7.0, 5.6 Hz, 0.56H, *β*-anomer), 1.86 (q, *J* = 11.9 Hz, 0.56H, *β*-anomer), 1.69 (dd, *J* = 7.7, 5.6 Hz, 1H, *α*-anomer), 1.33 (s, 3H, *α*-anomer), 1.32 (s, 3H, *α*-anomer), 1.30 (s, 1.7H, *β*-anomer), 1.29 (s, 1.7H, *β*-anomer); ^{13}C NMR (175 MHz, $CDCl_3$) δ 134.4, 133.0, 131.93, 131.91, 129.0, 127.8, 127.5, 100.0, 99.91, 99.89, 84.3, 82.5, 77.9, 70.9, 68.8, 68.4, 68.0, 65.6, 61.7, 61.3, 48.1, 48.0, 47.92, 47.91, 36.0, 35.3, 17.8, 17.74, 17.69; IR (film, cm^{-1}) 3500, 2944, 2354, 1457, 1379, 1126, 1048, 918, 739, 678; HRMS (ES) *m/z* calcd for $C_{18}H_{26}O_6S$ $[M+Na]^+$ 393.1342, found 393.1347.

Sugar Silane (10)

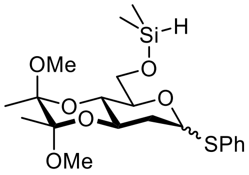

Following general procedure A, **7d** (2.3 mg, 6.1 mmol), NEt_3 (1.7 mL, 12 mmol), and Me_2SiHCl (1.0 mL, 9.2 mmol) were stirred at 0 °C for 4 h. The product (2.48 g, 5.79 mmol, 95%) was obtained as a purple oil after aqueous workup. ^{1}H NMR (700 MHz, $CDCl_3$) δ 7.51-7.53 (m, 1.0H), 7.44-7.47 (m, 1.9H), 7.24-7.29 (m, 3.9H), 7.21-7.24 (m, 1.0H), 5.68 (d, *J* = 4.9 Hz, 1H, *α*-anomer), 4.81 (dd, *J* = 11.2, 2.1 Hz, 0.56H, *β*-anomer), 4.66 (quin, *J* = 2.8 Hz, 0.56H, *β*-anomer), 4.60 (quin, *J* = 2.8 Hz, 1H, *α*-anomer), 4.23 (ddd, *J* = 9.8, 4.2, 1.4 Hz, 1H, *α*-anomer), 4.12 (ddd, *J* = 11.9, 9.8, 4.9 Hz, 1H, *α*-anomer), 3.90 (d, *J* = 4.2 Hz, 0.56H, *β*-anomer), 3.89 (d, *J* = 5.6 Hz, 1H, *α*-anomer), 3.80-3.86 (m, 1.2H), 3.78 (dd, *J* = 11.2, 2.1 Hz, 1H, *α*-anomer), 3.70 (t, *J* = 9.8 Hz, 1H, *α*-anomer), 3.58 (t, *J* = 9.8 Hz, 0.56H, *β*-anomer), 3.45 (ddd, *J* = 9.8, 4.9, 2.1 Hz, 0.56H, *β*-anomer), 3.31 (s, 3H, *α*-anomer), 3.28 (s, 3H, *α*-anomer), 3.25 (s, 1.7H, *β*-anomer), 3.25 (s, 1.7H, *β*-anomer), 2.15-2.24 (m, 2H, *α*-anomer), 2.13 (ddd, *J* = 11.9, 4.2, 2.1 Hz, 0.56H, *β*-anomer), 1.84 (q, *J* = 11.9 Hz, 0.56H, *β*-anomer), 1.32 (s, 3H, *α*-anomer), 1.31 (s, 3H, *α*-anomer), 1.281 (s, 1.7H, *β*-anomer), 1.275 (s, 1.7H, *β*-anomer), 0.21 (d, *J* = 2.1 Hz, 1.7H, *β*-anomer), 0.20 (d, *J* = 2.8 Hz, 1.7H, *β*-anomer), 0.17 (d, *J* = 2.8 Hz, 1.7H, *β*-anomer); ^{13}C (175 MHz, $CDCl_3$) δ 135.1, 133.6, 132.1, 131.3, 128.81, 128.75, 127.5, 127.1, 99.9, 99.8, 84.3, 82.4, 78.6, 71.4, 68.7, 68.2, 67.4, 65.8, 62.8, 62.3, 48.03, 47.98, 47.9, 36.0, 35.5, 17.81, 17.78, 17.75, 17.7, -1.29, -1.34, -1.5, -1.6; IR (film, cm^{-1}) 2947, 2354, 2120, 1455, 1378, 1248, 1120, 1054, 897, 747, 685; HRMS (ES) *m/z* calcd for $C_{20}H_{32}O_6SSi$ $[M+Na]^+$ 451.1581, found 451.1583.

Sugar Silane 8a and Butanol Silyl-Linked (11a)

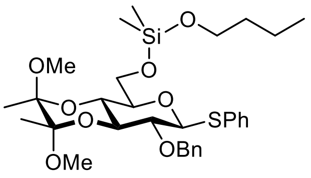

Following general procedure C, **8a** (120 mg, 0.22 mmol), butanol (18 µL, 0.2 mmol), CuCl (0.99 mg, 0.010 mmol), IPr•HCl (8.5 mg, 0.020 mmol), NaOtBu (1.9 mg, 0.020 mmol), and 4Å molecular sieves (80 mg) were stirred overnight. The product (113 mg, 0.186 mmol, 93%) was obtained as a colorless oil upon flash chromatography (5 to 12.5% EtOAc/hex) on SiO_2. ^{1}H NMR (500 MHz, $CDCl_3$) δ 7.56-7.61 (m, 2H), 7.45 (d, *J* = 7.0 Hz, 2H), 7.36 (t, *J* = 7.0 Hz, 2H), 7.26-7.33 (m, 4H), 4.83 (d, *J* = 10.5 Hz, 1H), 4.78 (d, *J* = 11.0 Hz, 1H), 4.65 (d, *J* = 9.5 Hz, 1H), 3.99 (d, *J* = 11.0 Hz, 1H), 3.85-3.93 (m, 2H), 3.77 (t, *J* = 10.0 Hz, 1H), 3.70 (t, *J* = 7.0 Hz, 2H), 3.46-3.54 (m, 2H), 3.31 (s, 3H), 3.29 (s, 3H), 1.54 (quin, *J* = 7 Hz, 2H), 1.29-1.40 (m, 2H), 1.37 (s, 3H), 1.32 (s, 3H), 0.91 (t, *J* = 7.5 Hz, 3H), 0.17 (s, 3H), 0.16 (s, 3H); ^{13}C NMR (125 MHz, $CDCl_3$) δ 138.4, 133.4, 132.4, 128.8, 128.3, 128.2, 127.8, 127.5, 99.7, 99.5, 87.2, 78.2, 77.4, 75.4, 74.9, 65.2, 62.3, 60.8, 48.0, 47.9, 34.7, 19.0, 17.9, 17.7, 13.9, -3.0, -3.2; IR (film, cm^{-1}) 2957, 1702, 1454, 1376, 1256, 1036; HRMS (ES) *m/z* calcd for $C_{31}H_{46}O_8SSi$ $[M+NH_4]^+$ 624.3021, found 624.3026.

Sugar Silane 8a and Butanol Glycoside (12a)

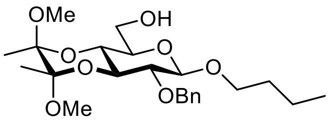

Following the general procedure, **11a** (57 mg, 0.094 mmol), NIS (27 mg, 0.12 mmol), 2,6-DTBMP (39 mg, 0.19 mmol), and TMSOTf (20 µL, 0.11 mmol) were stirred at -40 °C for 10 min, warmed to 0 °C for 1 h, and quenched with TBAF. The product (35 mg, 0.080 mmol, 85%) was obtained as a colorless oil upon flash chromatography (15 to 30% EtOAc/hex) on SiO_2. ^{1}H NMR (500 MHz, $CDCl_3$) δ 7.40 (d, *J* = 7.0 Hz, 2H), 7.33 (t, *J* = 7.0 Hz, 2 H), 7.27 (t, *J* = 7.0 Hz, 1H), 4.85 (d, *J* = 11.0 Hz, 1H), 4.79 (d, *J* = 11.5 Hz, 1H), **4.44 (d, *J* = 7.5 Hz, 1H)**, 3.93 (dt, *J* = 9.5, 6.5 Hz, 1H), 3.88 (dd, *J* = 12.0, 3.0 Hz, 1H), 3.82 (t, *J* = 10.0 Hz, 1H), 3.68-3.78 (m, 2H), 3.48-3.58 (m, 2H), 3.42 (dd, *J* = 9.5, 7.5 Hz, 1H), 3.31 (s, 3H), 3.27 (s, 3H), 1.56-1.71 (m, 2H), 1.34-1.51 (m, 2H), 1.37 (s, 3H), 1.32 (s, 3H), 0.94 (t, *J* = 7.5 Hz, 3H); ^{13}C NMR (175 MHz, $CDCl_3$) δ 138.7, 128.2, 127.7, 127.5, 103.9, 99.52, 99.47, 79.0, 74.7, 73.6, 72.2, 70.2, 66.1, 61.4, 47.9, 47.8, 31.7, 19.2, 17.8, 17.6, 13.8; IR (film, cm^{-1}) 3491, 3064, 2915, 2246, 1736, 1497, 1454, 1369, 1307; HRMS (ES) *m/z* calcd for $C_{23}H_{36}O_8$ $[M+NH_4]^+$ 458.2748, found 458.2751.

Sugar Silane 8a and Phenethyl Alcohol Silyl-Linked (11b)

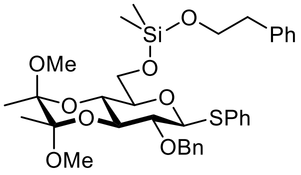

Following general procedure C, **8a** (59 mg, 0.11 mmol), phenethyl alcohol (12 μL, 0.10 mmol), CuCl (0.5 mg, 0.005 mmol), IPr•HCl (4.1 mg, 0.010 mmol), NaOtBu (1 mg, 0.01 mmol), and 4Å molecular sieves (40 mg) were stirred overnight. The product (47 mg, 0.072 mmol, 72%) was obtained as a colorless oil upon flash chromatography (5 to 10% EtOAc/hex) on SiO_2. ^1H NMR (700 MHz, $CDCl_3$) δ 7.53-7.57 (m, 2H), 7.41-7.45 (d, *J* = 7.7 Hz, 2H), 7.34 (t, *J* = 7.0 Hz, 2H), 7.22-7.30 (m, 6H), 7.17-7.21 (m, 2H), 4.81 (d, *J* = 10.5 Hz, 1H), 4.72 (d, *J* = 10.5 Hz, 1H), 4.63 (d, *J* = 9.8 Hz, 1H), 3.85-3.93 (m, 4H), 3.82 (dd, *J* = 11.2, 4.2 Hz, 1H), 3.74 (t, *J* = 9.8 Hz, 1H), 3.44-3.49 (m, 2H), 3.27 (s, 6H), 2.84 (t, *J* = 7.0 Hz, 2H), 1.35 (s, 3H), 1.29 (s, 3H), 0.093 (s, 3H), 0.089 (s, 3H); ^{13}C NMR (175MHz, $CDCl_3$) δ 138.8, 138.4, 133.4, 132.2, 129.1, 128.8, 128.3, 128.2, 128.1, 127.7, 127.4, 126.1, 99.6, 99.5, 87.2, 78.1, 77.4, 75.4, 74.8, 65.1, 63.6, 60.7, 48.0, 47.9, 39.2, 28.8, 23.8, 23.6, 17.8, 17.6, -3.1, -3.3; IR (film, cm^{-1}) 3064, 3031, 2935, 1679, 1606, 1584, 1472, 1368, 1258, 1136, 1032; HRMS (ES) *m/z* calcd for $C_{35}H_{46}O_8SSi$ $[M+NH_4]^+$ 672.3021, found 672.3021.

Sugar Silane 8a and Phenethyl Alcohol Glycoside (12b)

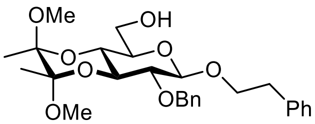

Following general procedure D, **11b** (47 mg, 0.072 mmol), NIS (21 mg, 0.093 mmol), 2,6-DTBMP (29 mg, 0.14 mmol), and TMSOTf (16 μL, 0.086 mmol) were stirred at -40 °C, warmed to 0 °C for 1 h, and quenched with TBAF. The product (30 mg, 0.061 mmol, 86%) was obtained as a colorless oil upon flash chromatography (15 to 25% EtOAc/hex) on SiO_2. ^1H NMR (700 MHz, $CDCl_3$) δ 7.18-7.31 (m, 10H), 4.67 (d, *J* = 11.2 Hz, 1H), 4.64 (d, *J* = 11.2 Hz, 1H), **4.45 (d, *J* = 7.7 Hz, 1H)**, 4.13 (dt, *J* = 9.1, 7.0 Hz, 1H), 3.84 (dd, *J* = 11.9, 2.1 Hz, 1H), 3.74-3.81 (m, 2H), 3.67-3.74 (m, 2H), 3.47 (ddd, *J* = 9.8, 4.2, 3.5 Hz, 1H), 3.39 (dd, *J* = 9.8, 7.7 Hz, 1H), 3.28 (s, 3H), 3.26 (s, 3H), 2.95 (t, *J* = 7.0 Hz, 2H), 1.90 (s, 1H), 1.34 (s, 3H), 1.29 (s, 3H); ^{13}C NMR (175 MHz, $CDCl_3$) δ 138.8, 138.5, 128.9, 128.4, 128.1, 127.6, 127.4, 126.3, 103.9, 99.6, 99.5, 79.0, 74.6, 73.6, 72.1, 71.0, 66.05, 61.4, 47.93, 47.87, 36.2, 17.8, 17.6; IR (film, cm^{-1}) 3494, 2925, 2247, 1497, 1454, 1368, 1329, 1222, 1202, 1132, 1109, 907, 729, 697; HRMS (ES) *m/z* calcd for $C_{27}H_{36}O_8$ $[M+NH_4]^+$ 506.2748, found 506.2759.

Sugar Silane 8a and Cyclohexanol Silyl-Linked (11c)

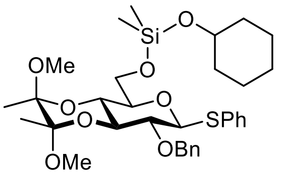

Following general procedure C, **8a** (59 mg, 0.11 mmol), cyclohexanol (10.6 µL, 0.10 mmol), CuCl (0.5 mg, 0.005 mmol), IPr•HCl (4.1 mg, 0.010 mmol), NaOtBu (1 mg, 0.01 mmol), and 4Å molecular sieves (40 mg) were stirred overnight. The product (51 mg, 0.077 mmol, 78%) was obtained as a colorless oil upon flash chromatography (5 to 10% EtOAc/hex) on SiO_2. ^{1}H NMR (500 MHz, $CDCl_3$) δ 7.57-7.61 (m, 2H), 7.43-7.47 (m, 2H), 7.33-7.38 (m, 2H), 7.25-7.32 (m, 4H), 4.82 (d, *J* = 11.0 Hz, 1H), 4.73 (d, *J* = 10.5 Hz, 1H), 4.65 (d, *J* = 9.5 Hz, 1H), 3.99 (dd, *J* = 11.5, 2.0 Hz, 1H), 3.85-3.93 (m, 2H), 3.71-3.91 (m, 2H), 3.46-3.54 (m, 2H), 3.31 (s, 3H), 3.29 (s, 3H), 1.80-1.90 (m, 2H), 1.67-1.76 (m, 2H), 1.50 (dt, *J* = 12.5, 4.0 Hz, 1H), 1.10-1.40 (m, 5H), 1.37 (s, 3H), 1.31 (s, 3H), 0.17 (s, 3H), 0.16 (s, 3H); ^{13}C NMR (125 MHz, $CDCl_3$) δ 138.4, 133.5, 132.4, 128.8, 128.3, 128.2, 127.7, 127.5, 99.7, 99.5, 87.2, 78.2, 77.3, 75.4, 74.9, 70.9, 65.2, 60.7, 48.0, 47.9, 35.9, 35.8, 25.5, 24.4, 17.9, 17.7, -2.3, -2.6; IR (film, cm^{-1}) 3063, 2992, 2931, 2857, 2246, 1703, 1585, 1498, 1454, 1376, 1331, 1256, 1219, 1134; HRMS (ES) *m/z* calcd for $C_{33}H_{48}O_8SSi$ $[M+NH_4]^+$ 650.3177, found 650.3185.

Sugar Silane 8a and Cyclohexanol Glycoside (12c)

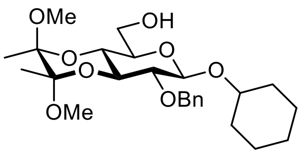

Following general procedure D, **11c** (49 mg, 0.077 mmol), NIS (23 mg, 1.0 mmol), 2,6-DTBMP (32 mg, 0.16 mmol), and TMSOTf (17 µL, 0.093 mmol) were stirred at 0 °C for 10 min, warmed to 0 °C for 1 h, and quenched with TBAF. The product (40 mg, 0.086 mmol, 92%) was obtained as a white solid upon flash chromatography (15 to 20% EtOAc/hex) on SiO_2. ^{1}H NMR (500 MHz, $CDCl_3$) δ 7.40 (d, *J* = 7.0 Hz, 2H), 7.33 (t, *J* = 7.0 Hz, 2H), 7.25-7.31 (m, 1H), 4.87 (d, *J* = 11.0 Hz), 4.78 (d, *J* = 11.5 Hz, 1H), **4.55 (d, *J* = 7.5 Hz, 1H)**, 3.87 (d, *J* = 12.0 Hz, 1H), 3.81 (t, *J* = 10.0 Hz, 1H), 3.62-3.78 (m, 3H), 3.50 (ddd, *J* = 9.5, 5.0, 3.0 Hz, 1H), 3.41 (dd, *J* = 10.0, 7.5 Hz, 1H), 3.31 (s, 3H), 3.27 (s, 3H), 1.87-2.06 (m, 3H), 1.71-1.84 (m, 2H), 1.51-1.60 (m, 1H), 1.16-1.51 (m, 4 H), 1.36 (s, 3H), 1.31 (s, 3H); ^{13}C NMR (125 MHz, $CDCl_3$) δ 138.8, 128.2, 127.8, 127.5, 102.3, 99.6, 99.5, 79.1, 78.4, 74.8, 73.6, 72.3, 66.3, 61.5, 47.9, 33.8, 32.0, 25.6, 24.1, 24.0, 18.9, 17.8, 17.6; IR (film, cm^{-1}) 3495, 2932, 2353, 1721, 1454, 1374, 1125, 1041; HRMS (ES) *m/z* calcd for $C_{25}H_{38}O_8$ $[M+NH_4]^+$ 484.2905, found 484.2900.

Sugar Silane 8a and Methyl 2,3,4-*O*-Tribenzyl-Glucopyranose Silyl-Linked (11d)

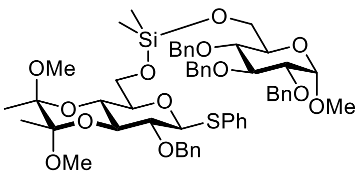

Following general procedure B, **8a** (48 mg, 0.090 mmol) was slowly added over 1 h to methyl 2,3,4-*O*-tribenzyl-α-D-glucopyranoside (28 mg, 0.060 mmol) and $B(C_6F_5)_3$ and stirred overnight. The product (36 mg, 0.036 mmol, 60%) was obtained as a white solid upon flash chromatography (10 to 18% EtOAc/hex) on SiO_2. ^{1}H NMR (700 Mz, $CDCl_3$) δ 7.52-7.55 (m, 2H), 7.40-7.43 (m, 2H), 7.19-7.36 (m, 21H), 4.96 (d, *J* = 11.2 Hz, 1H), 4.85 (d, *J* = 10.5 Hz, 1H), 4.75-4.81 (m, 3H), 4.70 (d, *J* = 10.5 Hz, 1H), 4.65 (d, *J* = 11.9 Hz, 1H), 4.58-4.64 (m, 3H), 3.98 (t, *J* = 9.8 Hz, 1H), 3.94 (dd, *J* = 11.2, 1.4 Hz, 1H), 3.81-3.92 (m, 4H), 3.72 (t, *J* = 9.8 Hz, 1H), 3.62 (dt, *J* = 10.0, 3.0 Hz, 1H), 3.55 (t, *J* = 9.8 Hz, 1H), 3.50 (dd, *J* = 9.8, 4.2 Hz, 1H), 3.41-3.46 (m, 2H), 3.33 (s, 3H), 3.25 (s, 3H), 3.22 (s, 3H), 1.33 (s, 3H), 1.26 (s, 3H), 0.14 (s, 3H), 0.12 (s, 3H); ^{13}C NMR (175 MHz, $CDCl_3$) 138.8, 138.44, 138.38, 138.2, 133.4, 132.3, 128.8, 128.42, 128.39, 128.37, 128.3, 128.1, 128.05, 128.01, 127.8, 127.72, 127.68, 127.62, 127.58, 127.5, 99.6, 99.5, 98.0, 87.3, 82.1, 80.0, 78.1, 77.42, 77.39, 75.8, 75.4, 74.85, 74.81, 73.3, 71.1, 65.1, 61.5, 60.9, 55.0, 48.0, 47.9, 29.7, 17.8, 17.6, -2.9, -3.0; IR (film, cm^{-1}) 2929, 2361, 1585, 1497, 1455, 1366, 1257, 1134, 1048; HRMS (ES) *m/z* calcd for $C_{55}H_{68}O_{13}SSi$ $[M+Na]^+$ 1019.4042, found 1019.4042.

Sugar Silane 8a and Methyl 2,3,4-*O*-Tribenzyl-Glucopyranose Glycoside (12d)

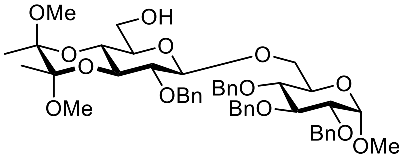

Following general procedure D, **11d** (32 mg, 0.032 mmol), NIS (9.4 mg, 0.042 mmol), 2,6-DTBMP (13 mg, 0.064 mmol), and TMSOTf (7 μL, 0.04 mmol) were stirred at -40 °C for 10 min, warmed to 0 °C for 3 h, and quenched with TBAF. The product was obtained as a white solid (20 mg, 0.024 mmol, 74%) upon flash chromatography (40 to 55% EtOAc/hex) on SiO_2. ^{1}H NMR (700 MHz, $CDCl_3$) δ 7.34-7.37 (m, 2H), 7.22-7.34 (m, 13H), 7.11-7.19 (m, 5H), 4.96 (d, *J* = 10.5 Hz, 1H), 4.75-4.84 (m, 4H), 4.66 (d, *J* = 11.2 Hz, 2H), 4.61 (d, *J* = 3.5 Hz, 1H), 4.49 (d, *J* = 11.2 Hz, 1H), **4.36 (d, *J* = 7.7 Hz, 1H)**, 4.06 (dd, *J* = 11.2, 1.4 Hz, 1H), 3.96 (t, *J* = 9.8 Hz, 1H), 3.82 (dd, *J* = 11.9, 2.1 Hz, 1H), 3.79 (t, *J* = 9.8 Hz, 1H), 3.76 (dd, *J* = 10.5, 2.1 Hz, 1H), 3.65-3.73 (m, 3H), 3.55 (t, *J* = 9.8 Hz, 1H), 3.52 (dd, *J* = 9.8, 3.5 Hz, 1H), 3.42-3.48 (m, 2H), 3.33 (s, 3H), 3.28 (s, 3H), 3.24 (s, 3H), 1.32 (s, 3H), 1.28 (s, 3H); ^{13}C NMR (175 MHz, $CDCl_3$) δ 138.8, 138.5, 138.4, 138.2, 128.4, 128.31, 128.29, 128.2, 128.1, 128.0, 127.9, 127.7, 127.62, 127.55, 127.5, 127.4, 104.0, 99.6, 99.5, 98.2, 82.0, 79.7, 78.6, 77.6, 75.7, 74.83, 74.79, 73.7, 73.4, 72.7, 69.8, 68.8, 65.9, 61.3, 55.2, 47.91, 47.90, 17.8, 17.6; IR (film, cm^{-1}) 3498, 3030, 2923, 1720, 1496, 1454, 1367, 1194, 1134, 1093, 1028, 737, 697; HRMS (ES) *m/z* calcd for $C_{47}H_{58}O_{13}$ $[M+Na]^+$ 853.3770, found 853.3750.

Sugar Silane 8a and 1,2:3,4-Diisopropylidene Galactopyranose Glycoside (12e)

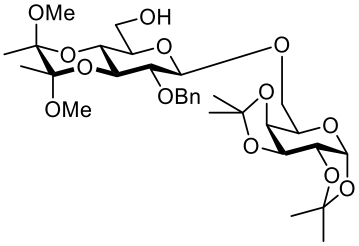

Following general procedure C, **8a** (59 mg, 0.11 mmol), 1,2:3,4-di-*O*-isopropylidene-galactopyranose (26 mg, 0.10 mmol), CuCl•IPr (2.4 mg, 0.0050 mmol), NaOtBu (0.48 mg, 0.0050 mmol), and 4Å molecular sieves (40 mg) were stirred overnight. A mixture of the desired silyl-linked intermediate (39 mg, 0.049 mmol, 49%) and the silyl-linked dimer were obtained upon flash chromatography (5 to 15% EtOAc/hex). The mixture was immediately subjected to general procedure D, taking care that all present thiophenyl leaving groups were activated, whereupon NIS (43 mg, 0.19 mmol), 2,6-DTBMP (51 mg, 0.25 mmol), and TMSOTf (32 μL, 0.18 mmol) were stirred at -40 °C for 10 min, warmed to 0 °C for 3 h, and quenched with TBAF. The desired product (26 mg, 0.041 mmol, 41% overall yield) was obtained as a white solid upon flash chromatography (40 to 50% EtOAc/hex) on SiO_2. ^{1}H NMR (700 MHz, $CDCl_3$) δ 7.41 (d, *J* = 7.7 Hz, 2H), 7.30 (t, *J* = 7.0 Hz, 2H), 7.22-7.26 (m, 1H), 5.54 (d, *J* = 4.9 Hz, 1H), 4.92 (d, *J* = 11.2 Hz, 1H), 4.74 (d, *J* = 11.9 Hz, 1H), 4.57 (d, *J* = 7.7 Hz, 1H), **4.48 (d, *J* = 7.7 Hz, 1H)**, 4.31 (dd, *J* = 2.8, 2.1 Hz, 1H), 4.25 (d, *J* = 9.1 Hz, 1H), 4.05 (dd, *J* = 10.5, 4.9 Hz, 1H), 4.01 (t, *J* = 5.6 Hz, 1H), 3.82-3.89 (m, 1H), 3.80 (t, *J* = 9.8 Hz, 1H), 3.75 (dd, *J* = 10.5, 7.0 Hz, 1H), 3.63-3.71 (m, 2H), 3.46-3.52 (m, 1H), 3.40 (dd, *J* = 9.8, 7.0 Hz, 1H), 3.29 (s, 3H), 3.24 (s, 3H), 1.52 (s, 3H), 1.43 (s, 3H), 1.34 (s, 3H), 1.322 (s, 3H), 1.316 (s, 3H), 1.28 (s, 3H); ^{13}C (175 MHz, $CDCl_3$) 139.1, 128.1, 127.7, 127.3, 104.4, 99.6, 99.5, 96.4, 78.7, 74.3, 73.7, 72.0, 71.1, 70.7, 70.5, 69.4, 66.8, 66.3, 61.5, 47.89, 47.86, 26.0, 26.0, 25.0, 24.4, 17.8, 17.6; IR (film, cm^{-1}) 3494, 2989, 2933, 1701, 1454, 1376, 1254, 1210, 1133, 1113, 1069, 1045, 1005; HRMS (ES) *m/z* calcd for $C_{31}H_{46}O_{13}$ $[M+K]^+$ 665.2570, found 665.2575.

Sugar Silane 8a and Ketosteroid Silyl-Linked (11f)

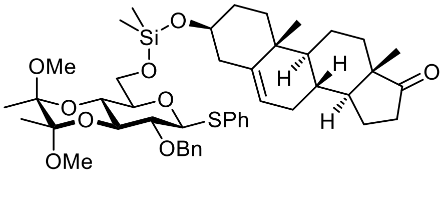

Following general procedure B, **8a** (80 mg, 0.15 mmol) was slowly added over 1 h to a solution of steroid (29 mg, 0.10 mmol) and $B(C_6F_5)_3$ in 1:1 PhMe:CH_2Cl_2 and stirred overnight. The product (65 mg, 0.079 mmol, 79%) was obtained as a white solid upon flash chromatography (10 to 16% EtOAc/hex) on SiO_2. ^{1}H NMR (700 MHz, $CDCl_3$) δ 7.52-7.55 (m, 2H), 7.40-7.42 (m, 2H), 7.31-7.34 (m, 2H), 7.21-7.29 (m, 4H), 5.33 (m, 1H), 4.79 (d, *J* = 10.5 Hz, 1H), 4.70 (d, *J* = 10.5 Hz, 1H), 4.62 (d, *J* = 9.1 Hz, 1H), 3.96 (dd, *J* = 11.2, 1.4 Hz, 1H), 3.83-3.89 (m, 2H), 3.76 (t, *J* = 9.8 Hz, 1H), 3.65 (ddd, *J* = 15.4, 11.2, 4.9 Hz, 1H), 3.44-3.49 (m, 2H), 3.28 (s, 3H), 3.26 (s, 3H), 2.44 (dd, *J* = 18.9, 8.4 Hz, 1H), 2.29 (td, *J* = 11.2, 2.1 Hz, 1H), 2.23 (ddd, *J* = 13.3, 4.9,

2.1 Hz), 2.02-2.10 (m, 2H), 1.91 (ddd, J = 14.7, 8.4, 6.3 Hz, 1H), 1.73-1.84 (m, 3H), 1.39-1.66 (m, 6H), 1.34 (s, 3H), 1.29 (s, 3H), 1.19-1.30 (m, 3H), 1.02 (td, J = 13.6, 3.5 Hz, 1H), 0.99 (s, 3H), 0.91 (td, J = 11.6, 4.2 Hz, 1H), 0.86 (s, 3H), 0.15 (s, 3H), 0.14 (s, 3H); ^{13}C NMR (175 MHz, $CDCl_3$) 221.2, 141.4, 138.4, 133.6, 132.1, 128.8, 128.3, 128.1, 127.7, 127.4, 120.7, 99.6, 99.5, 87.2, 78.1, 77.4, 75.4, 74.8, 71.9, 65.0, 60.6, 51.7, 50.2, 48.0, 47.9, 47.5, 42.4, 37.2, 36.6, 35.8, 31.7, 31.44, 31.42, 30.8, 21.9, 20.3, 19.4, 17.8, 17.7, 13.5, -2.4, -2.5; IR (film, cm^{-1}) 2936, 2158, 1738, 1454, 1375, 1256, 1132, 1110, 1048, 1033, 899, 849, 797, 735, 697; HRMS (ES) *m/z* calcd for $H_{46}H_{64}O_9SSi$ $[M+NH_4]^+$ 838.4379, found 838.4365.

Sugar Silane 8a and Ketosteroid Glycoside (12f)

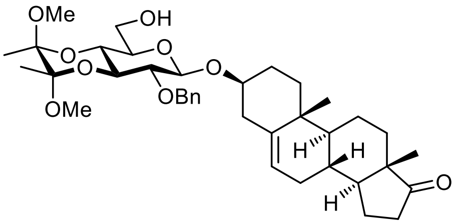

Following general procedure D, **11f** (65 mg, 0.079 mmol), NIS (23 mg, 0.10 mmol), 2,6-DTBMP (33 mg, 0.16 mmol), and TMSOTf (17 μL, 0.095 mmol) were stirred at -40 °C for 10 min, warmed to 0 °C for 1 h, and quenched with TBAF. The product was obtained as a white solid (35 mg, 0.053 mmol, 67%) upon flash chromatography (20 to 40% EtOAc/hex) on SiO_2. ^{1}H NMR (700 MHz, $CDCl_3$) δ 7.39 (d, J = 7.0 Hz, 2H), 7.32 (t, J = 7.7 Hz, 2H), 7.24-7.28 (m, 1H), 5.35 (d, J = 4.9 Hz, 1H), 4.84 (d, J = 11.9 Hz, 1H), 4.78 (d, J = 11.2 Hz, 1H), **4.52 (d, J = 7.7 Hz, 1H)**, 3.84 (d, J = 11.2 Hz, 1H), 3.79 (t, J = 9.8 Hz, 1H), 3.64-3.74 (m, 2H), 3.53 (ddd, J = 15.4, 11.2, 4.2 Hz, 1H), 3.45-3.50 (m, 1H), 3.39 (dd, J = 9.8, 7.0 Hz, 1H), 3.29 (s, 3H), 3.25 (s, 3H), 2.45 (dd, J = 19.6, 8.4 Hz, 1H), 2.40 (d, J = 11.2 Hz, 1H), 2.30 (t, J = 12.6 Hz, 1H), 2.10-2.24 (m, 2H), 1.81-1.97 (m, 5H), 1.59-1.70 (m, 4H), 1.40-1.58 (m, 3H), 1.34 (s, 3H), 1.29 (s, 3H), 1.22-1.32 (m, 3H), 1.01-1.10 (m, 1H), 1.00 (s, 3H), 0.95-1.01 (m, 1H); ^{13}C NMR (175 MHz, $CDCl_3$) δ 221.1, 140.6, 138.8, 128.2, 127.8, 127.5, 121.3, 102.5, 99.55, 99.49, 79.9, 79.2, 74.9, 73.6, 72.3, 66.2, 61.5, 51.7, 50.2, 47.92, 47.90, 47.5, 39.0, 37.2, 36.8, 35.8, 31.5, 31.4, 30.8, 29.8, 21.9, 20.3, 19.4, 17.8, 17.6, 13.5; IR (film, cm^{-1}) 3480, 2918, 1739, 1452, 1375, 1136, 1103, 1046, 882; HRMS (ES) *m/z* calcd for $C_{38}H_{54}O_9$ $[M+NH_4]^+$ 672.4093, found 672.4106.

Sugar Silane 8b and Butanol Silyl-Linked (11g)

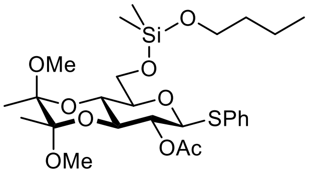

Following general procedure C, **8b** (54 mg, 0.11 mmol), butanol (9.2 μL, 0.10 mmol), CuCl (0.5 mg, 0.005 mmol), IPr•HCl (4.3 mg, 0.010 mmol), NaOtBu (1 mg, 0.010 mmol), and 4Å molecular sieves (40 mg) were stirred overnight. The product (48 mg, 0.086 mmol, 86%) was obtained as a colorless oil upon flash chromatography (10 to 15% EtOAc/hex) on SiO_2. ^{1}H NMR

(700 MHz, $CDCl_3$) δ 7.47-7.51 (m, 2H), 7.22-7.28 (m, 3H), 4.96 (t, *J* = 9.8 Hz, 1H), 4.64 (d, *J* = 9.8 Hz, 1H), 3.96 (dd, *J* = 11.2, 1.4 Hz, 1H), 3.79-3.85 (m, 2H), 3.75 (t, *J* = 9.8 Hz, 1H), 3.66 (t, *J* = 7.0 Hz, 1H), 3.51 (ddd, *J* = 9.8, 4.9, 1.4 Hz, 1H), 3.23 (s, 3H), 3.22 (s, 3H), 2.01 (s, 3H), 1.50 (quin, *J* = 7.0 Hz, 2H), (sex, *J* = 7.0 Hz, 2H), 1.26 (s, 3H), 1.25 (s, 3H), 0.88 (t, *J* = 7.7 Hz, 3H), 0.12 (s, 3H), 0.11 (s, 3H); ^{13}C NMR (175 MHz, $CDCl_3$) δ 169.2, 133.1, 132.2, 128.8, 127.6, 99.8, 99.5, 86.2, 78.5, 71.9, 69.3, 65.2, 62.3, 60.7, 47.9, 47.6, 34.7, 20.9, 18.9, 17.61, 17.57, 13.9, -3.0, -3.2; IR (film, cm^{-1}) 2955, 2932, 1754, 1368, 1255, 1223, 1128, 1071, 1034, 849; HRMS (ES) *m/z* calcd for $C_{26}H_{42}O_9SSi$ $[M+NH_4]^+$ 576.2657, found 576.2649.

Sugar Silane 8b and Butanol Glycoside (12g)

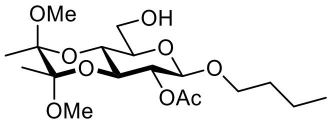

Following general procedure D, **11g** (46 mg, 0.082 mmol), NIS (24 mg, 0.11 mmol), 2,6-DTBMP (34 mg, 0.16 mmol), and TMSOTf (18 μL, 0.010 mmol) were stirred at -40 °C for 10 min, warmed to 0 °C for 3 h, and quenched with TBAF. The product (24 mg, 0.061 mmol, 75%) was obtained as a white solid upon flash chromatography (20 to 30% EtOAc/hex) on SiO_2. ^{1}H NMR (500 MHz, $CDCl_3$) δ 4.95 (dd, *J* = 10.0, 8.0 Hz, 1H), **4.44 (d, *J* = 8.0 Hz, 1H)**, 3.71-3.91 (m, 5H), 3.52 (ddd, *J* = 9.0, 4.5, 3.0 Hz, 1H), 3.46 (dt, *J* = 9.5, 7.0 Hz, 1H), 3.25 (s, 3H), 3.24 (s, 3H), 2.08 (s, 3H), 2.00 (s, 1H), 1.46-1.62 (m, 2H), 1.24-1.42 (m, 2H), 1.29 (s, 3H), 1.28 (s, 3H), 0.90 (t, *J* = 7.5 Hz, 3H); ^{13}C NMR (100 MHz, $CDCl_3$) δ 169.2, 101.8, 99.64, 99.55, 73.9, 70.7, 70.1, 69.8, 65.9, 61.2, 47.9, 47.6, 31.4, 20.8, 18.9, 17.6, 17.5, 13.7; IR (film, cm^{-1}) 3492, 2956, 1748, 1457, 1370, 1228, 1127, 906, 730; HRMS (ES) *m/z* calcd for $C_{18}H_{32}O_9$ $[M+NH_4]^+$ 410.2385, found 410.2384.

Sugar Silane 9 and Butanol Silyl-Linked (11h)

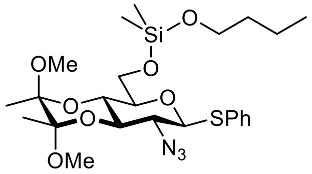

Following general procedure C, **9** (52 mg, 0.11 mmol), butanol (9.2 μL, 0.10 mmol), CuCl•IPr (2.5 mg, 0.0050 mmol), NaOtBu (0.48 mg, 0.0050 mmol), and 4Å molecular sieves (40 mg) were stirred overnight. The product (46 mg, 0.085 mmol, 85%) was obtained as a white solid upon flash chromatography (5 to 15% EtOAc/hex) on SiO_2. ^{1}H NMR (700 MHz, $CDCl_3$) δ 7.55-7.60 (m, 2H), 7.26-7.32 (m, 3H), 4.39 (d, *J* = 9.8 Hz, 1H), 3.94 (dd, *J* = 11.9, 1.4 Hz, 1H), 3.84 (dd, *J* = 11.2, 4.2 Hz, 1H), 3.68-3.74 (m, 2H), 3.67 (t, *J* = 7.0 Hz, 2H), 3.44-3.48 (m, 1H), 3.92 (t, *J* = 9.8 Hz, 1H), 3.31 (s, 3H), 3.24 (s, 3H), 1.51 (quin, *J* = 7.0 Hz, 2H), 1.29-1.36 (m, 2H), 1.31 (s, 3H), 1.26 (s, 3H), 0.88 (t, *J* = 7.7 Hz, 3H), 0.14 (s, 3H), 0.12 (s, 3H); ^{13}C NMR (175 MHz, $CDCl_3$) δ 133.6, 131.3, 128.9, 128.3, 100.2, 99.7, 86.1, 78.5, 73.2, 65.3, 62.0, 61.4, 60.5, 48.0, 48.0, 34.7, 18.9, 17.6, 17.5, 13.8, -3.0, -3.2; IR (film, cm^{-1}) 2956, 2930, 2109, 1454, 1439,

1375, 1367, 1288, 1256, 1111, 1092, 1067, 1030, 848; HRMS (ES) *m/z* calcd for $C_{24}H_{39}N_3O_7SSi$ $[M+NH_4]^+$ 559.2616, found 559.2620.

Sugar Silane 9 and Butanol Glycoside (12h)

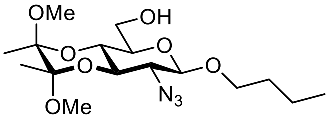

Following general procedure D, **11h** (44 mg, 0.081 mmol), NIS (24 mg, 0.11 mmol), 2,6-DTBMP (33 mg, 0.16 mmol), and TMSOTf (18 µL, 0.097 mmol) were stirred at -40 °C for 10 min, warmed to 0 °C for 1 h, and quenched with TBAF. The product (24 mg, 0.064 mmol, 80%) was obtained as a white solid upon flash chromatography (25 to 35% EtOAc/hex) on SiO_2. ^1H (700 MHz, $CDCl_3$) δ **4.26 (d, *J* = 8.4 Hz, 1H)**, 3.84 (dt, *J* = 9.8, 6.3 Hz, 1H), 3.79 (d, *J* = 11.9 Hz, 1H), 3.63-3.70 (m, 2H), 3.52 (t, *J* = 9.8 Hz, 1H), 3.48 (dt, *J* = 9.1, 7.0 Hz, 1H), 3.39-3.42 (m, 1H), 3.35 (dd, *J* = 10.5, 7.7 Hz, 1H), 3.24 (s, 3H), 3.19 (s, 3H), 1.84 (s, 1H), 1.51-1.61 (m, 2H), 1.29-1.40 (m, 2H), 1.28 (s, 3H), 1.22 (s, 3H), 0.86 (t, *J* = 7.0 Hz, 3H); ^{13}C NMR (175 MHz, $CDCl_3$) δ 102.7, 100.0, 99.7, 73.9, 70.6, 70.4, 66.0, 62.8, 61.1, 48.0, 48.0, 31.5, 19.1, 17.6, 17.5, 13.8; IR (film, cm^{-1}) 3501, 2956, 2874, 2108, 1460, 1371, 1279, 1262, 1222, 1202, 1117, 1099, 1065, 1029; HRMS (ES) *m/z* calcd for $C_{16}H_{29}N_3O_7$ $[M+Na]^+$ 398.1898, found 398.1899.

Sugar Silane 10 and Butanol Silyl-Linked (11i)

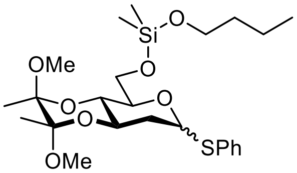

Following general procedure C, **10** (47 mg, 0.11 mmol), butanol (9.2 µL, 0.10 mmol), CuCl•IPr (2.5 mg, 0.0050 mmol), NaOtBu (0.48 mg, 0.0050 mmol), and 4Å molecular sieves (40 mg) were stirred overnight. The product (44 mg, 0.088 mmol, 88%) was obtained as a white solid upon flash chromatography (5 to 15% EtOAc/hex) on SiO_2. ^1H NMR (700 MHz, $CDCl_3$) δ 7.49 (d, *J* = 7.0 Hz, 1.1H, *β*-anomer), 7.45 (d, *J* = 7.7 Hz, 2H, *α*-anomer), 7.19-7.28 (m, 4.6H), 5.65 (d, *J* = 4.9 Hz, 1H, *α*-anomer), 4.82 (dd, *J* = 11.2, 1.4 Hz, 0.54H, *β*-anomer), 4.23 (dt, *J* = 9.8, 2.1 Hz, 1H, *α*-anomer), 4.12 (dt, *J* = 10.5, 5.6 Hz, 1H, *α*-anomer), 3.96 (d, *J* = 4.9 Hz, 0.54H, *β*-anomer), 3.80-3.92 (m, 3.2H), 3.64-3.70 (m, 2H), 3.62 (*t*, *J* = 7.0 Hz, 1H, *α*-anomer), 3.60 (t, *J* = 9.1 Hz, 0.54H, *β*-anomer), 3.45 (ddd, *J* = 9.8, 4.2, 1.4 Hz, 0.54H, *α*-anomer), 3.31 (s, 3H), 3.27 (s, 3H), 3.25 (s, 3H), 2.15-2.23 (m, 2H, *α*-anomer), 2.13 (ddd, *J* = 12.6, 4.2, 1.4 Hz, 0.54H, *β*-anomer), 1.84 (q, *J* = 11.9 Hz, 0.54H, *β*-anomer), 1.46-1.53 (m, 3.1H), 1.28-1.35 (m, 3.1H), 1.31 (s, 3H, *α*-anomer), 1.30 (s, 3H, *α*-anomer), 1.28 (s, 1.6H, *β*-anomer), 1.27 (s, 1.6H, *β*-anomer), 0.88 (t, *J* = 7.0 Hz, 4.6H), 0.123 (s, 1.6H, *β*-anomer), 0.118 (s, 1.6H, *β*-anomer), 0.08 (s, 6H, *α*-anomer); ^{13}C NMR (175 MHz, $CDCl_3$) δ 135.1, 133.9, 131.7, 131.4, 128.8, 128.7, 127.3, 127.0, 99.9, 99.84, 99.82, 84.3, 82.4, 78.6, 71.4, 68.7, 68.3, 67.4, 65.8, 62.3, 61.1, 60.7, 48.0, 48.0, 47.92, 47.89, 36.0, 35.5, 34.7, 34.6, 18.9, 17.81, 17.76, 17.7, 13.8, -3.0, -3.10, -3.14, -3.3; IR (film, cm^{-1}) 2956, 1584, 1456, 1376, 1255, 1114, 1074, 1051, 1037, 976, 926, 882, 848, 797, 740, 691, 642; HRMS (ES) *m/z* calcd for $C_{24}H_{40}O_7SSi$ $[M+NH_4]^+$ 518.2602, 518.2602.

Sugar Silane 10 and Butanol Glycoside (12i)

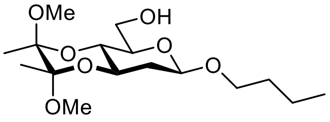

Following general procedure D, **11i** (32 mg, 0.064 mmol), NIS (19 mg, 0.083 mmol), 2,6-DTBMP (26 mg, 0.13 mmol), and TMSOTf (14 µL, 0.077 mmol) were stirred at -78 °C for 1 h and quenched with TBAF. The product (16 mg, 0.048 mmol, 90%) was obtained as a white solid upon flash chromatography (25 to 35% EtOAc/hex) on SiO_2. ^{1}H (700 MHz, $CDCl_3$) δ **4.57 (dd, *J* = 9.8, 1.4 Hz, 1H)**, 3.82-3.88 (m, 2H), 3.78 (ddd, *J* = 12.6, 9.8, 4.2 Hz, 1H), 3.71-3.76 (m, 1H), 3.57 (t, *J* = 9.8 Hz, 1H), 3.42-3.48 (m, 2H), 3.26 (s, 3H), 3.24 (s, 3H), 2.04 (ddd, *J* = 12.6, 4.2, 2.1 Hz, 1H), 1.20 (s, 1H), 1.69 (td, *J* = 12.6, 9.8 Hz, 1H), 1.56 (quin, *J* = 7.0 Hz, 2H), 1.35 (sept, *J* = 7.0 Hz, 2H), 1.29 (s, 3H), 1.28 (s, 3H), 0.90 (t, *J* = 7.0 Hz, 3H); ^{13}C (175 MHz, $CDCl_3$) 100.7, 99.80, 99.78, 73.9, 69.5, 68.3, 67.6, 61.6, 48.0, 47.9, 35.9, 31.6, 19.2, 17.8, 17.7, 13.8; IR (film, cm^{-1}) 3472, 2957, 1456, 1373, 1118, 1092, 1050, 1035, 926, 885, 846; HRMS (ES) *m/z* calcd for $C_{16}H_{30}O_7$ $[M+Na]^+$ 357.1884, found 357.1882.

1,6-Anhydro Glucopyranoside (18)

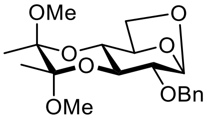

The 1,6-anhydro byproduct was synthesized for spectral comparison. Following the general procedure, **7a** (45 mg, 0.094 mmol), NIS (28 mg, 0.12 mmol), 2,6-DTBMP (58 mg, 0.28 mmol), and TMSOTf (38 µL, 0.21 mmol) were stirred at -40 °C for 10 min, warmed to 0 °C for 2 h, and quenched with TBAF. The product (18 mg, 0.049 mmol, 51%) was obtained as a white solid upon flash chromatography (5 to 15% EtOAc/hex) on SiO_2. ^{1}H NMR (500 MHz, $CDCl_3$) δ 7.30-7.38 (m, 4H), 7.24-7.30 (m, 1H), **5.35 (s, 1H)**, 4.79 (d, *J* = 12.0 Hz, 1H), 4.70 (d, *J* = 12.0 Hz, 1H), 4.42 (d, *J* = 2.1 Hz, 1H), 3.94 (t, *J* = 12.6 Hz, 1H), 3.62-3.70 (m, 2H), 3.51 (dd, *J* = 9.1, 4.9 Hz, 1H), 3.36 (d, *J* = 11.2 Hz, 1H), 3.31 (s, 3H), 3.24 (s, 3H), 1.33 (s, 3H), 1.32 (s, 3H); ^{13}C NMR (125 MHz, $CDCl_3$) δ 138.2, 128.3, 127.6, 127.5, 103.3, 100.4, 100.1, 80.4, 75.2, 72.6, 70.6, 70.2, 70.1, 47.91, 47.86, 17.9, 17.8; IR (film, cm^{-1}) 2991, 2950, 2902, 1497, 1454, 1375, 1210, 1134, 1112, 1076, 1051, 1036; HRMS (ES) *m/z* calcd for $C_{19}H_{26}O_7$ $[M+NH_4]^+$ 389.1571, 389.1571.

7a Silyl-Linked Dimer (19)

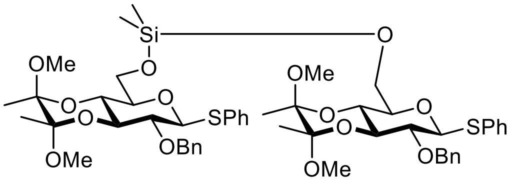

The bis-dimer was synthesized for spectral comparison. Following the general procedure, **8a** (51 mg, 0.095 mmol), **7a** (41 mg, 0.086 mmol), CuCl•IPr (2.4 mg, 0.0050 mmol), NaOtBu (0.48 mg, 0.005 mmol), and 4Å molecular sieves (40 mg) were stirred overnight. The product (63 mg, 0.062 mmol, 72%) was obtained as a white solid upon flash chromatography (5 to 15% EtOAc/hex) on SiO_2. ^{1}H NMR (700 MHz, $CDCl_3$) δ 7.53-7.57 (m, 4H), 7.40-7.44 (m, 4H), 7.33 (t, J = 7.0 Hz, 4H), 7.21-7.31 (m, 8H), 4.80 (d, J = 10.5 Hz, 2H), 4.71 (d, J = 10.5 Hz, 2H), 4.63 (d, J = 9.8 Hz, 2H), 4.00 (dd, J = 11.2, 1.4 Hz, 2H), 3.82-3.90 (m, 4H), 3.74 (t, J = 9.8 Hz, 2H), 3.44-3.49 (m, 4H), 3.26 (s, 6H), 3.24 (s, 6H), 1.33 (s, 6H), 1.27 (s, 6H), 0.16 (s, 6H); ^{13}C NMR (175 MHz, $CDCl_3$) 138.4, 133.5, 132.3, 128.8, 128.3, 128.2, 127.7, 127.4, 99.6, 99.5, 87.3, 78.2, 77.5, 75.4, 74.8, 65.2, 60.8, 48.0, 47.9, 17.8, 17.6, -3.0; HRMS (ES) *m/z* calcd for $C_{52}H_{68}O_{14}S_2Si$ $[M+NH_4]^+$ 1026.4158, found 1026.4155.

References

1) Crich, D.; Jayalath, P. *J. Org. Chem.* **2005**, *70*, 7252.
2) Buchan, Z. A.; Bader, S. J.; Montgomery, J. *Angew. Chem. Int. Ed.* **2009**, *48*, 4840.
3) Rye, C. S.; Withers, S. G. *J. Org. Chem.* **2002**, *67*, 4505.
4) Douglas, N. L.; Ley, S. V.; Osborn, H. M. I.; Owen, Dayfdd, R.; Priepke, H; W. M.; Warriner, S. L. *Synlett* **1996**, *8*, 793.
5) Buskas, T.; Garegg, P. J.; Konradsson, P.; Maloisel, J. L. *Tetrahedron: Asymmetry* **1994**, *5*, 2187.
6) Sakurai, K.; Kahne, D. *Tetrahedron Lett.* **2010**, *51*, 3724.

Phenyl 6-*O*-*tert*-butyldimethylsilyl-3,4-*O*-(2',3'-dimethoxybutane-2',3'-diyl)-thio-β-D-glucopyranoside (13)

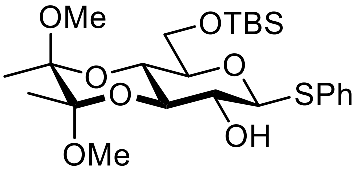

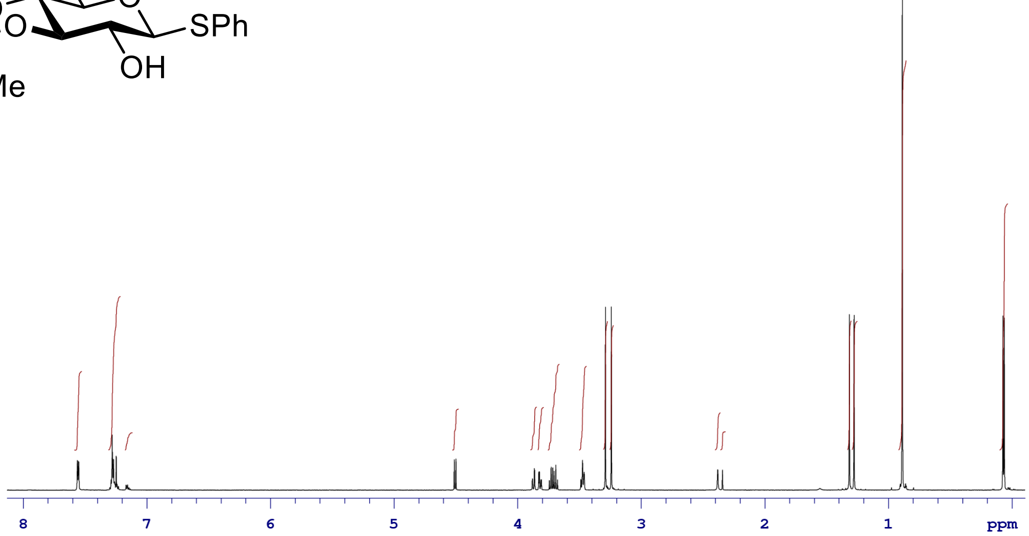

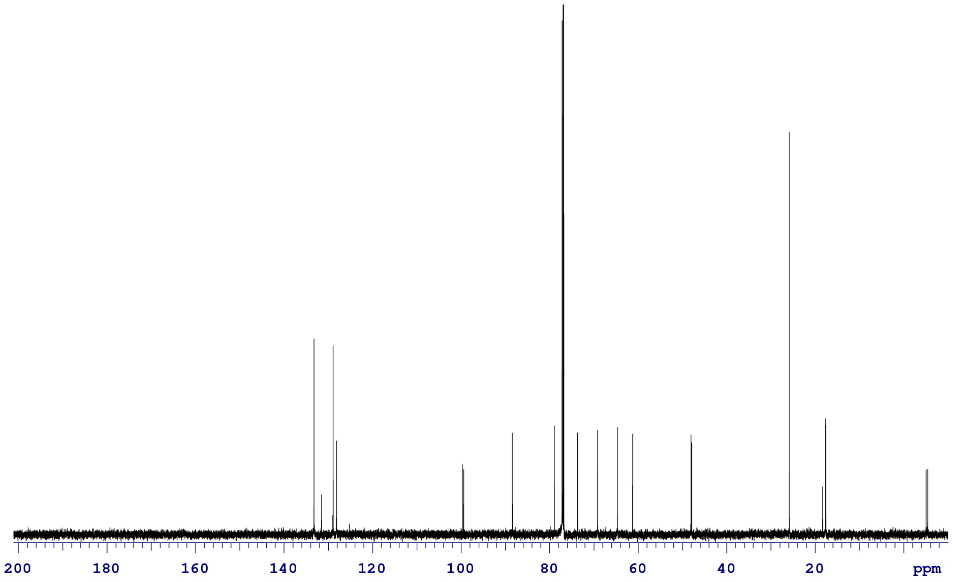

Sugar Silane (3)

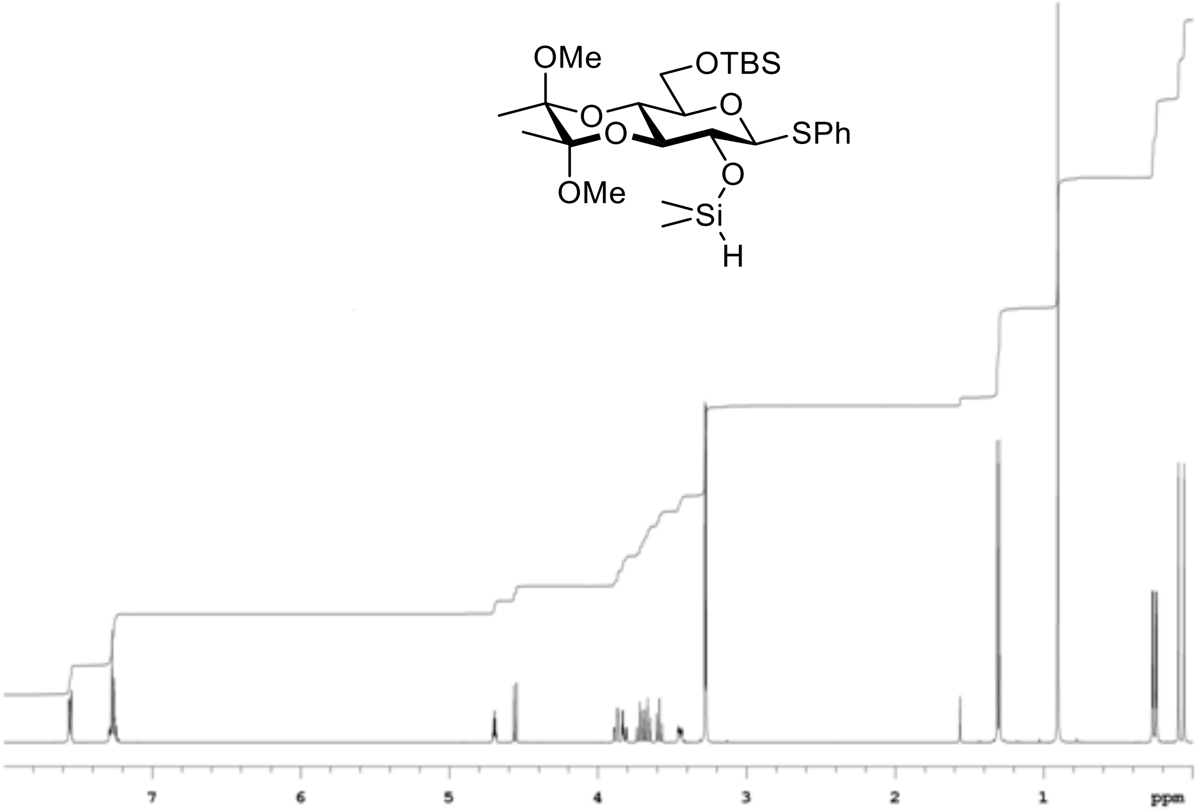

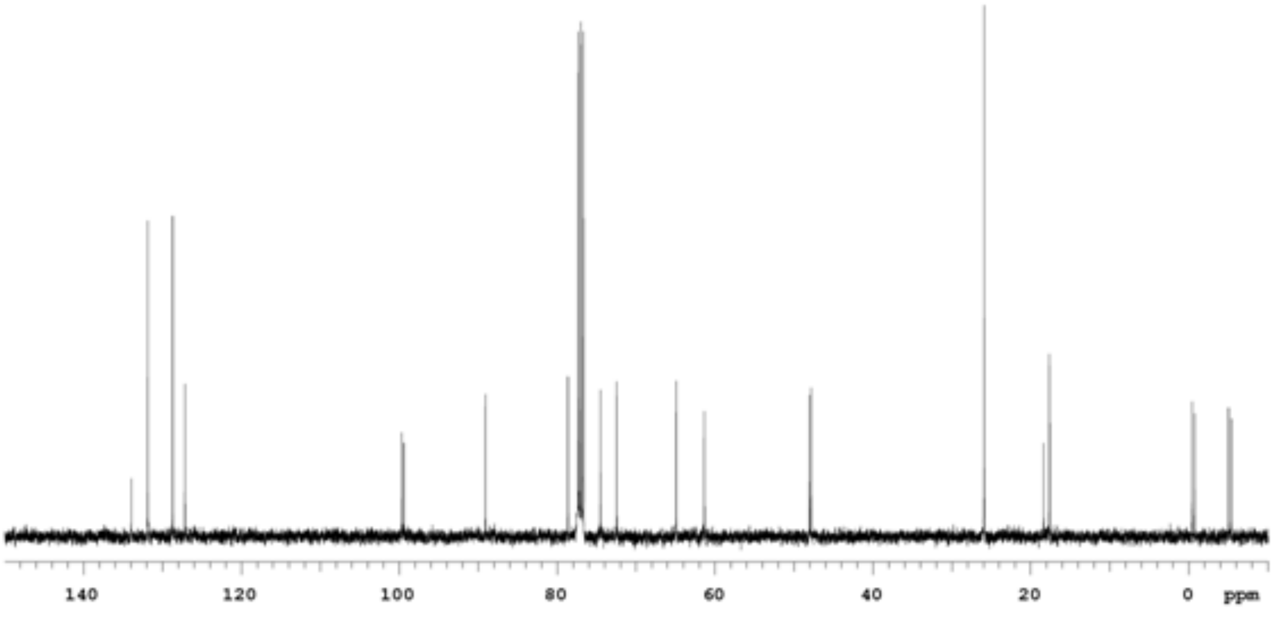

Sugar Silane 1a and (-)-Menthol Silyl-Linked (4a)

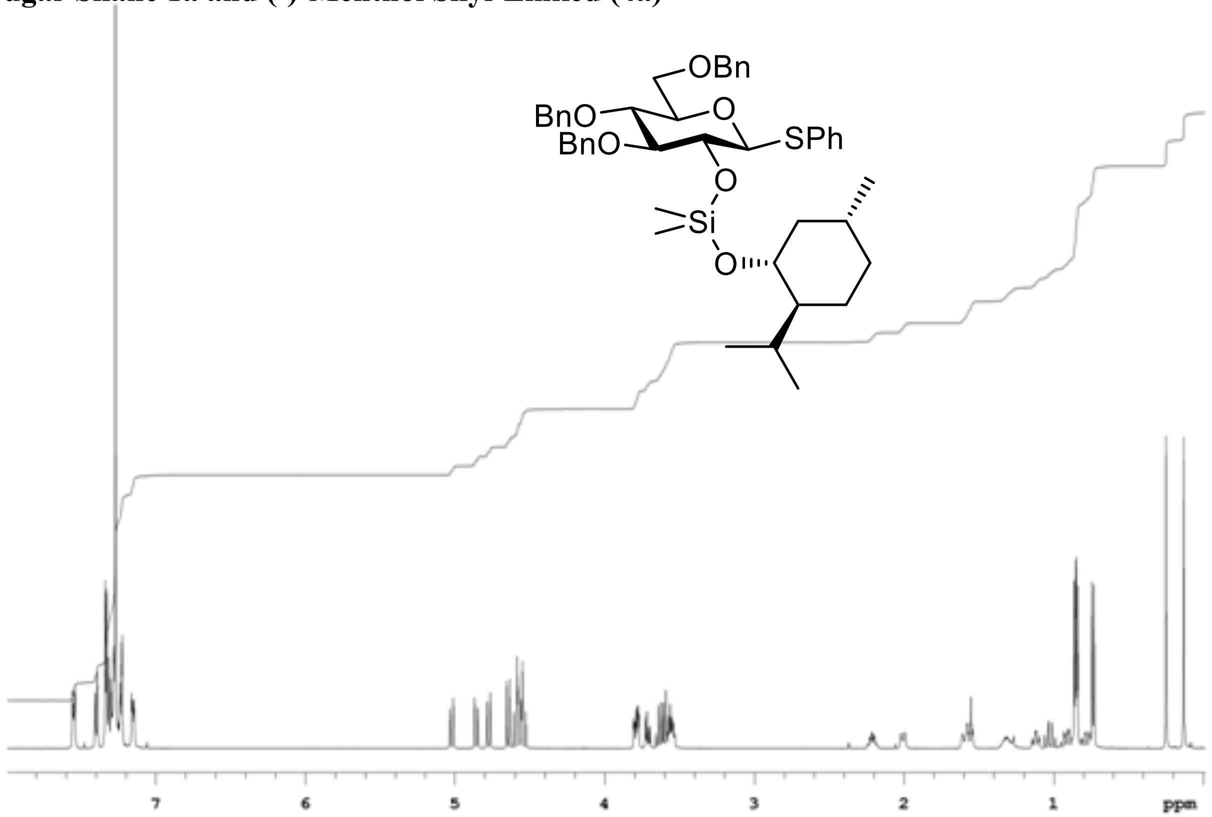

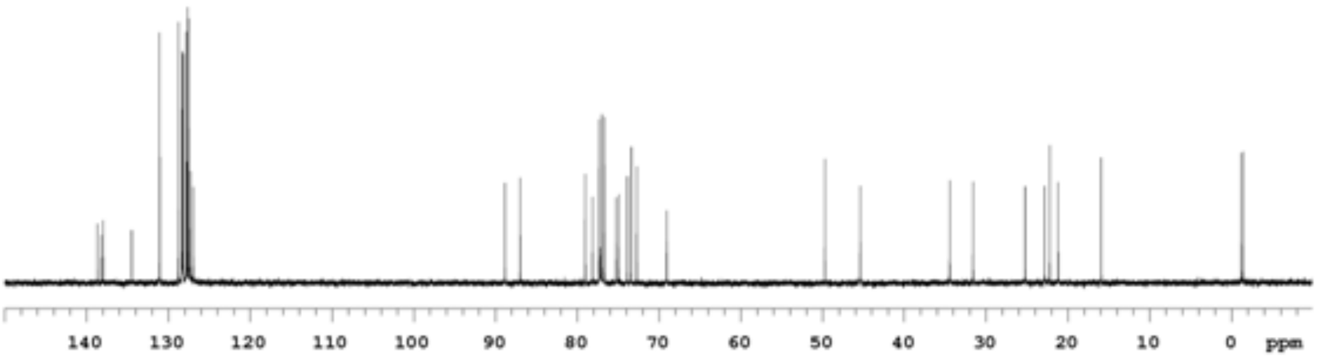

Sugar Silane 1a and (-)-Menthol Glycoside (5a)

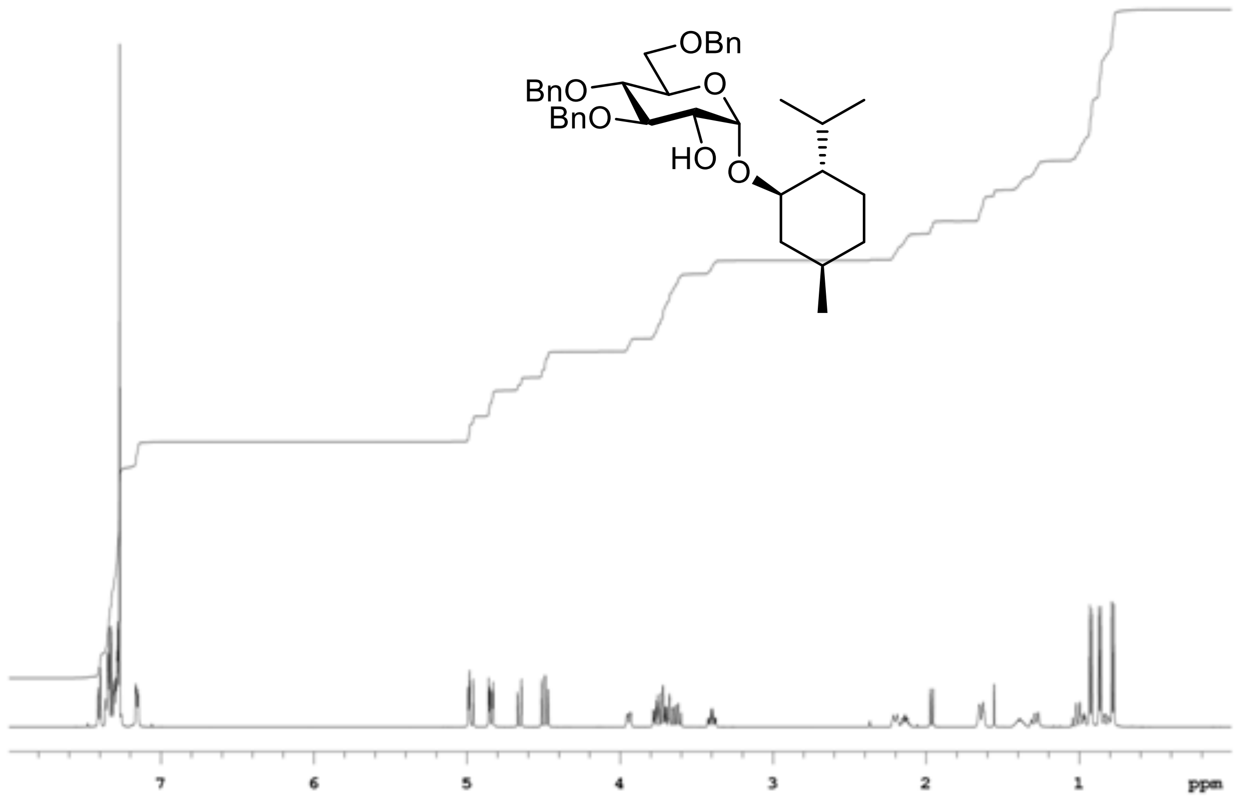

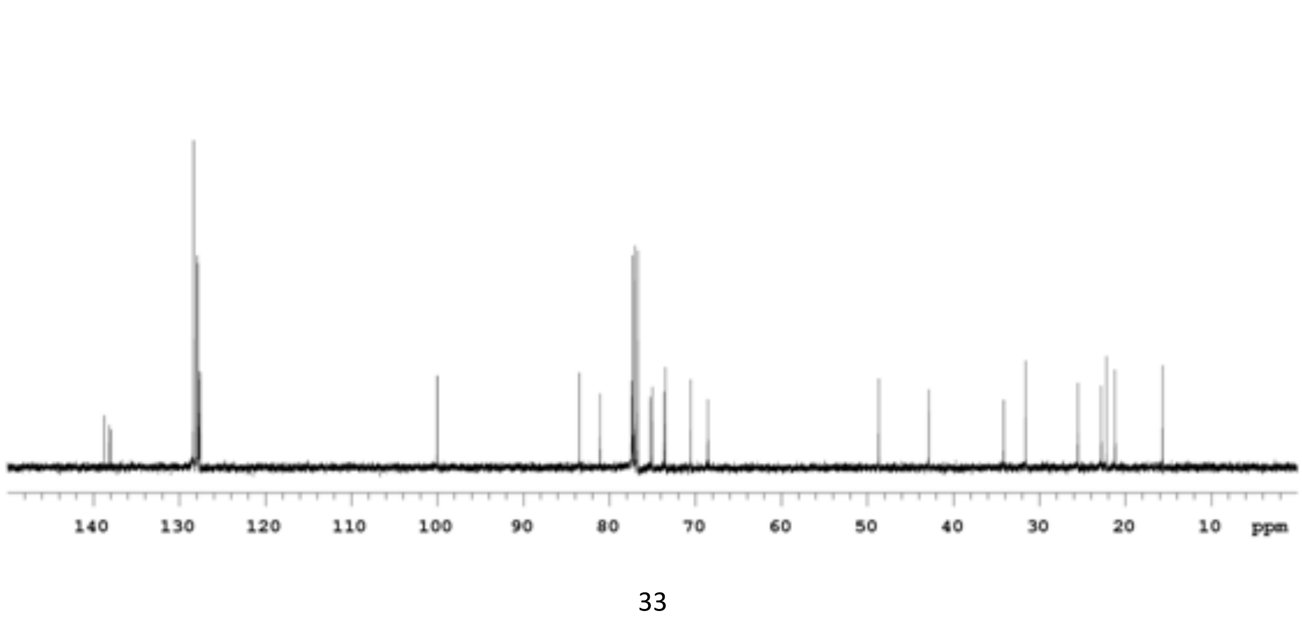

Sugar Silane 2a and (-)-Menthol Silyl-Linked (4b)

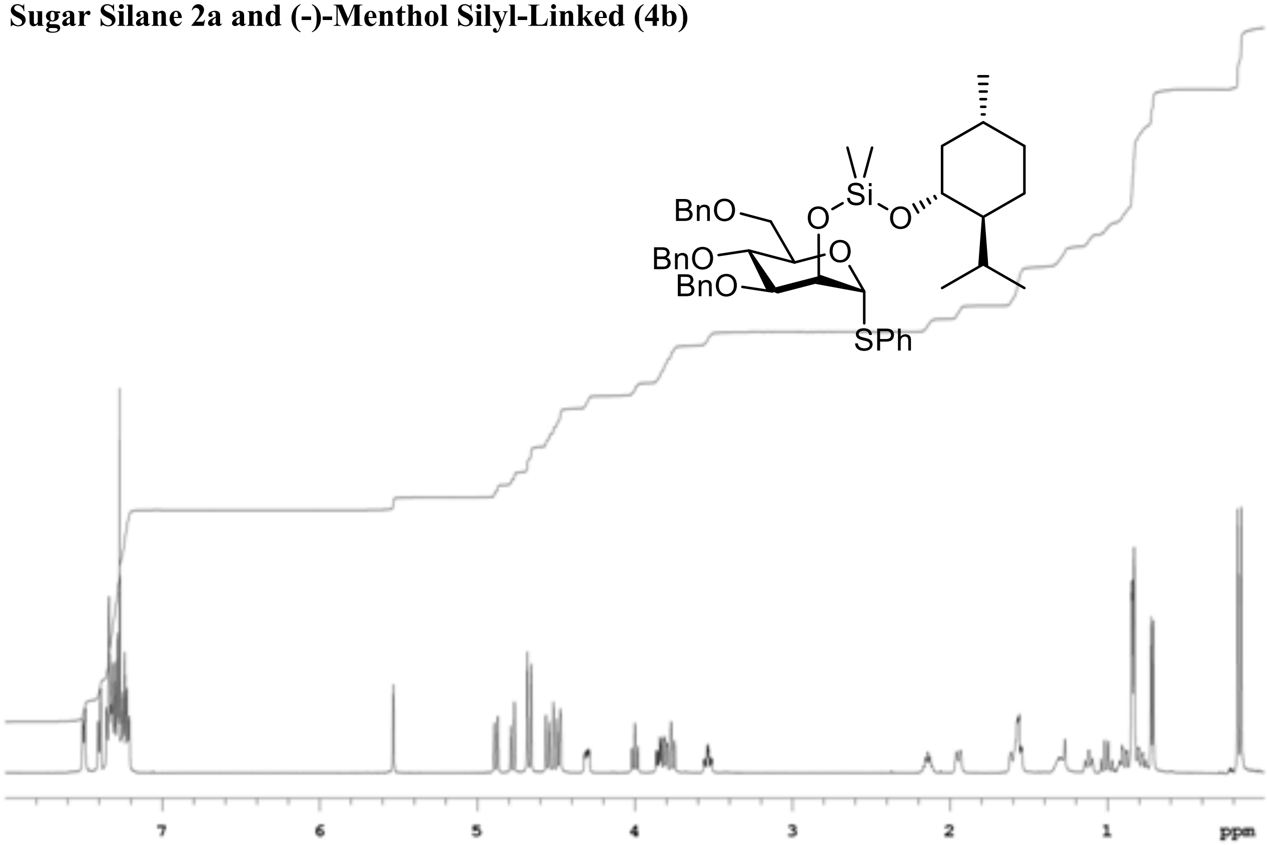

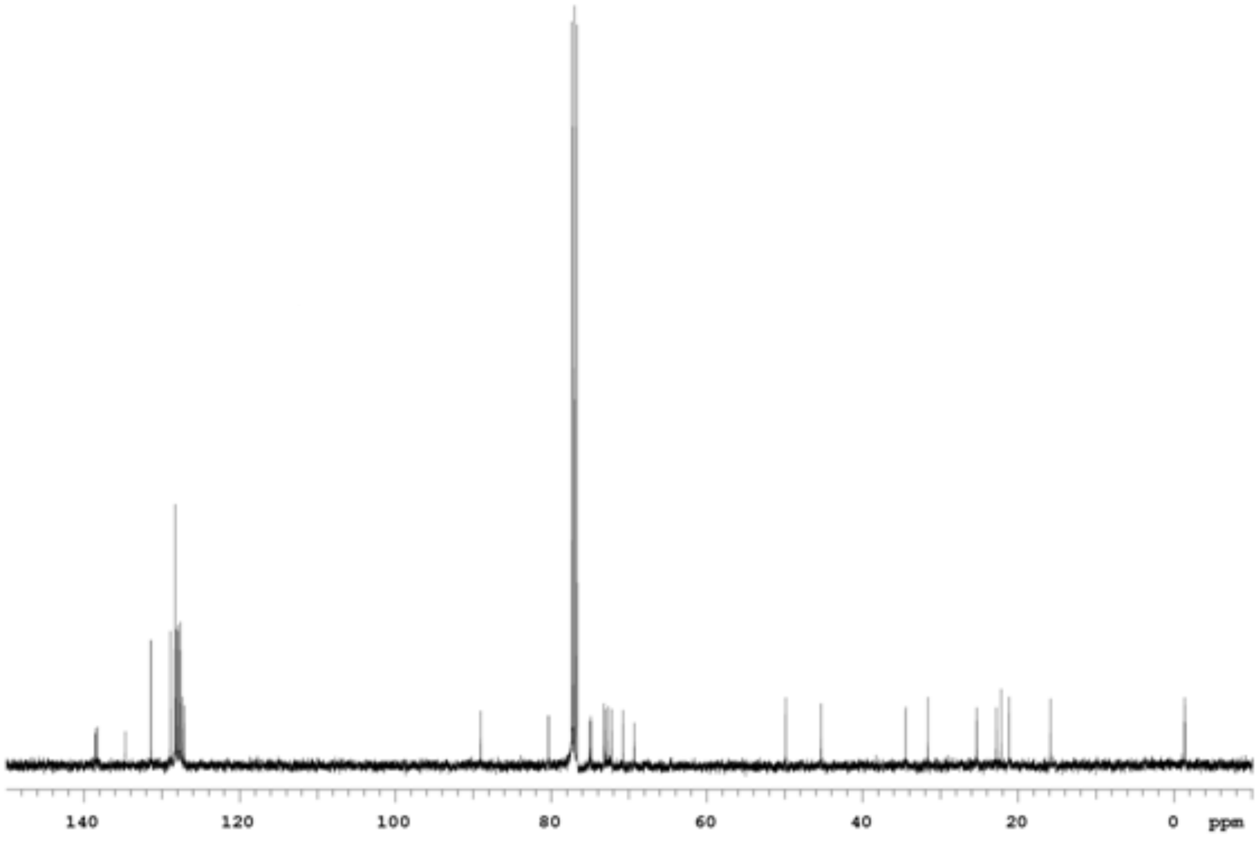

Sugar Silane 3 and (-)-Menthol Silyl-Linked (4c)

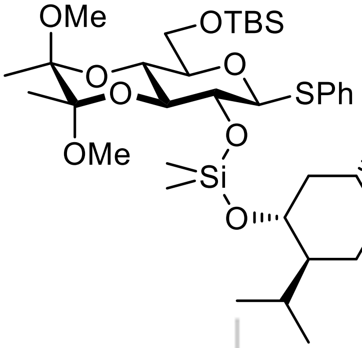

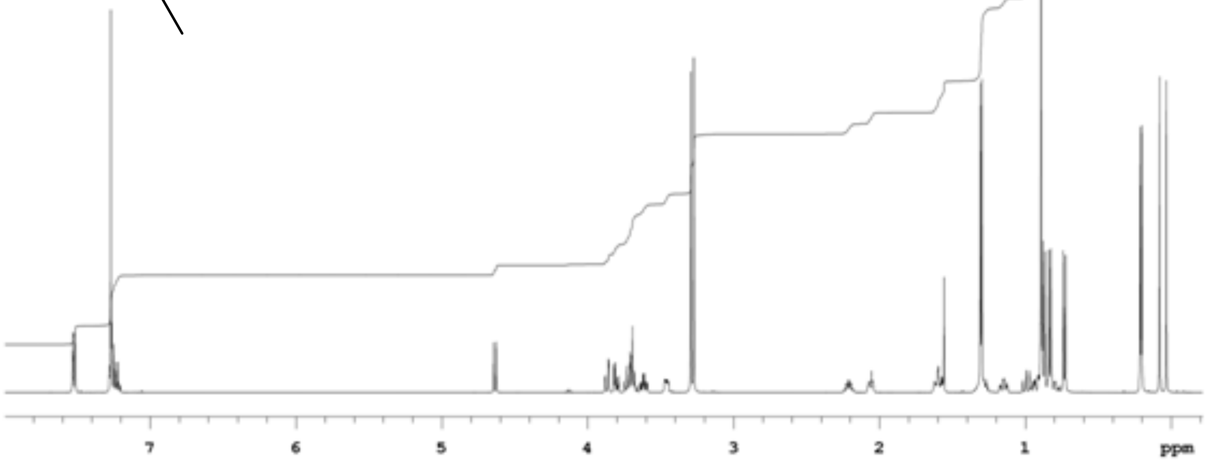

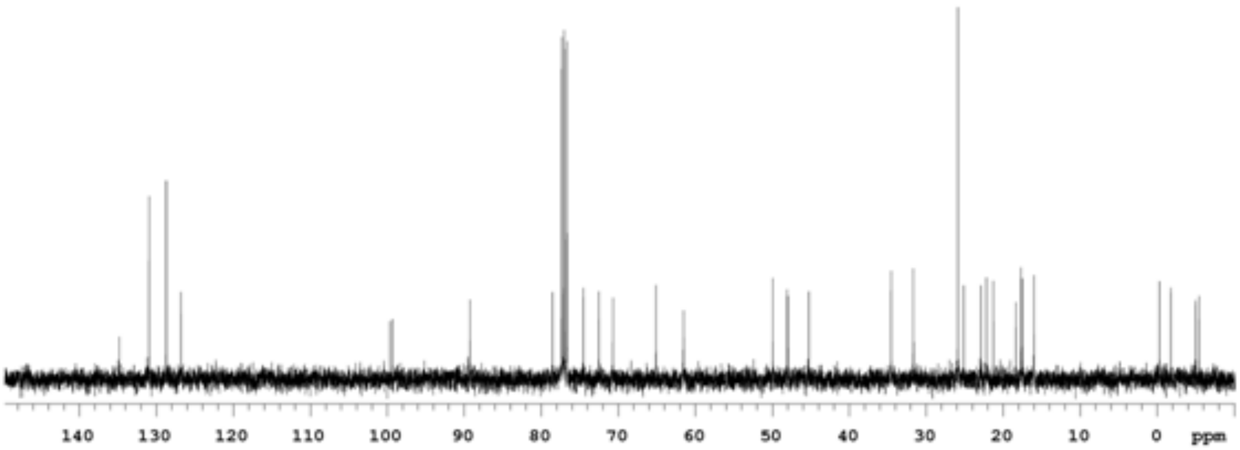

Sugar Silane 3 and (-)-Menthol Glycoside (5c)

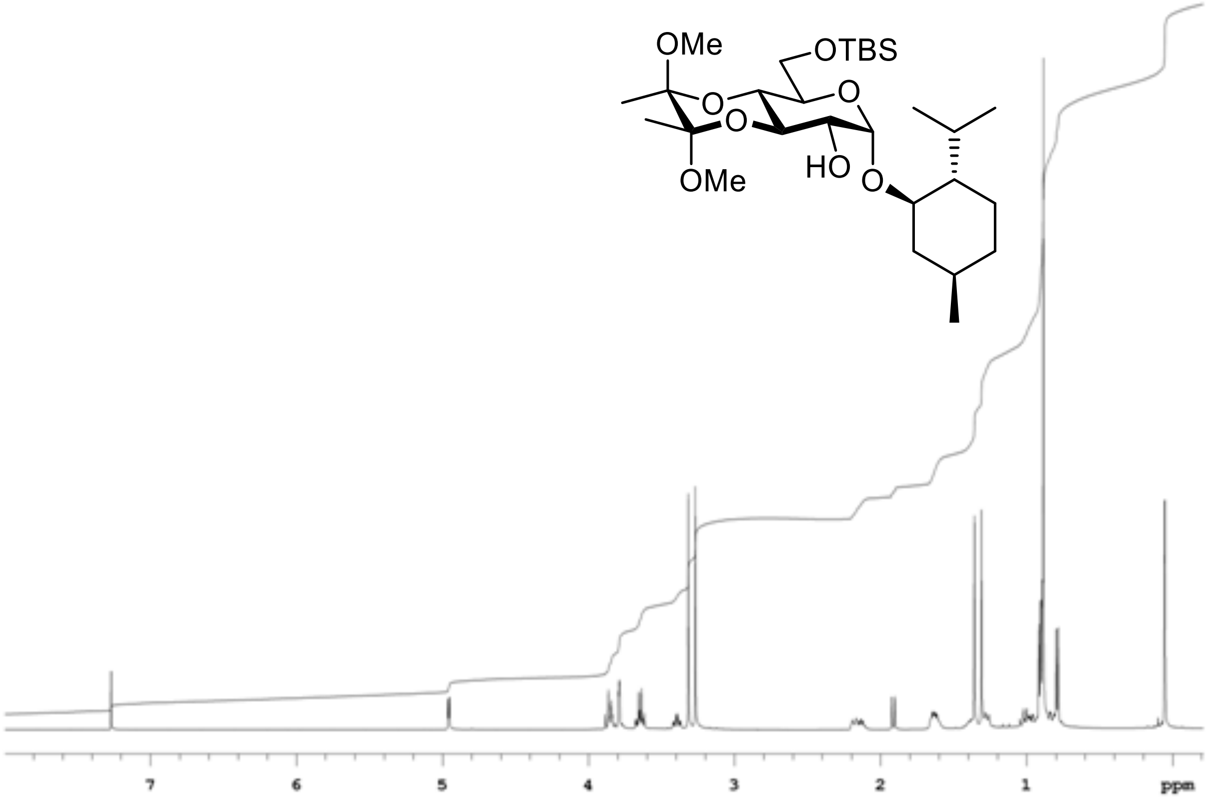

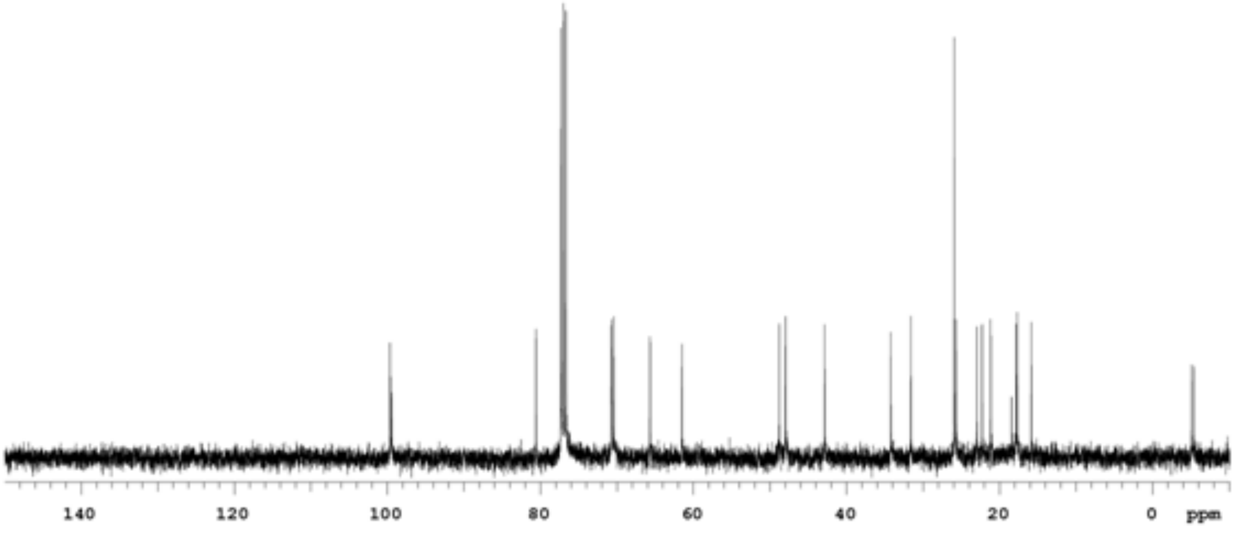

Sugar Silane 1b and 1,2:3,4-*O*-Diisopropylidene-D-Galactopyranose Silyl-Linked (4d)

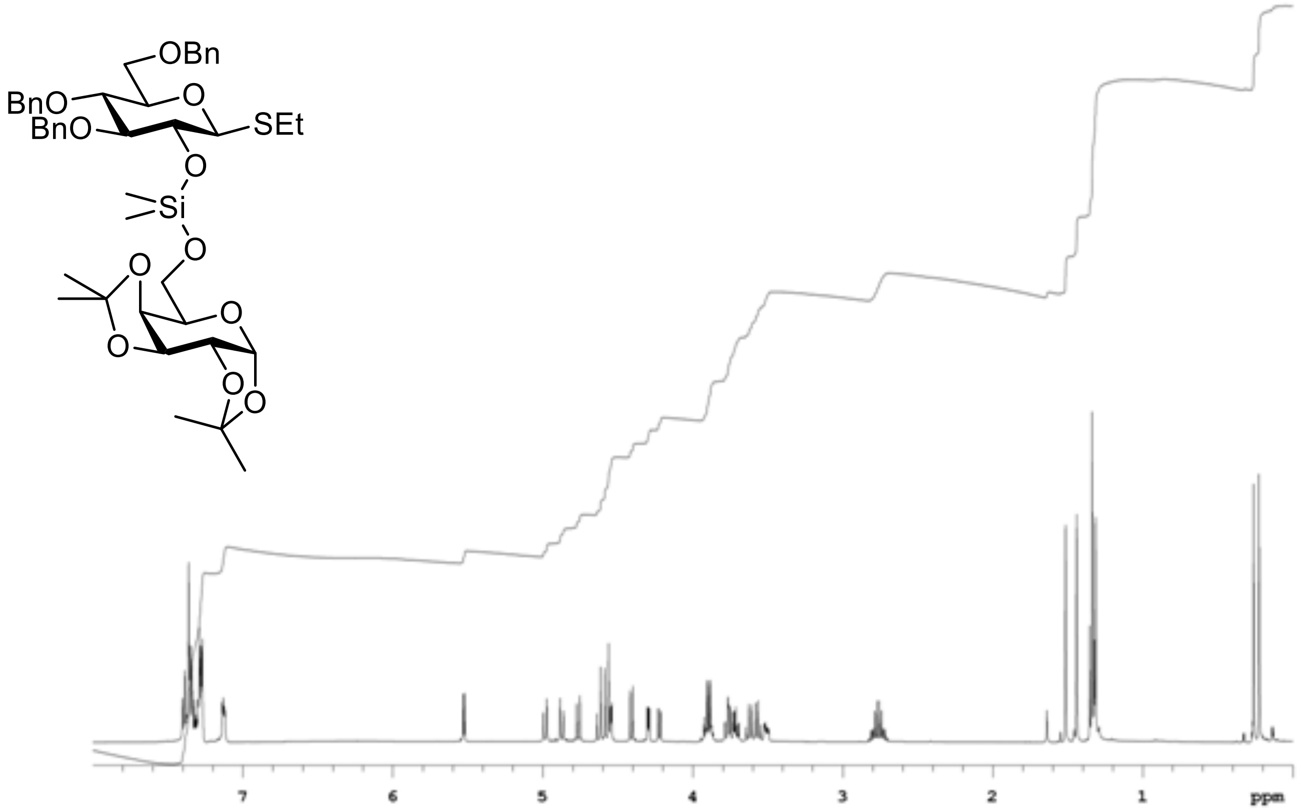

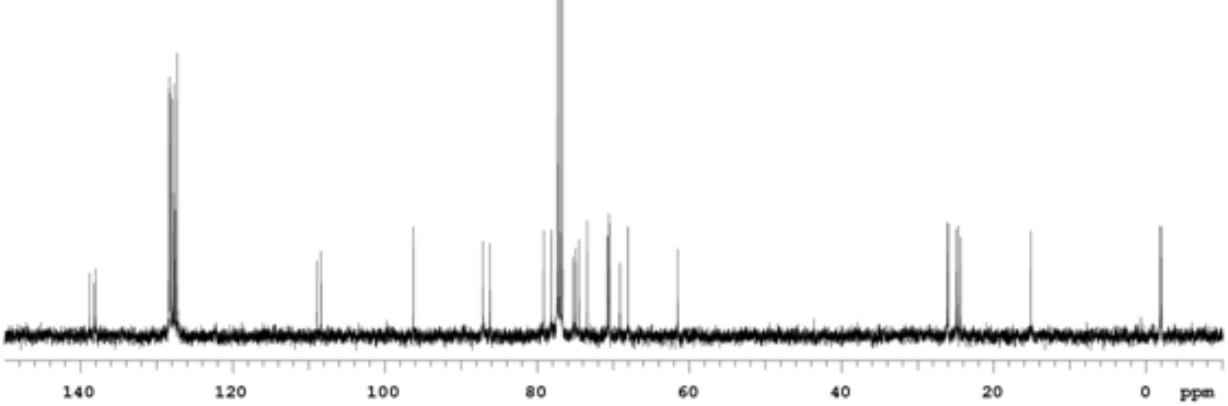

Sugar Silane 1b and 1,2:3,4-*O*-Diisopropylidene-D-Galactopyranose Glycoside (5d)

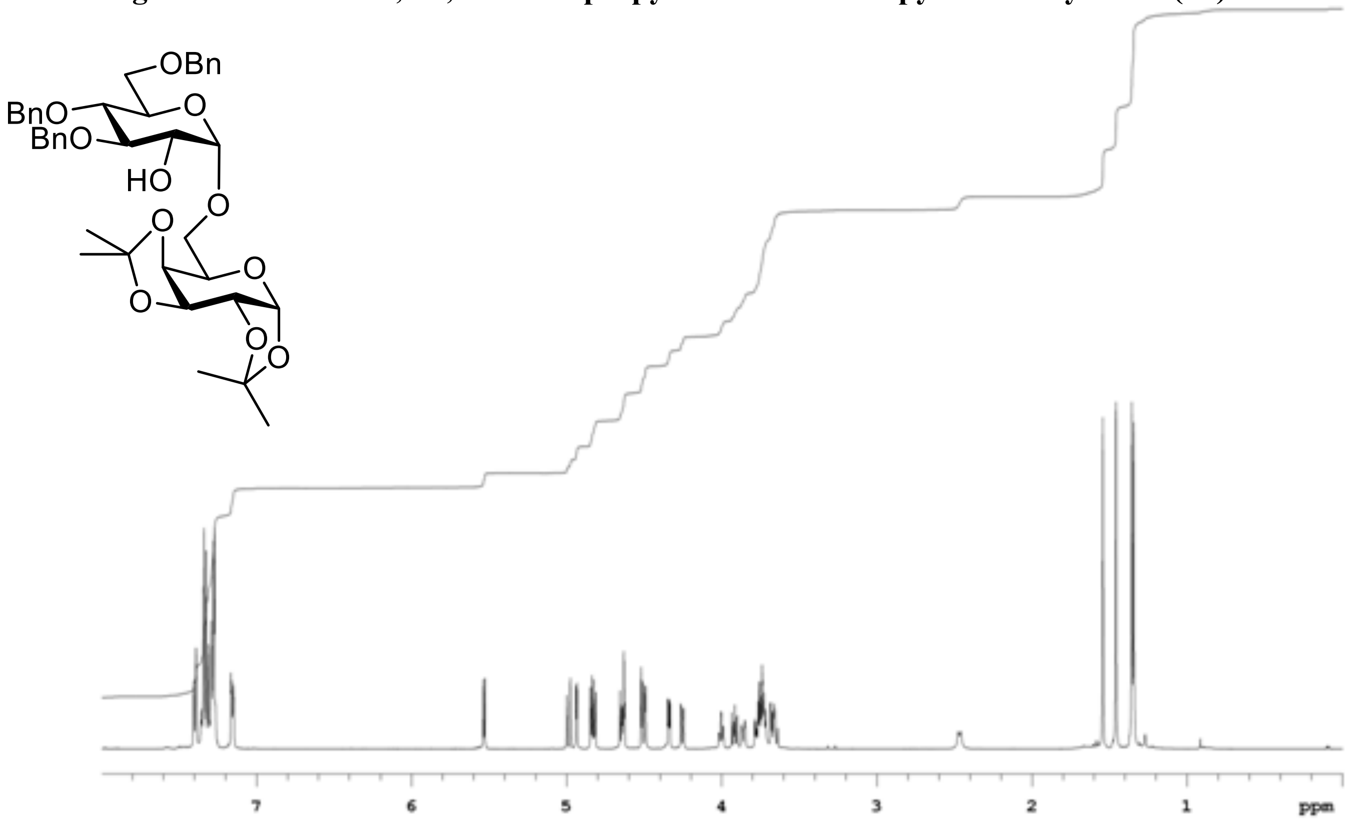

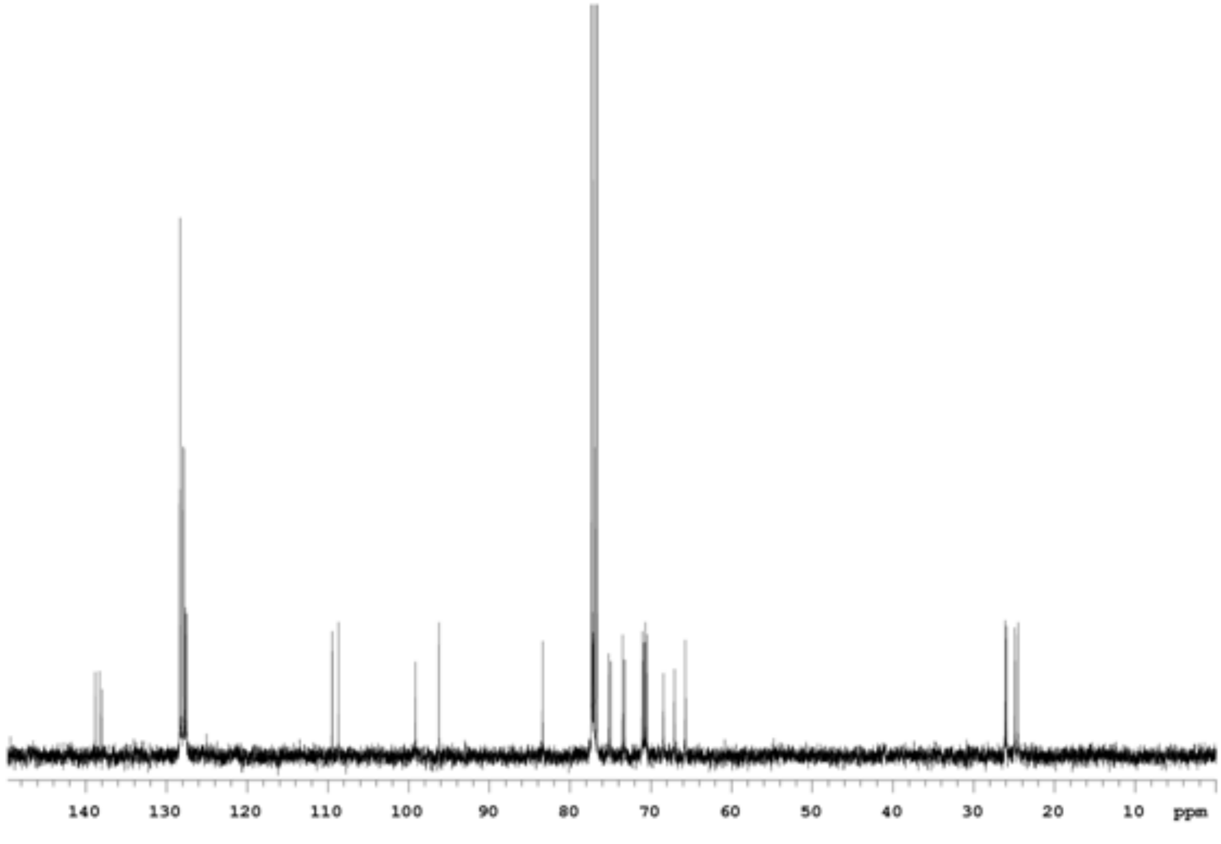

Sugar Silane 1b and Glycoside 5b Silyl-Linked (4e)

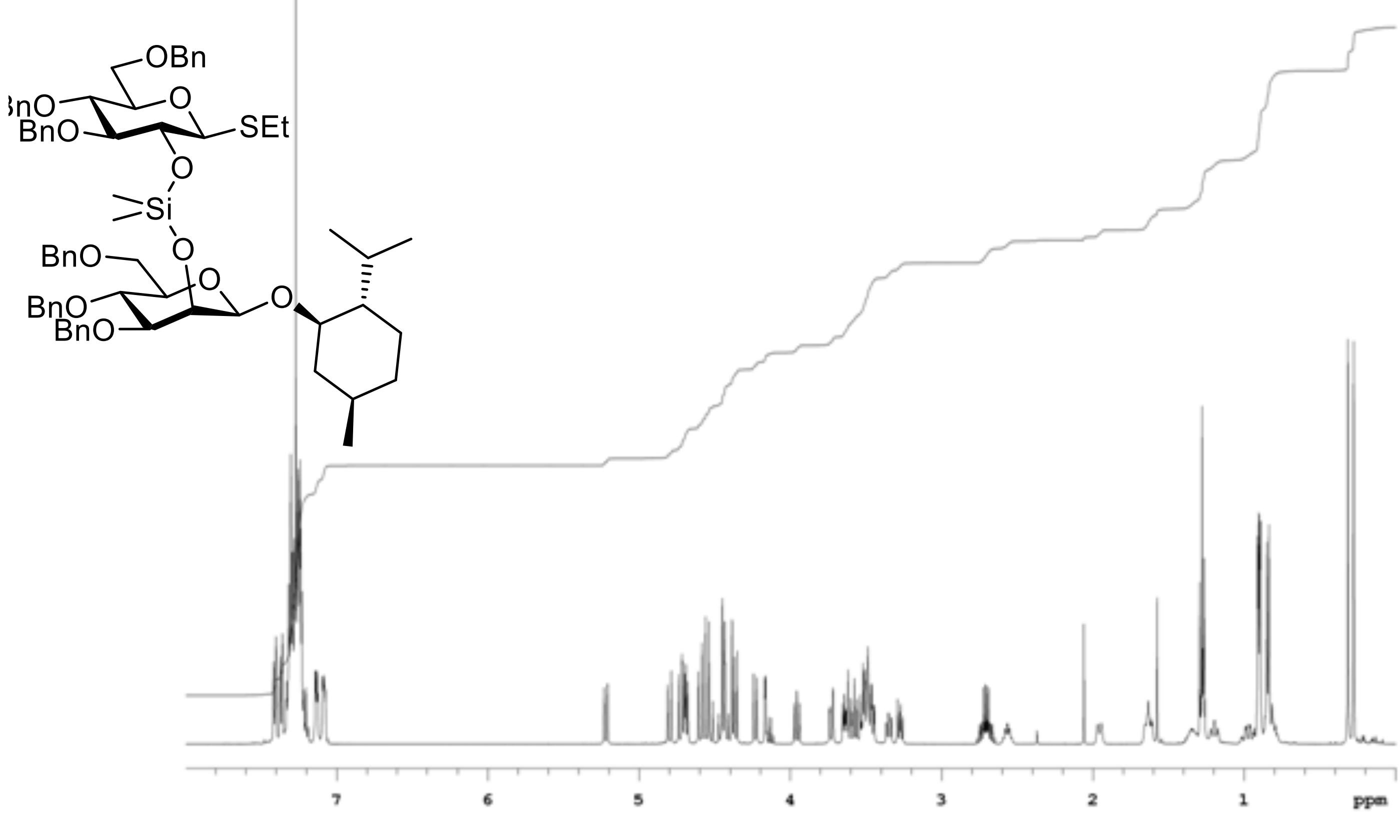

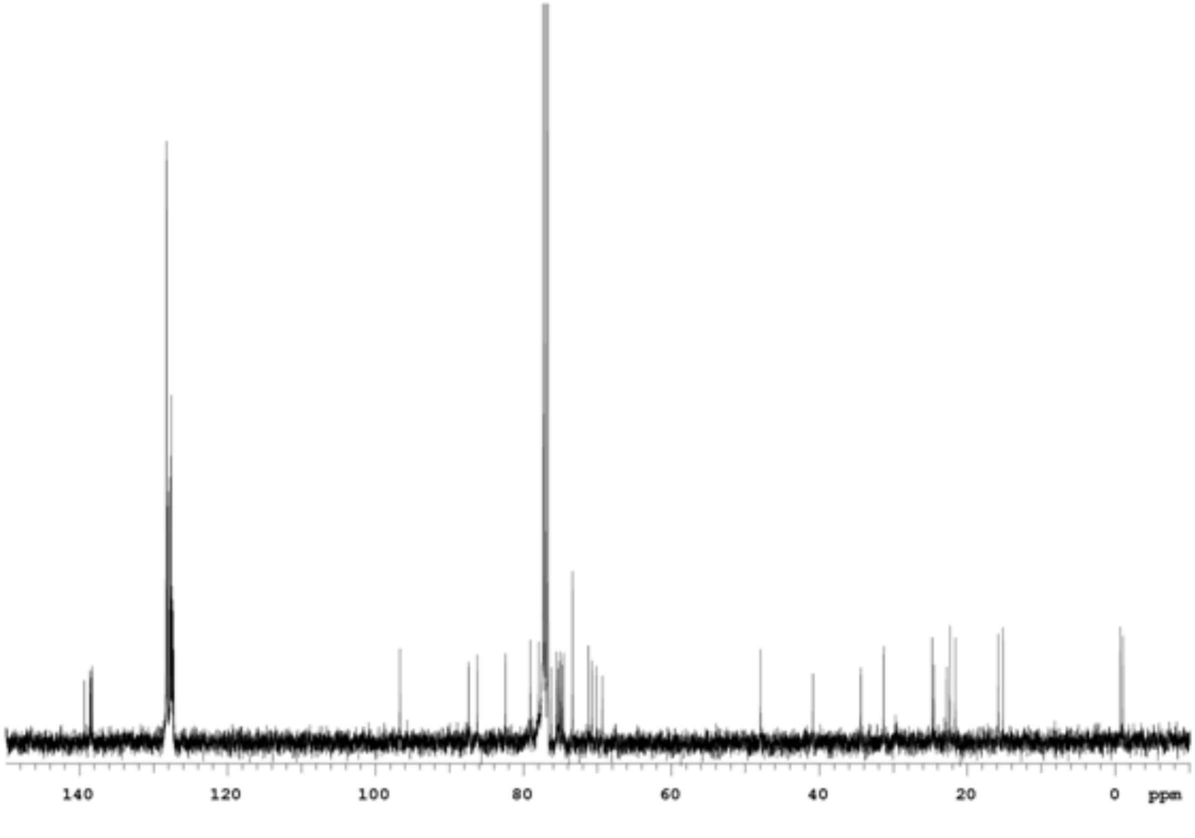

Sugar Silane 1b and Glycoside 5b Glycoside (5e)

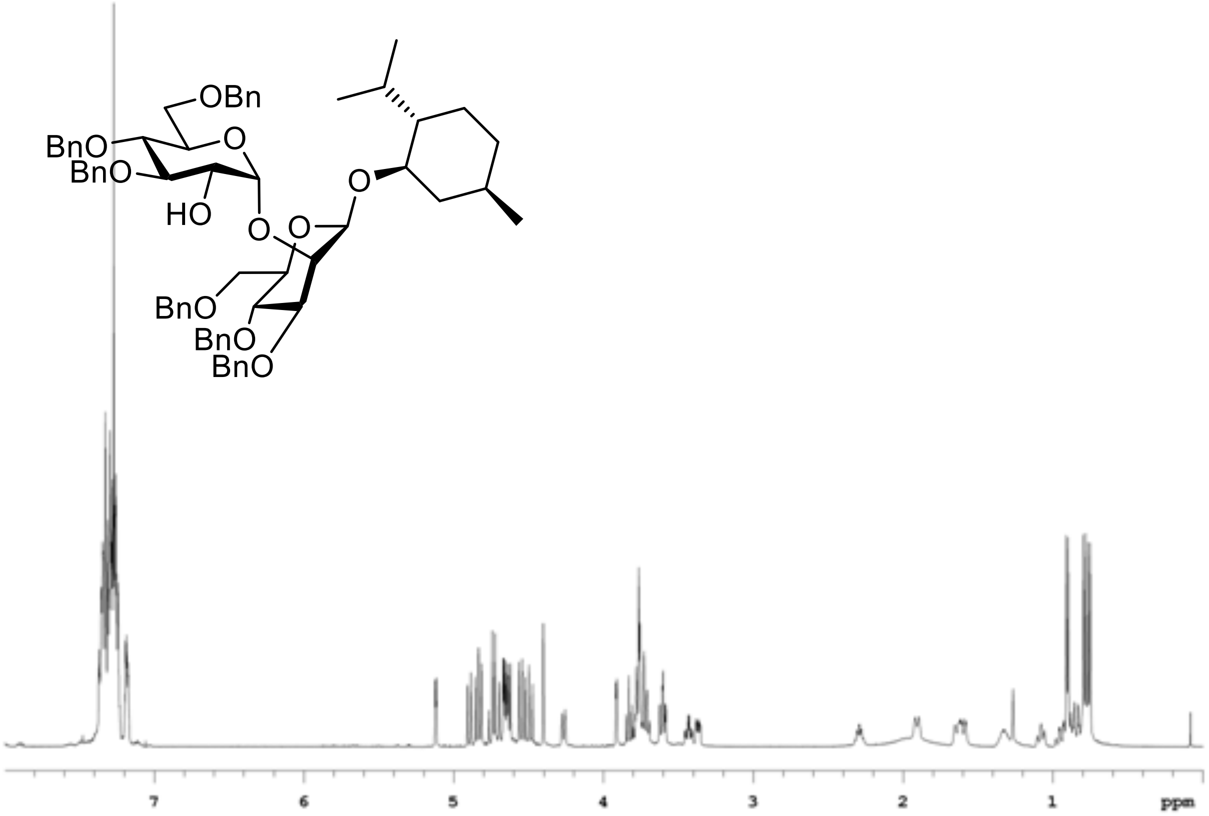

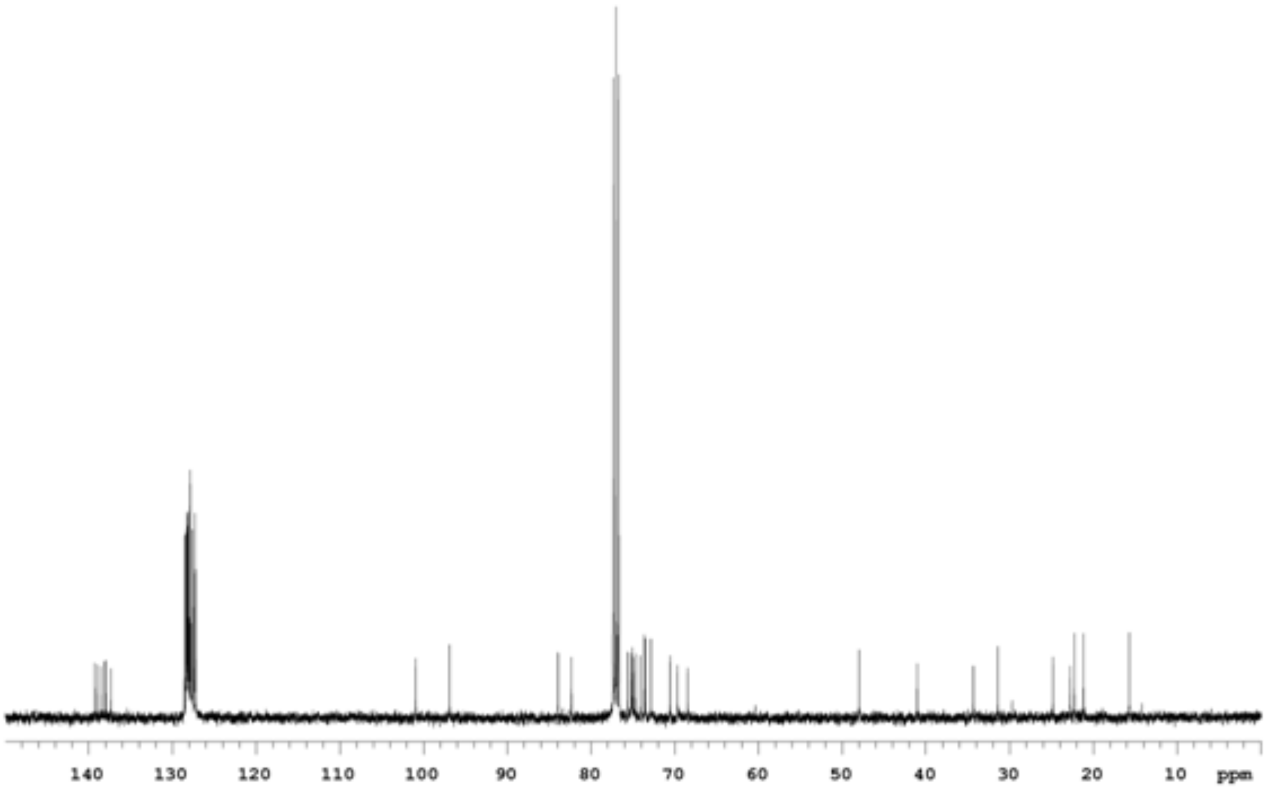

Sugar Silane 1a and (1*S*,2*S*,5*S*)-(-)-2-Hydroxy-3-Pinanone Silyl-Linked (4f)

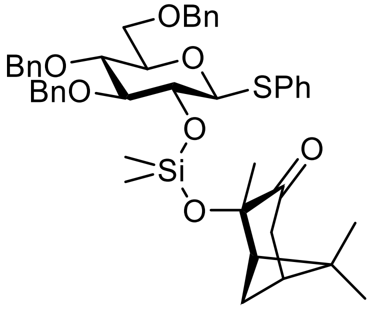

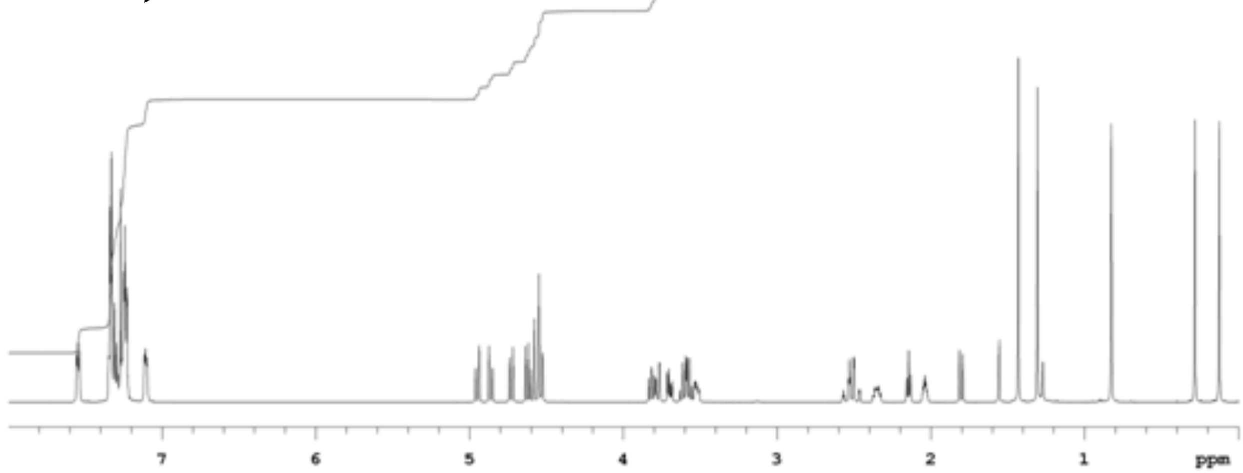

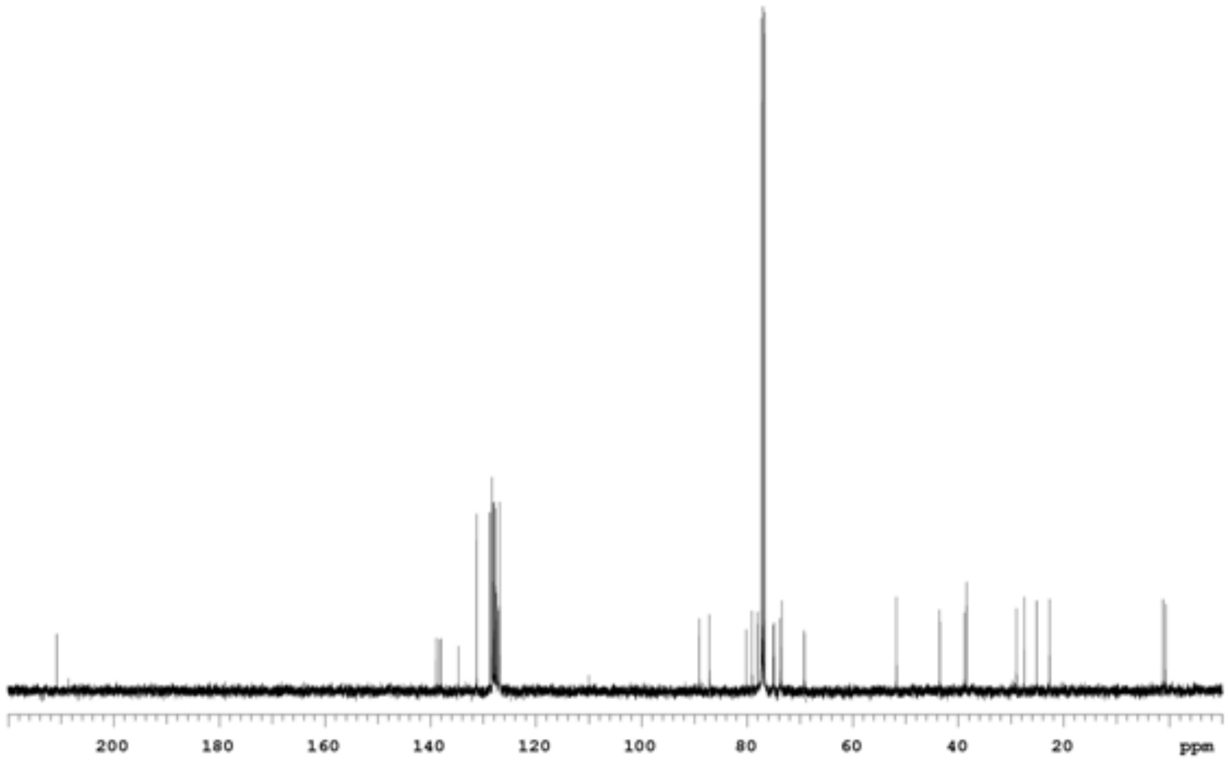

Sugar Silane 1a and (1*S*,2*S*,5*S*)-(-)-2-Hydroxy-3-Pinanone Glycoside (5f)

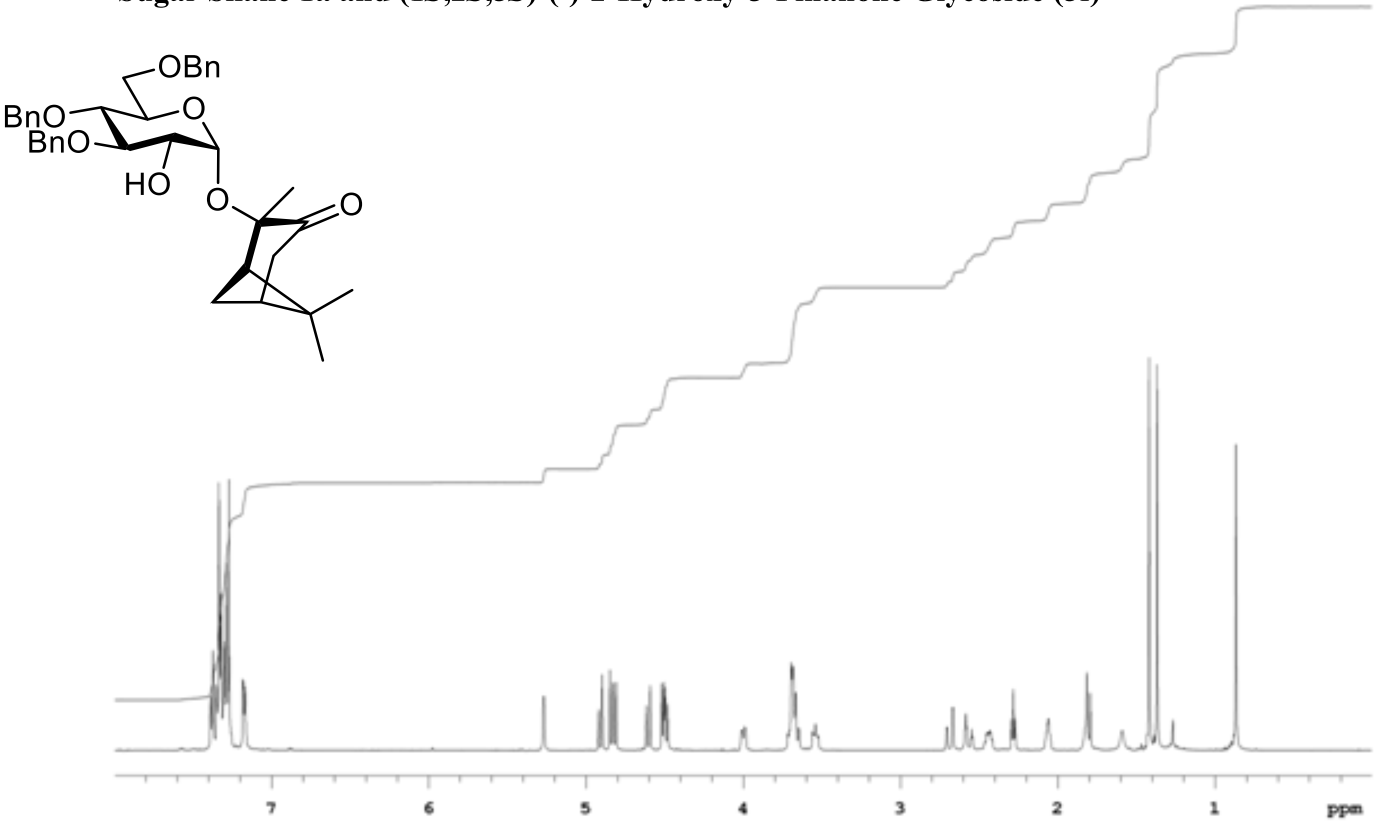

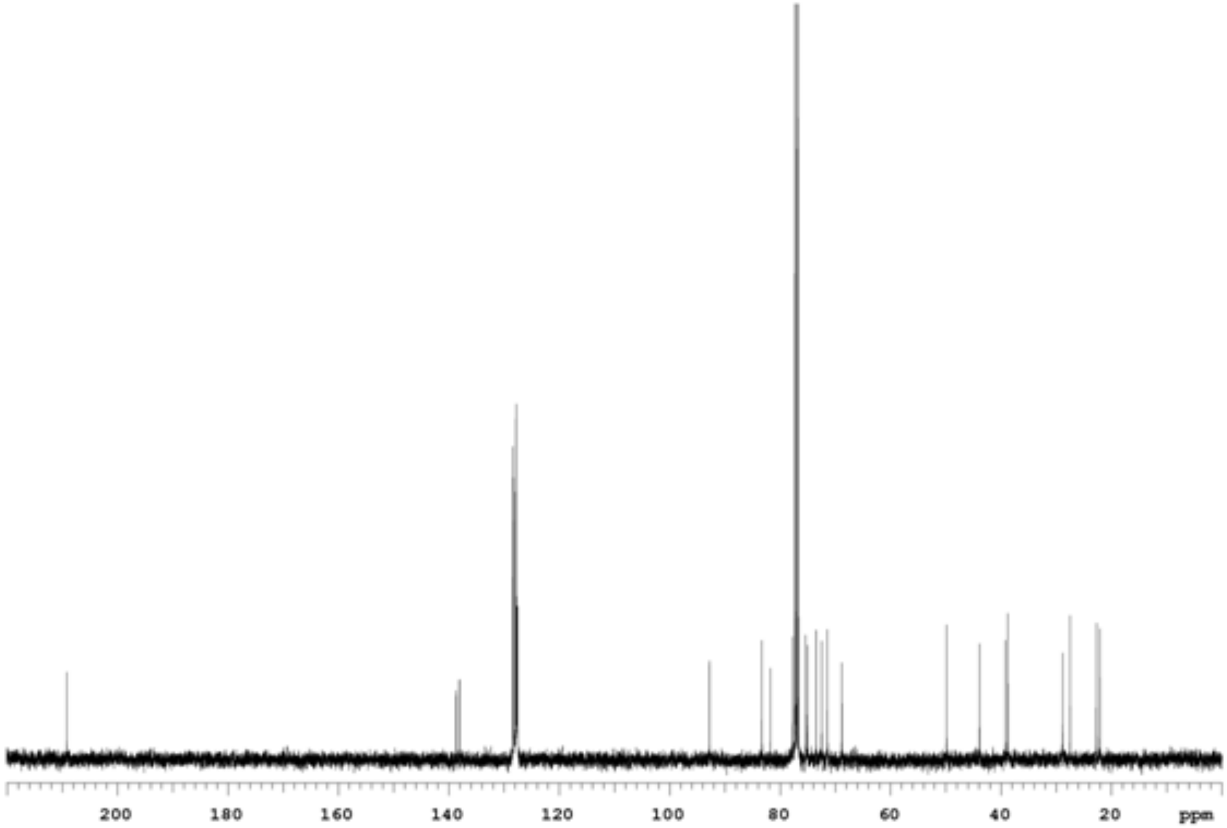

Sugar Silane 2a and (1*S*,2*S*,5*S*)-(-)-2-Hydroxy-3-Pinanone Silyl-Linked (4g)

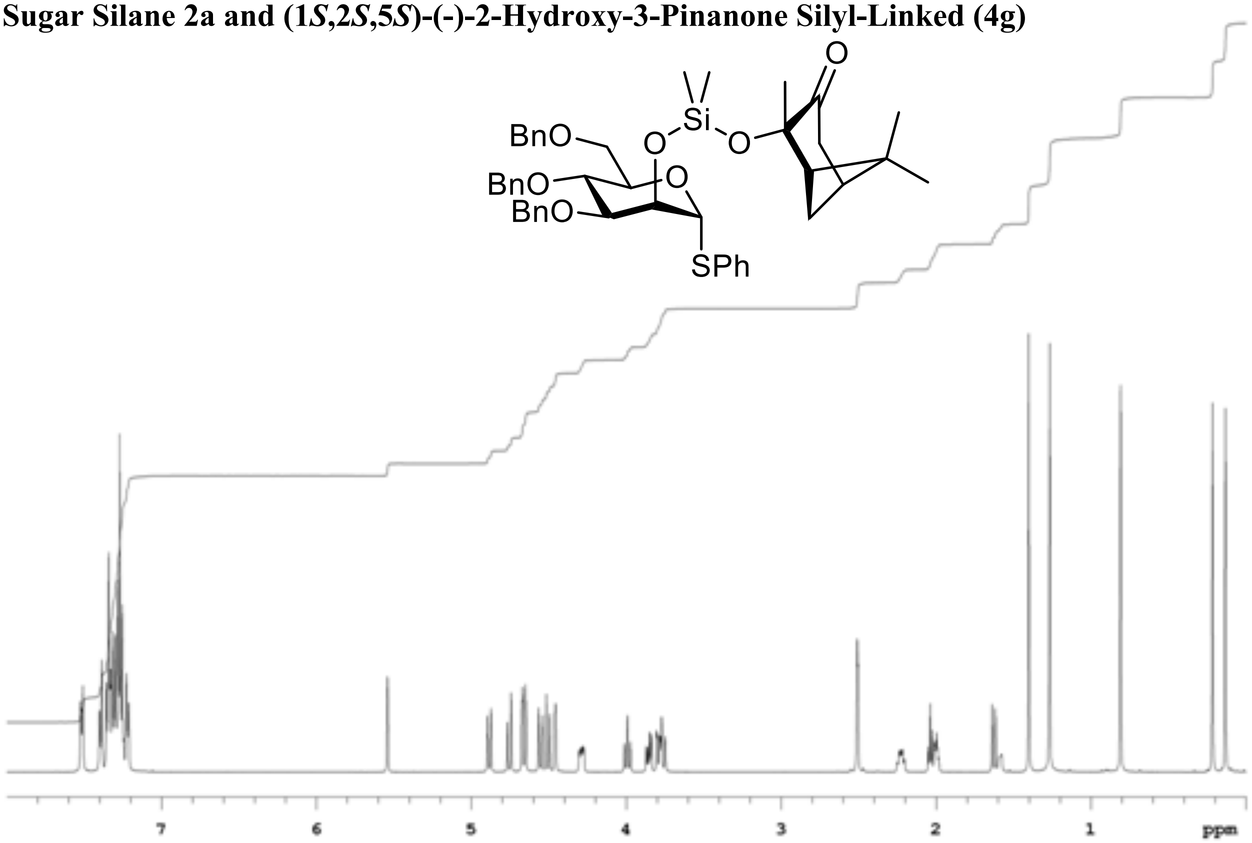

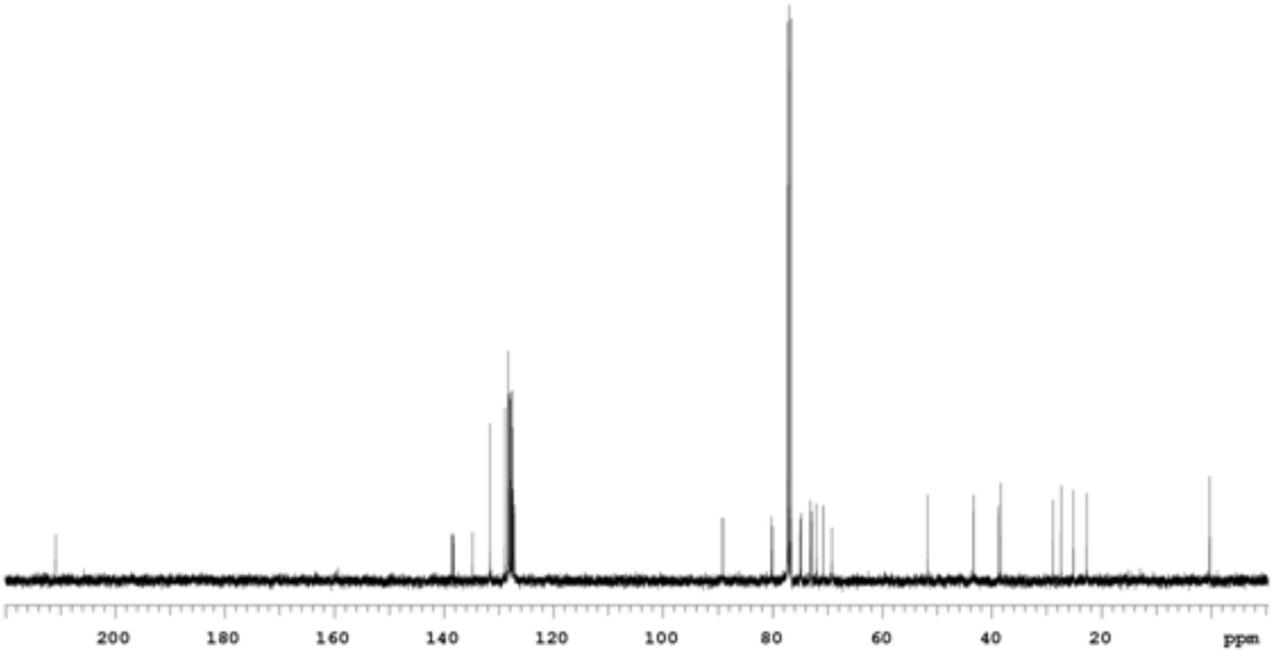

Sugar Silanes 2a and (1*S*,2*S*,5*S*)-(-)-2-Hydroxy-3-Pinanone Glycoside (5g)

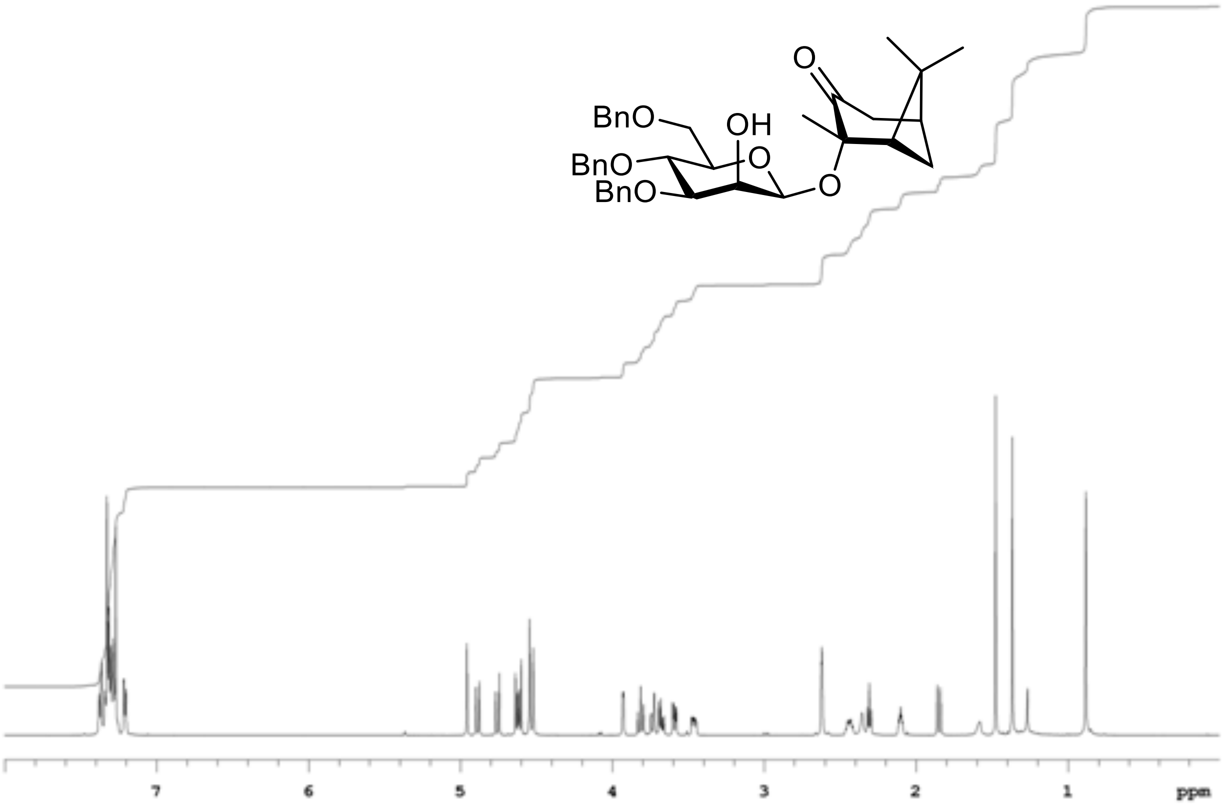

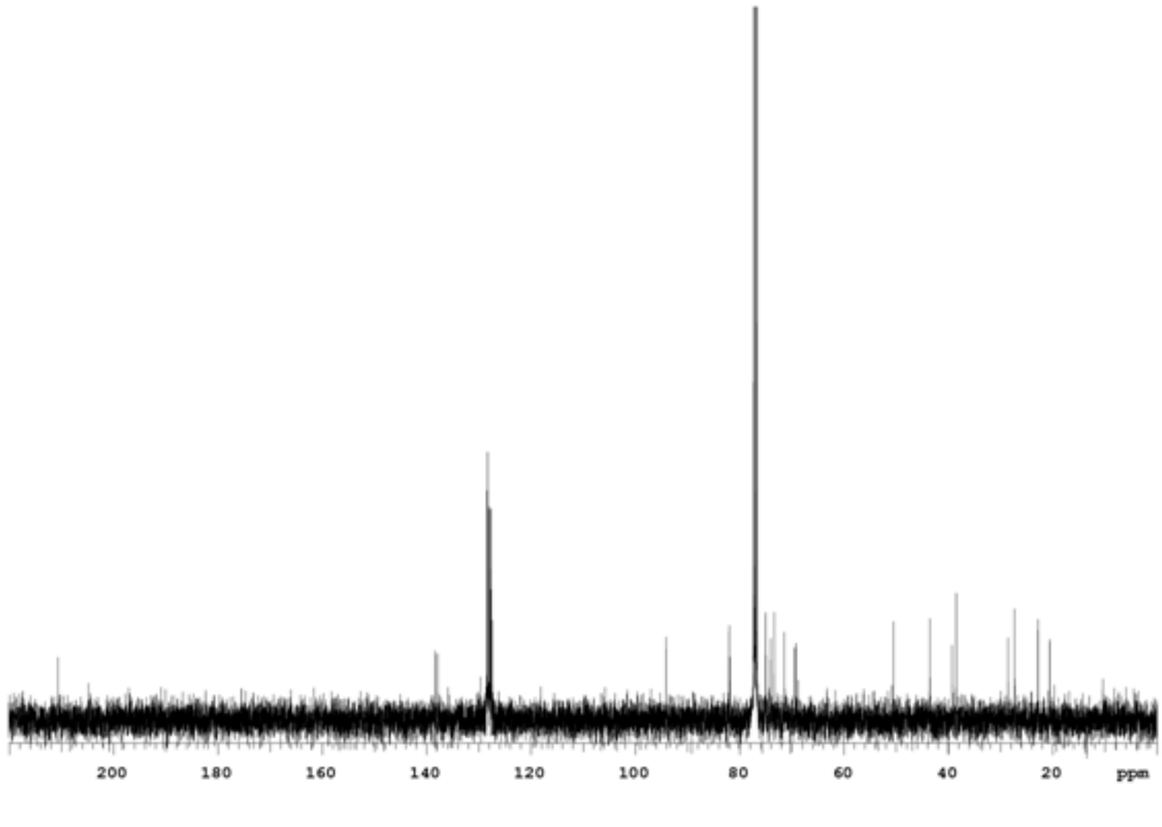

Sugar Silane 1b and Ketosteroid Silyl-Linked (4h)

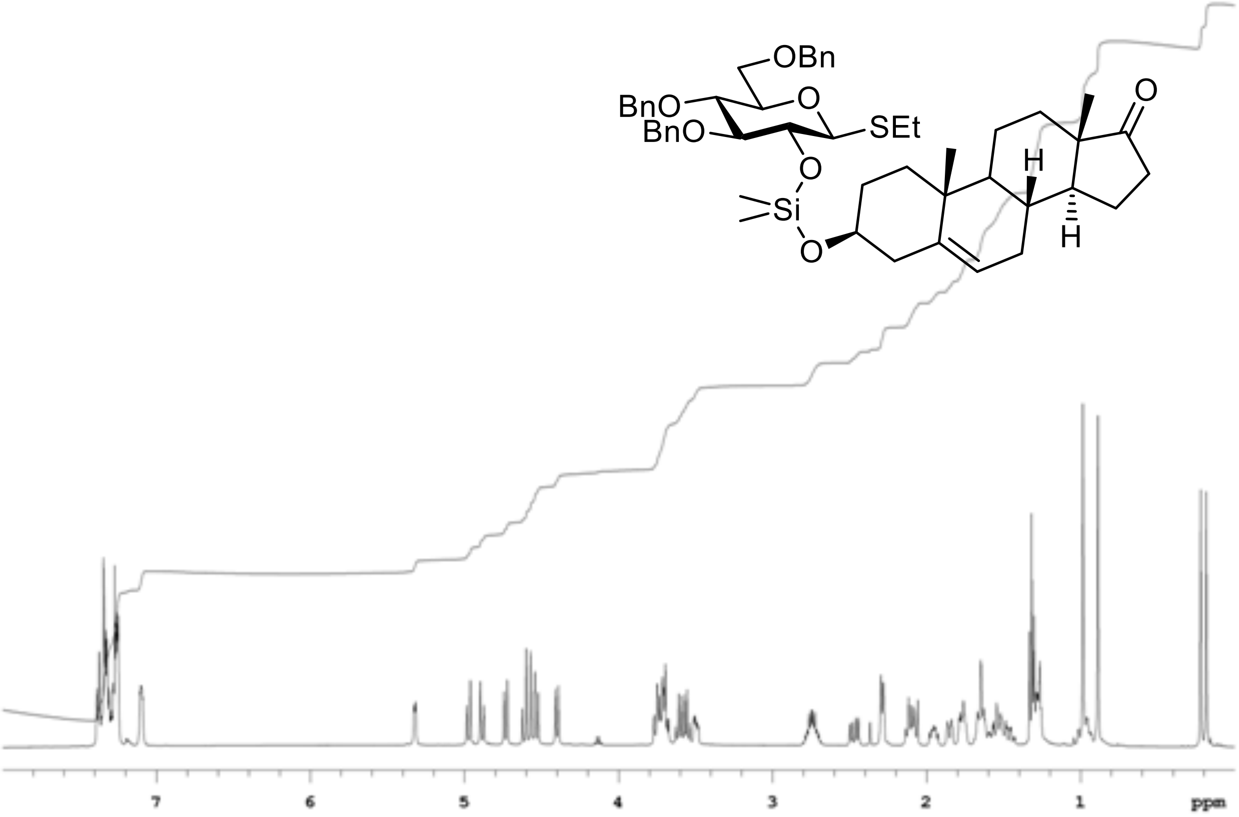

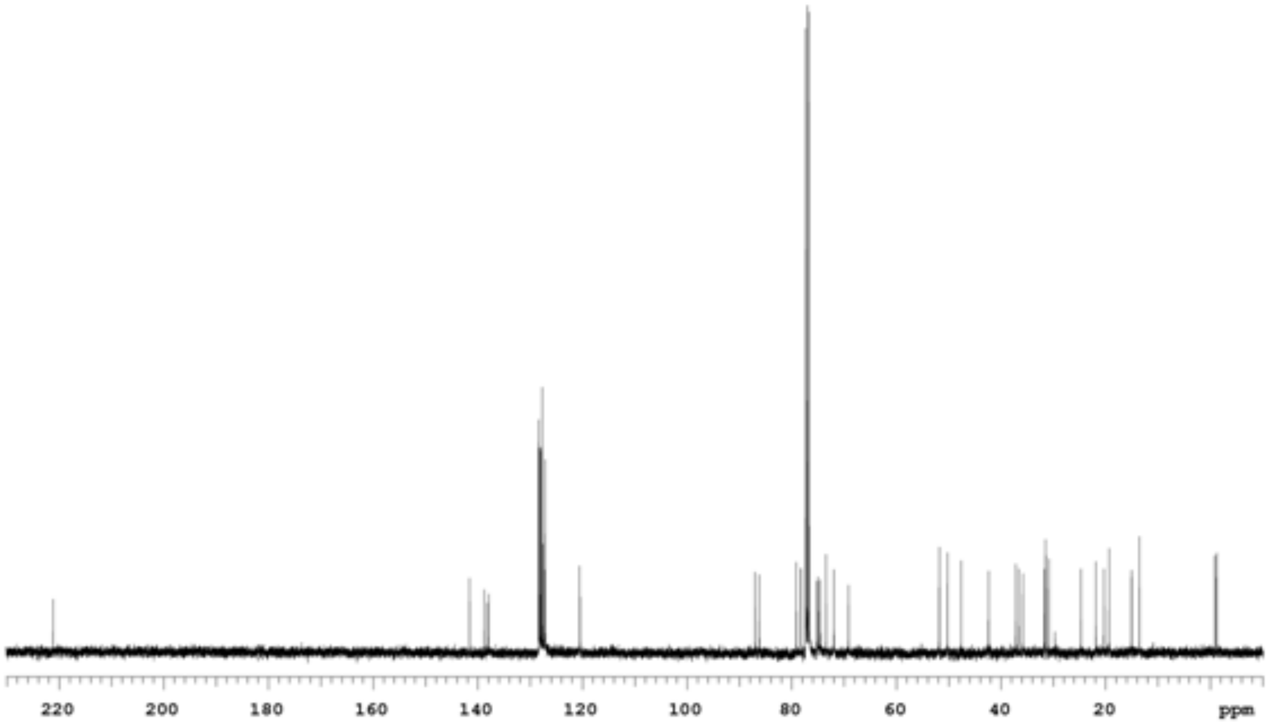

Sugar Silane 1b and Ketosteroid Glycoside (5h)

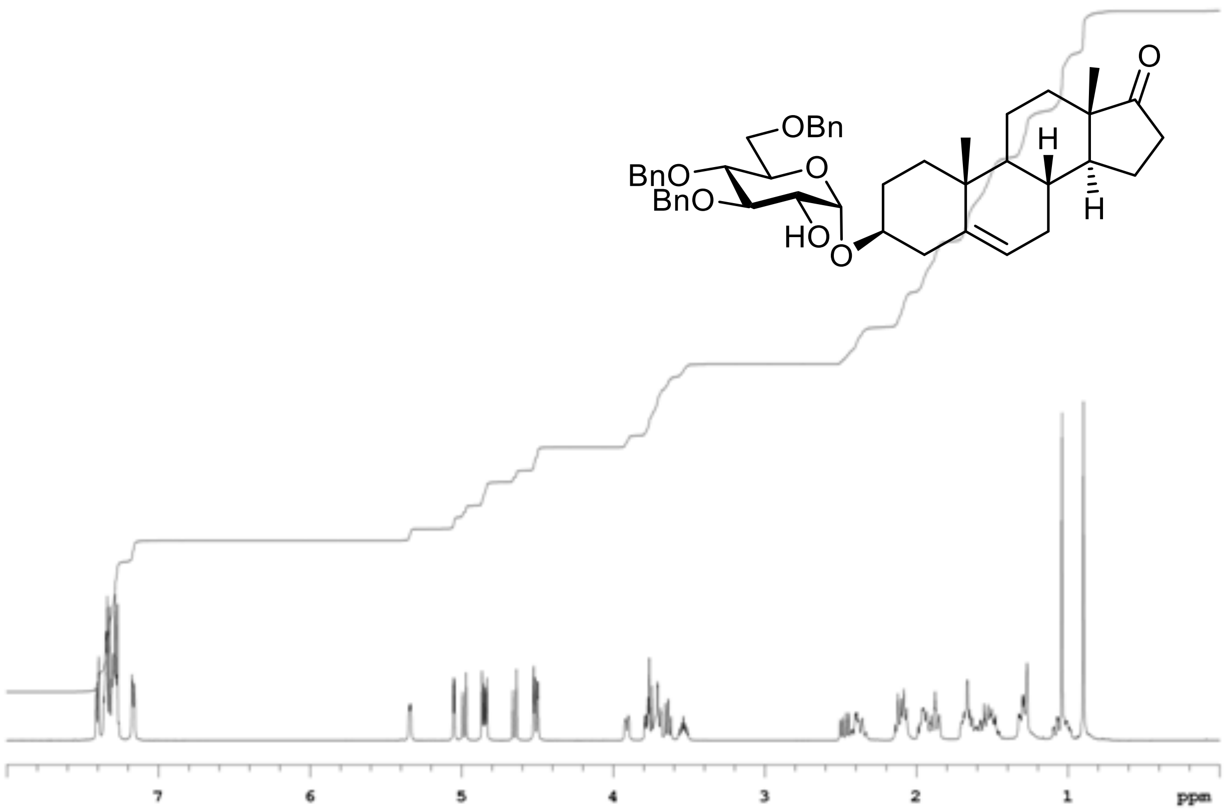

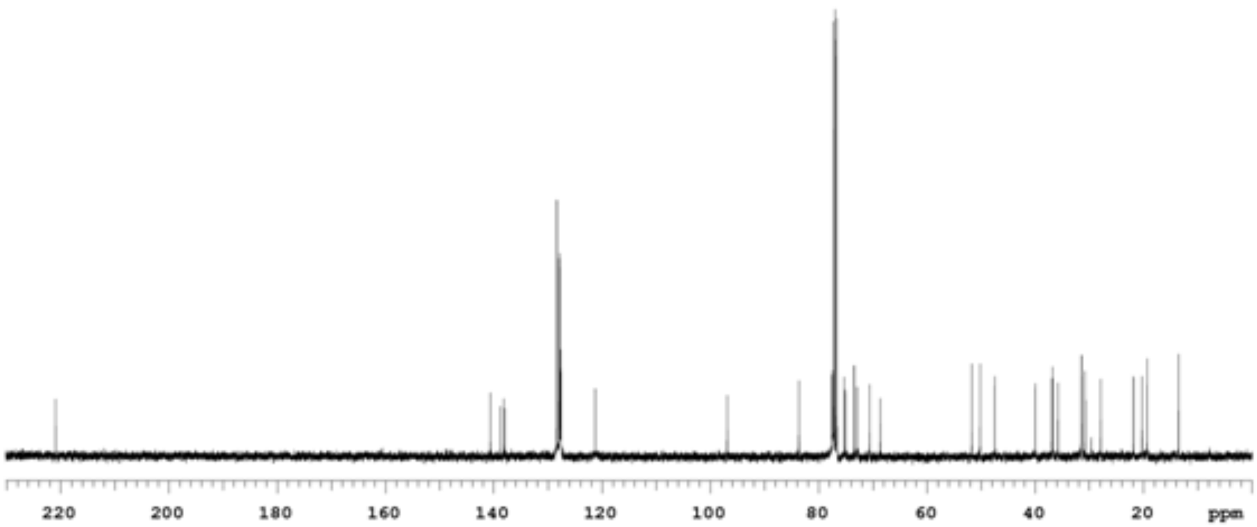

Sugar Silane 1a and Methyl 2,3-Di-*O*-Acetyl-Glucoside Silyl-Linked (4i)

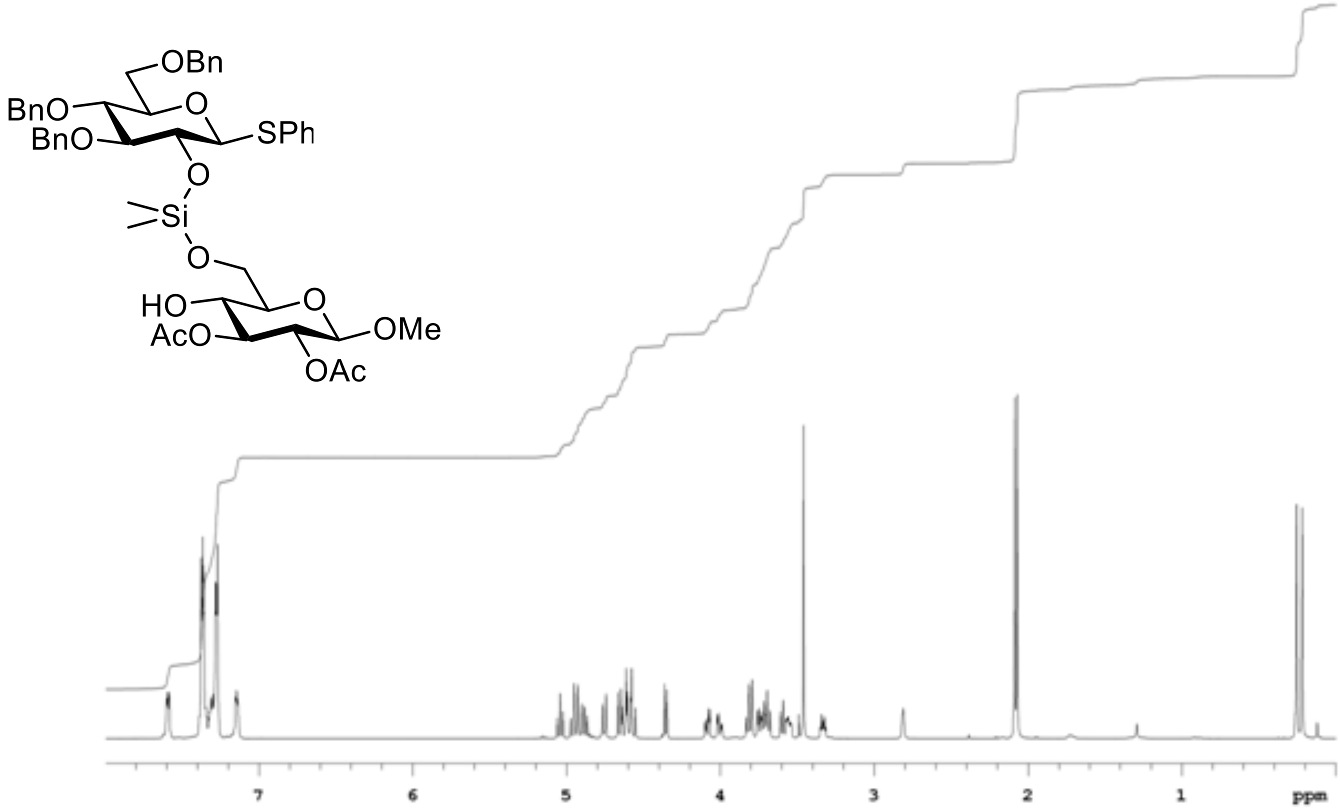

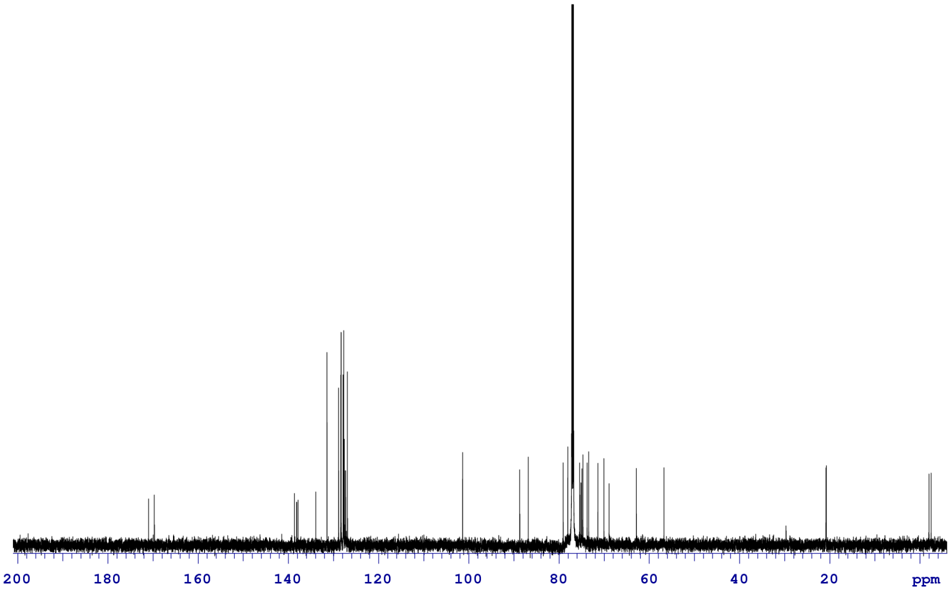

Sugar Silane 1a and Methyl 2,3-Di-*O*-Acetyl-Glucoside Glycoside (5i)

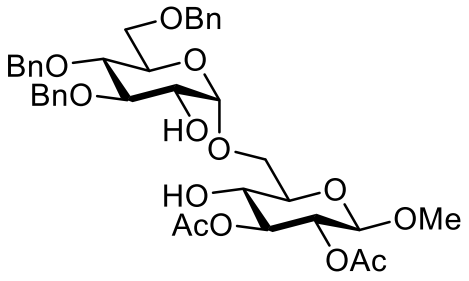

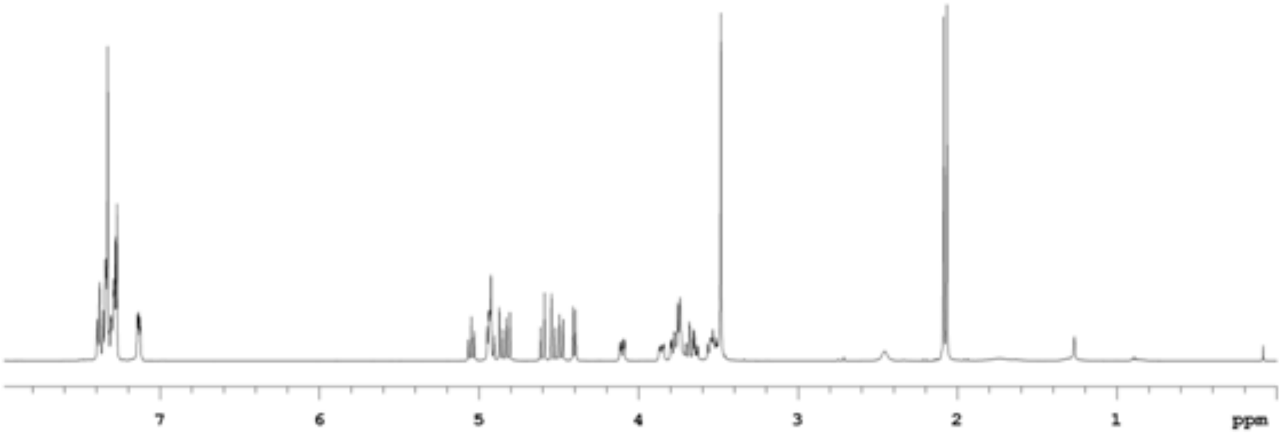

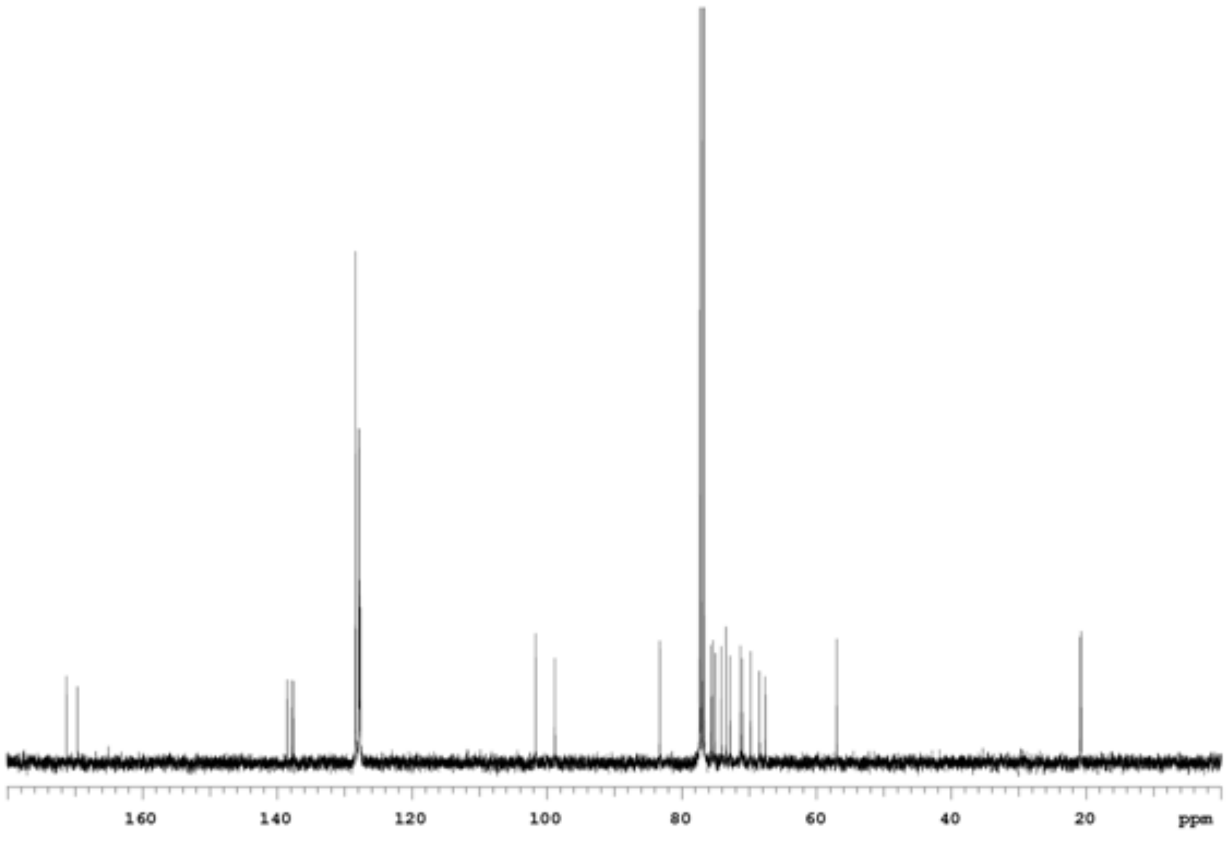

Sugar Silane 1a and Methyl-3,4-*O*-(2',3'-Dimethoxybutane-2',3'-Diyl)-Mannoside Silyl-Linked (4j)

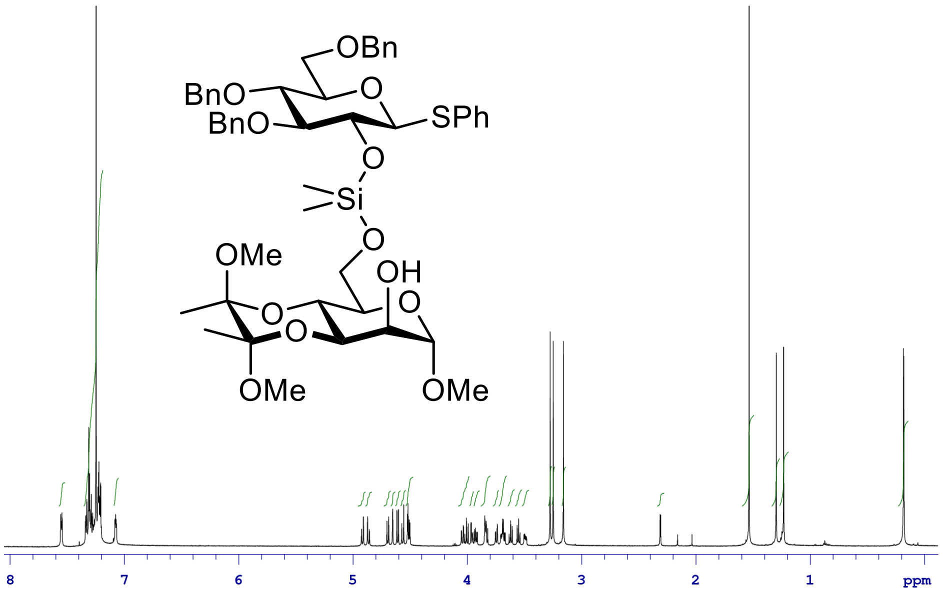

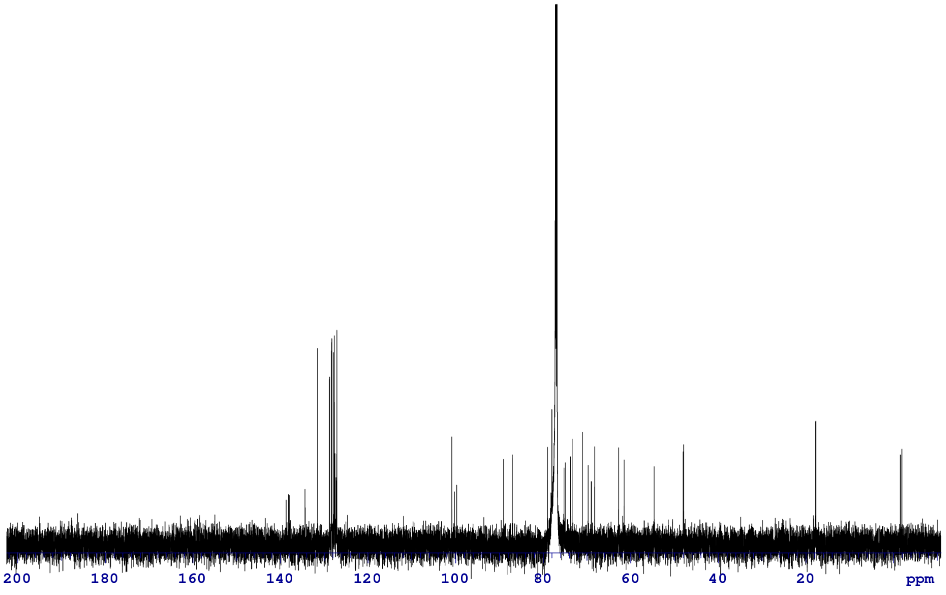

Sugar Silane 1a and Methyl-3,4-*O*-(2’,3’-Dimethoxybutane-2’,3’-Diyl)-Mannoside Glycoside (5j)

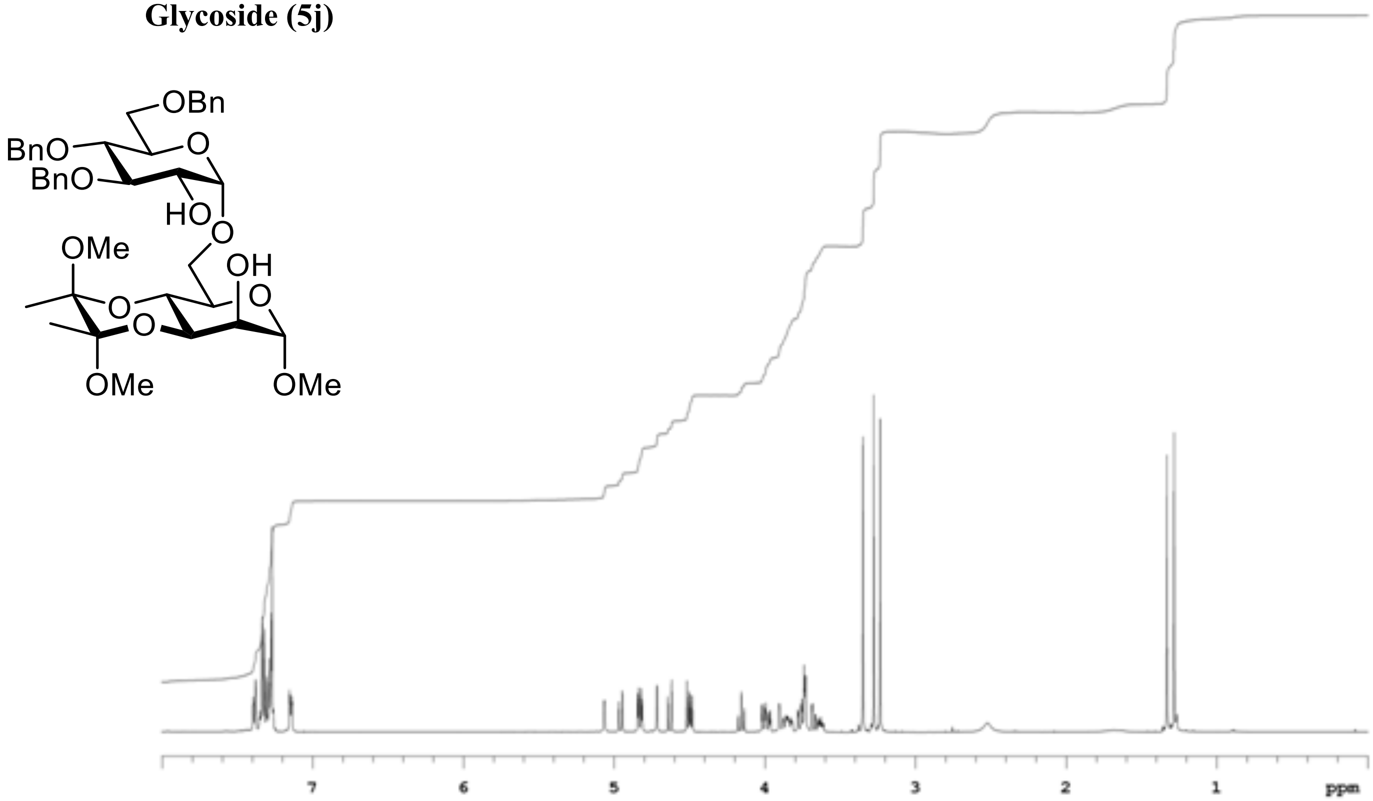

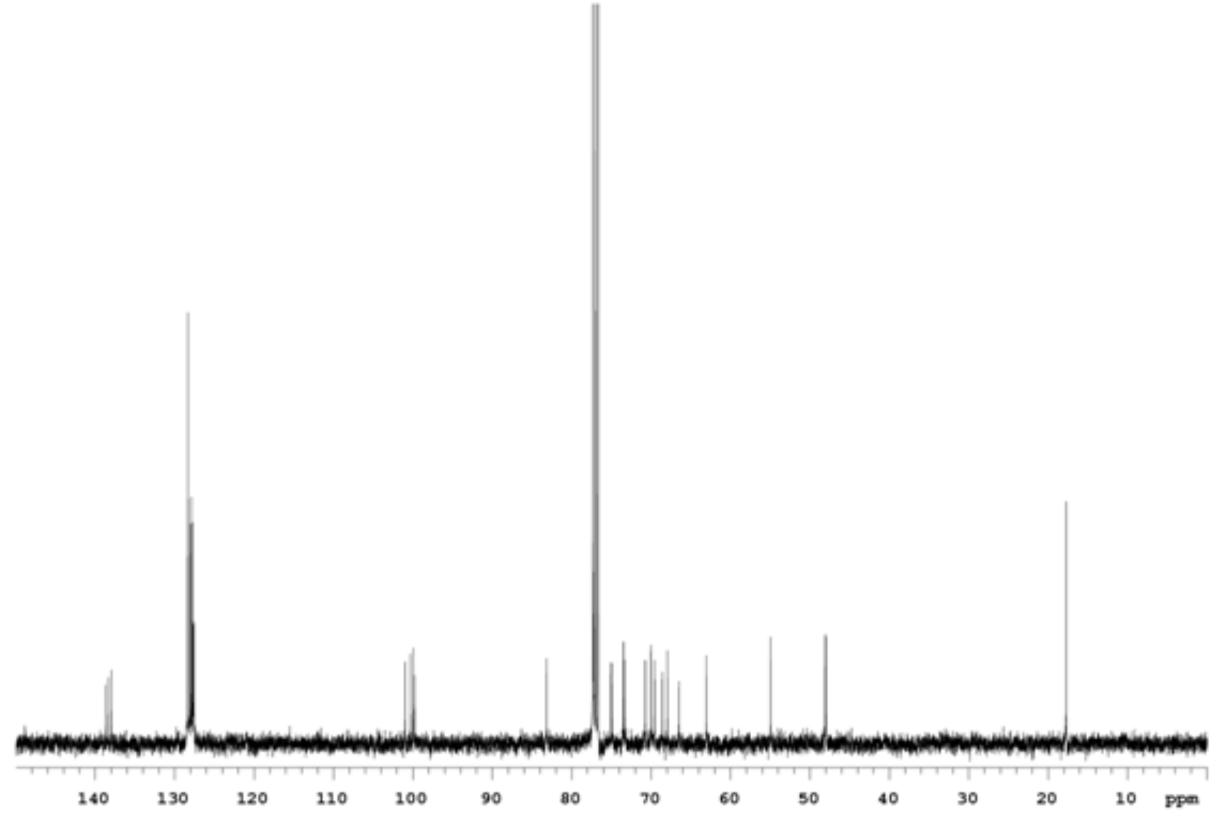

Phenyl 2-*O*-benzyl-6-*O*-*tert*-butyldimethylsilyl-3,4-*O*-(2',3'-dimethoxybutane-2',3'-diyl)-thio-β-D-glucopyranoside (14)

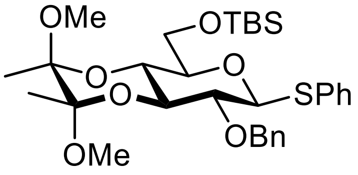

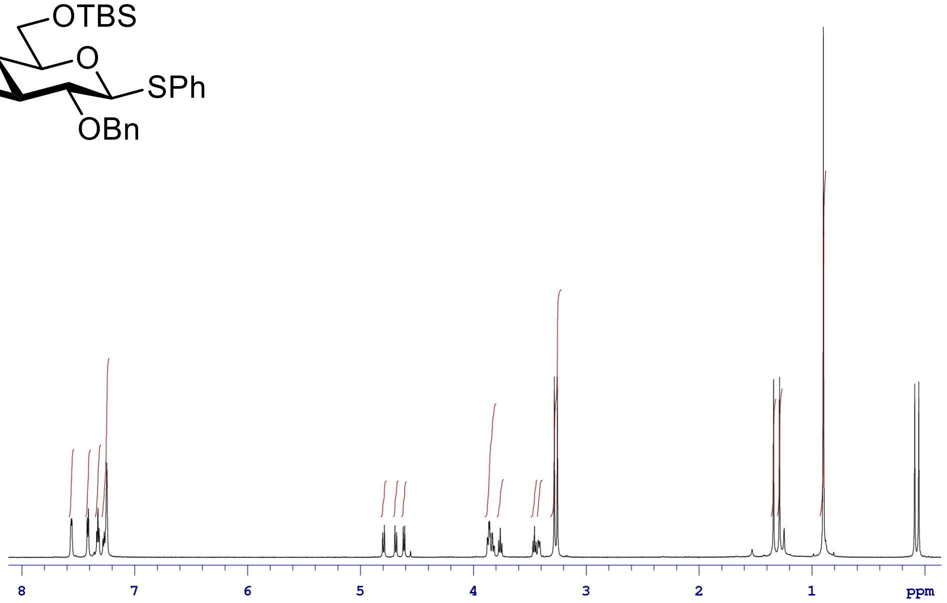

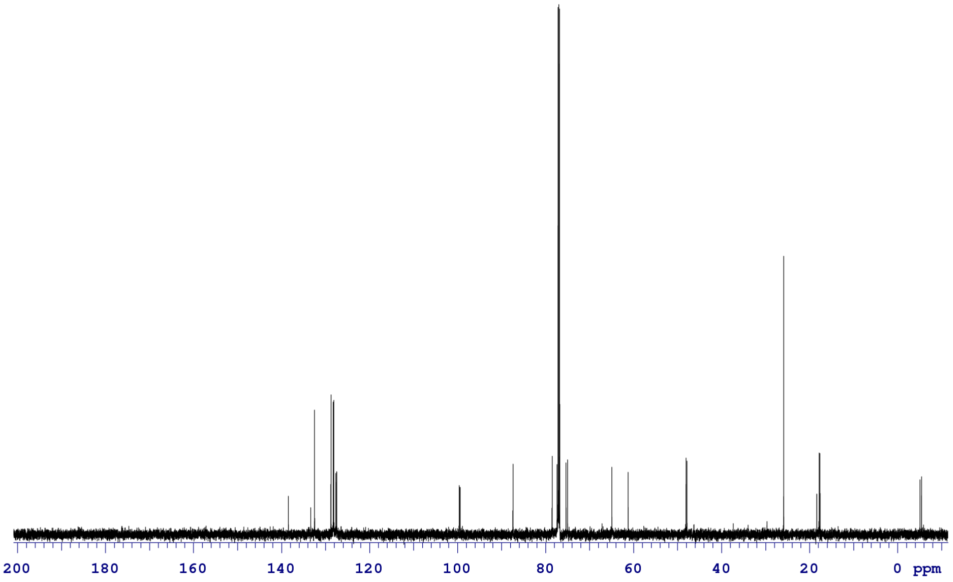

Phenyl 2-*O*-benzyl-3,4-*O*-(2’,3’-dimethoxybutane-2’,3’-diyl)-thio-β-D-glucopyranoside (7a)

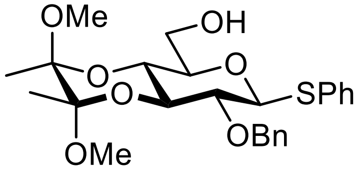

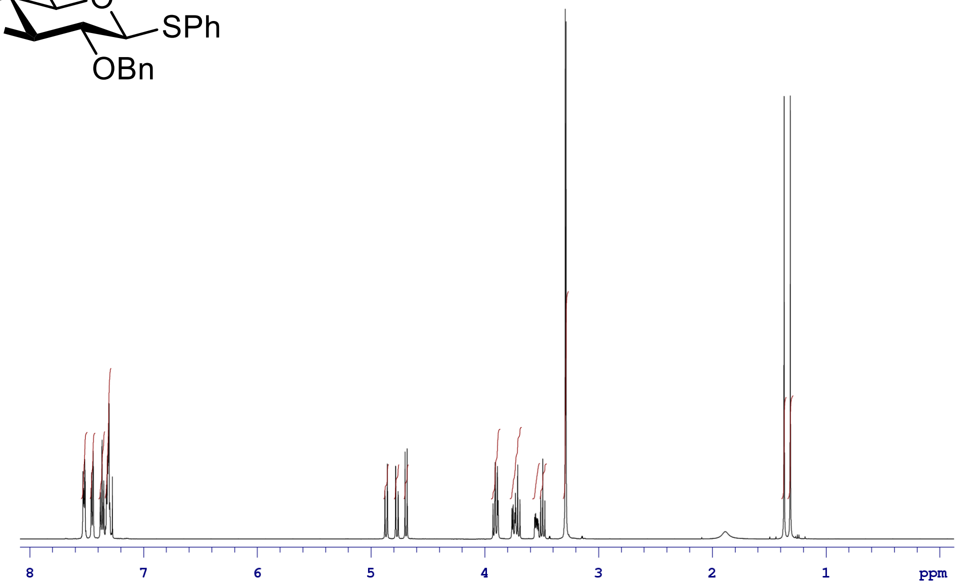

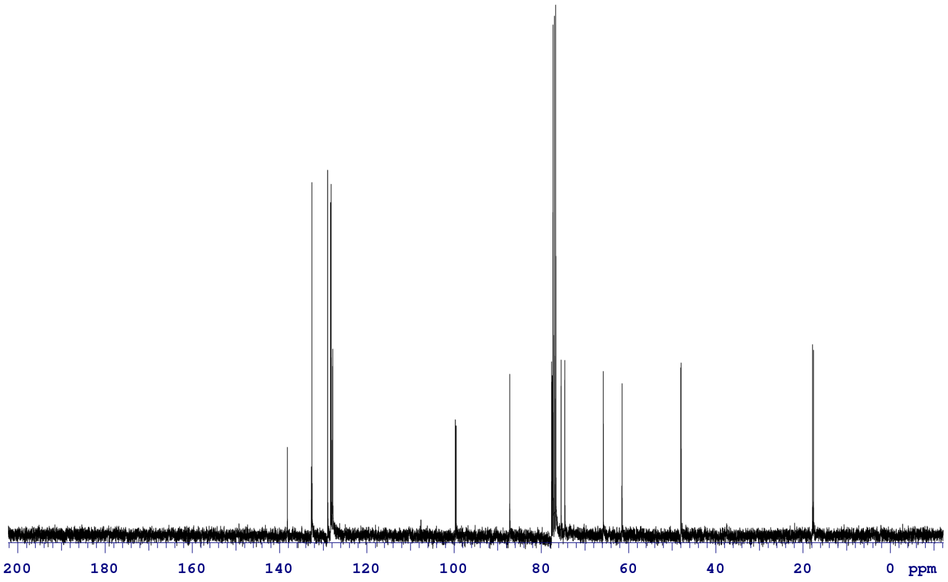

Sugar Silane (8a)

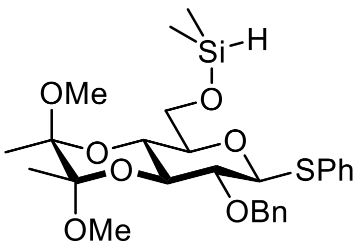

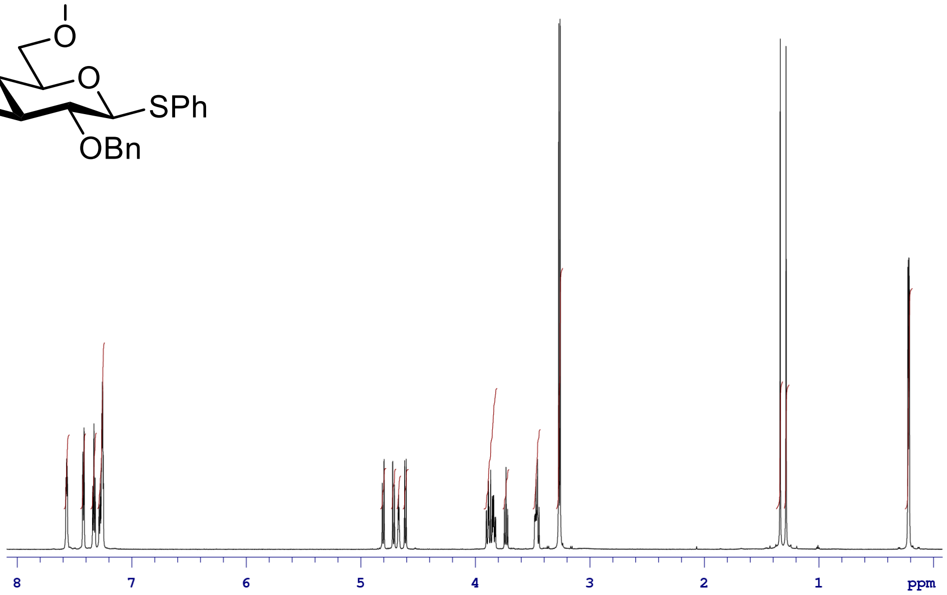

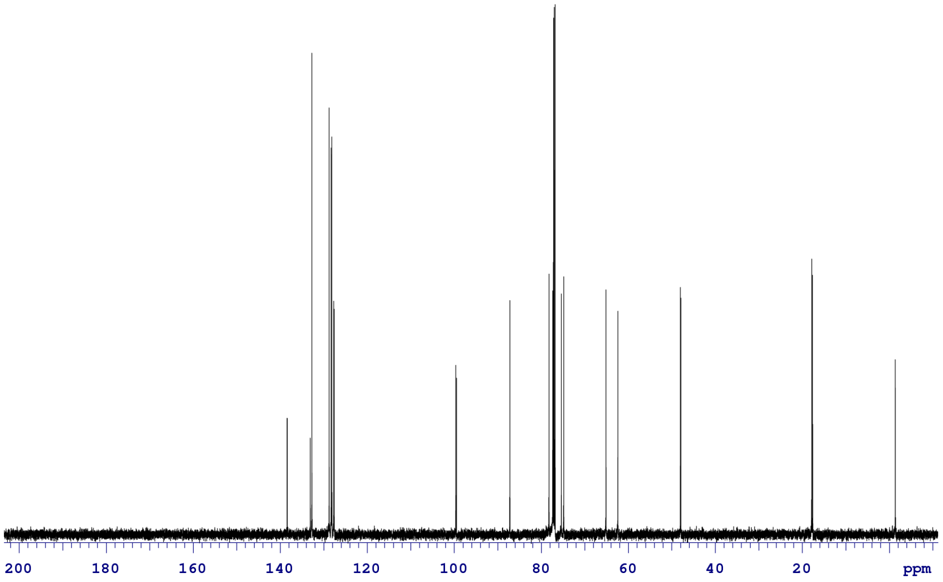

Phenyl 2-*O*-acetyl-6-*O*-*tert*-butyldimethylsilyl-3,4-*O*-(2’,3’-dimethoxybutane-2’,3’-diyl)-thio-β-D-glucopyranoside (15)

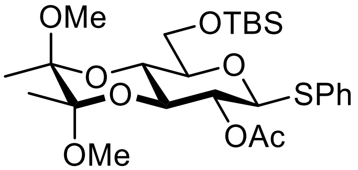

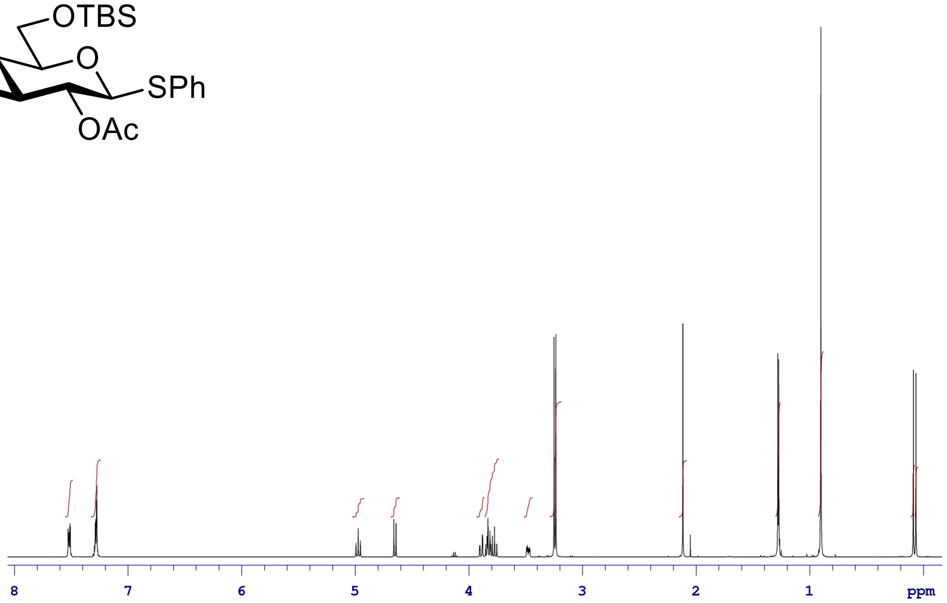

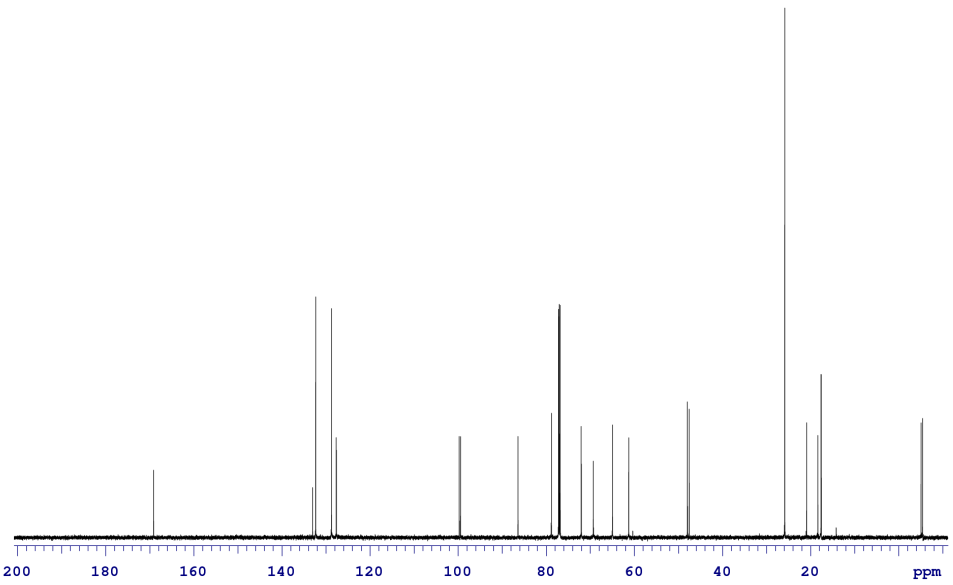

Phenyl 2-*O*-acetyl-3,4-*O*-(2’,3’-dimethoxybutane-2’,3’-diyl)-thio-β-D-glucopyranoside (7b)

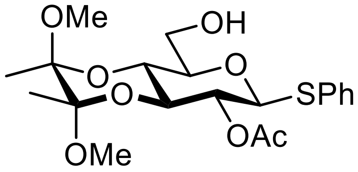

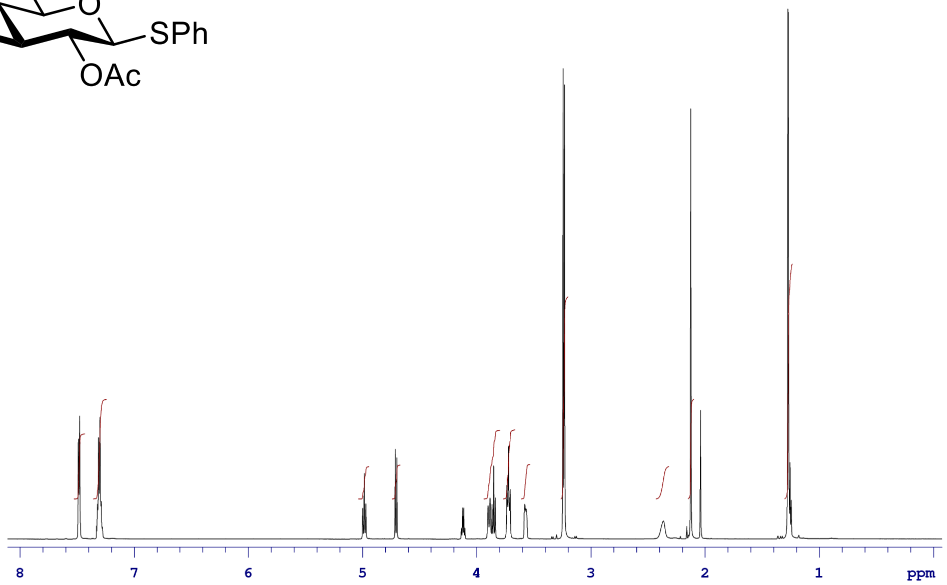

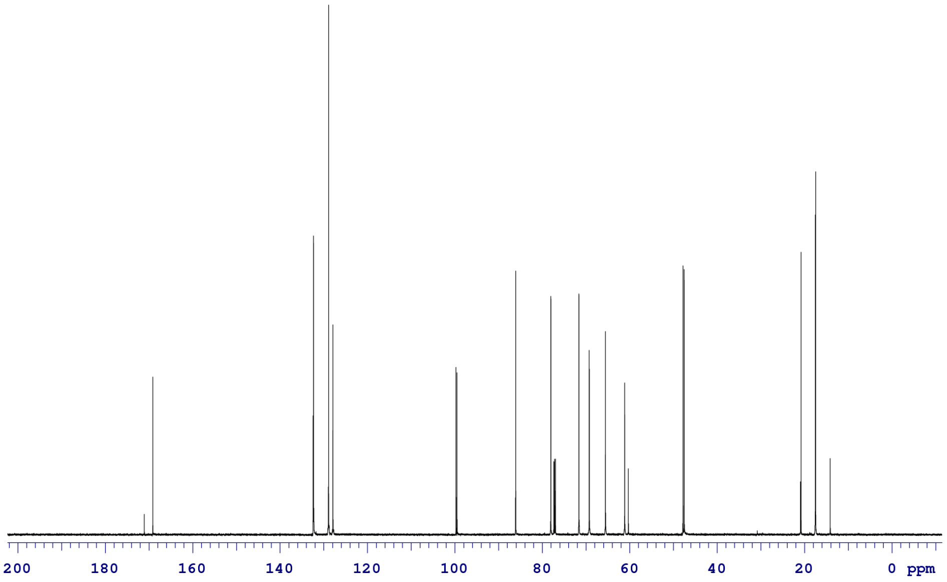

Sugar Silane (8b)

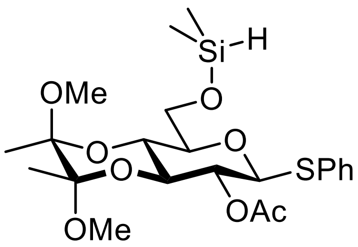

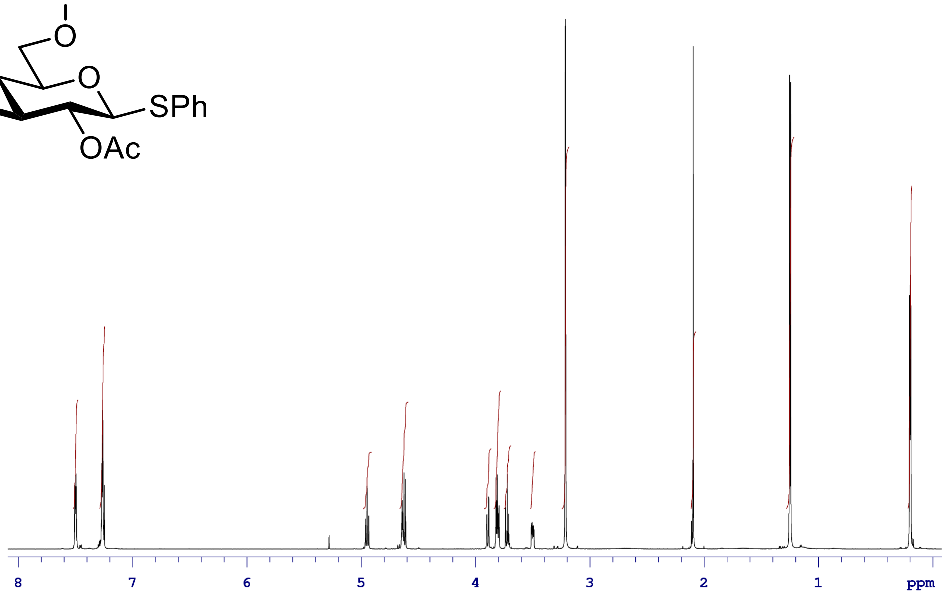

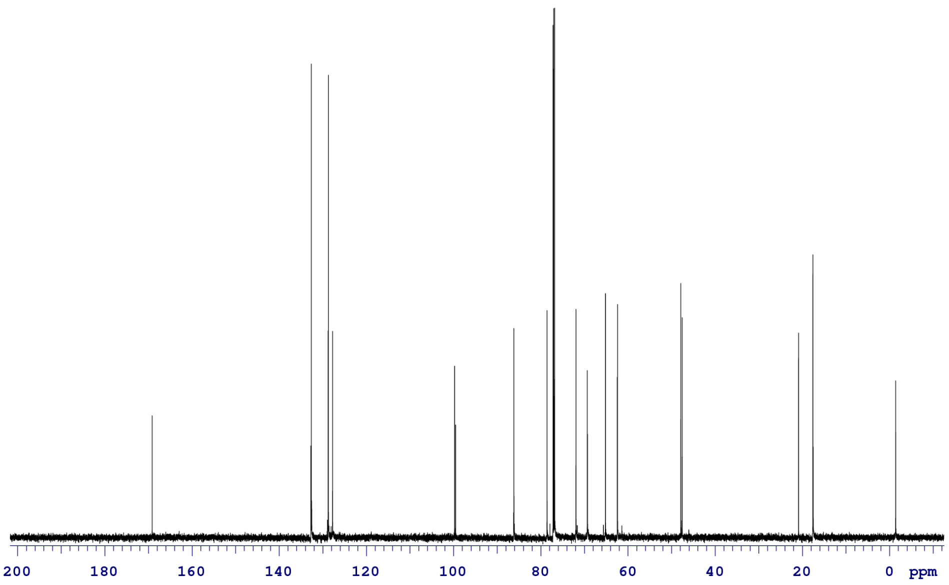

Phenyl 3,4,6-*O*-triacetyl-2-azido-2-deoxy-thio-β-D-glucopyranoside (16)

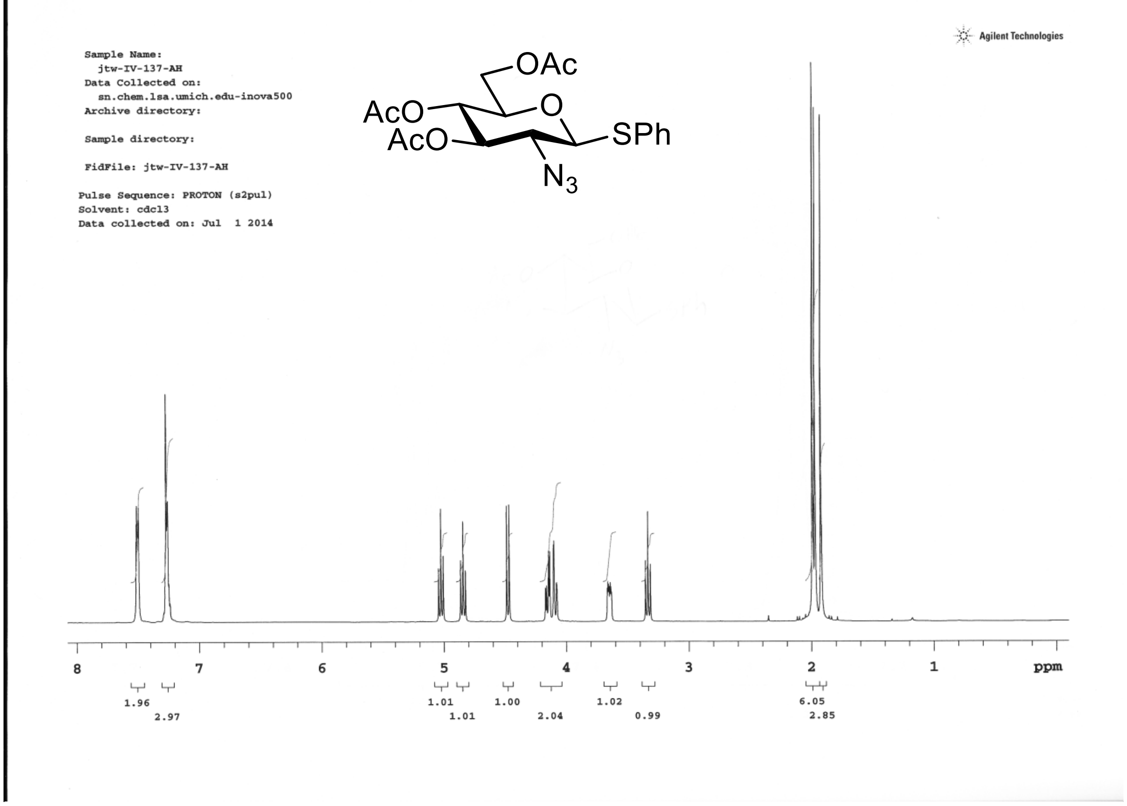

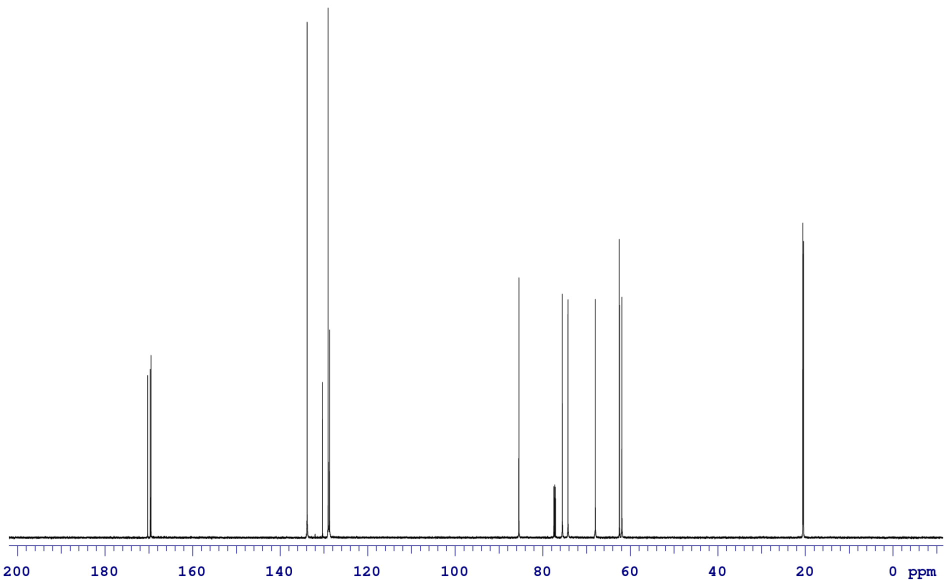

Phenyl 2-azido-2-deoxy-thio-β-D-glucopyranside (17)

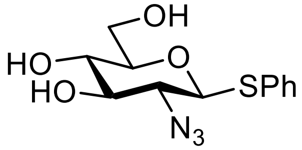

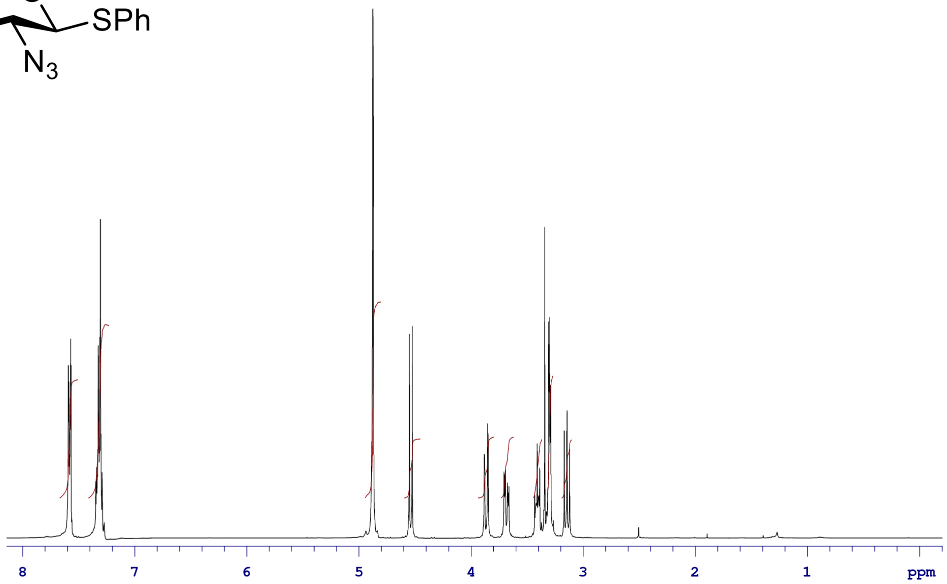

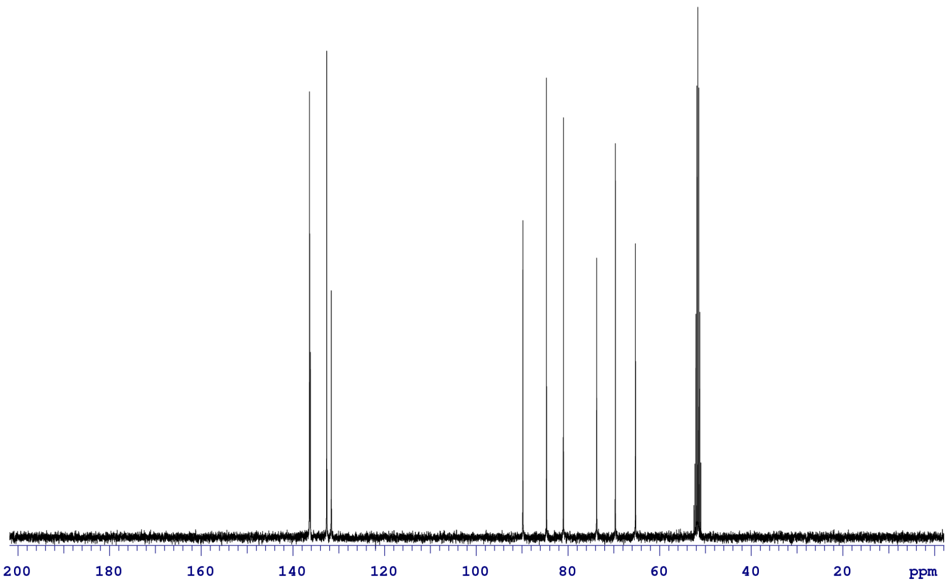

Phenyl 2-azido-2-deoxy-3,4-*O*-(2',3'-dimethoxybutane-2',3'-diyl)-thio-β-D-glucopyranoside (7c)

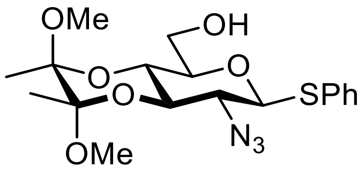

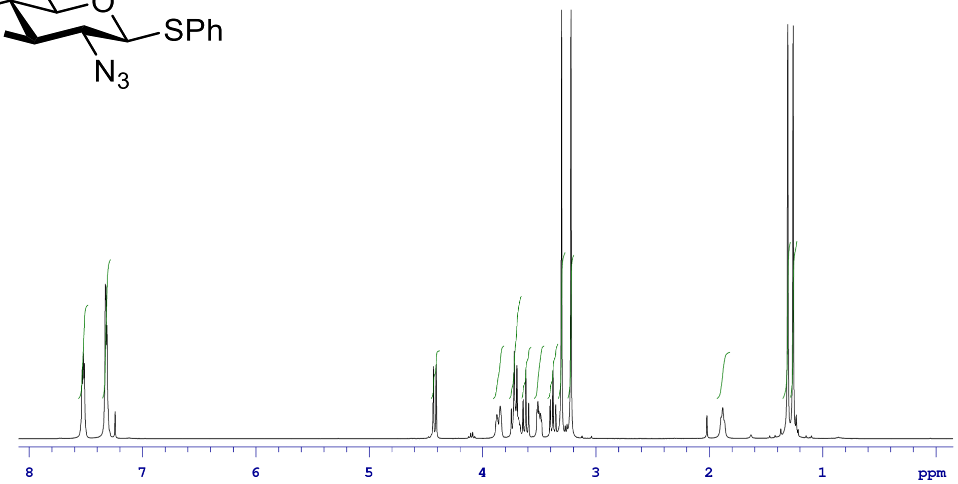

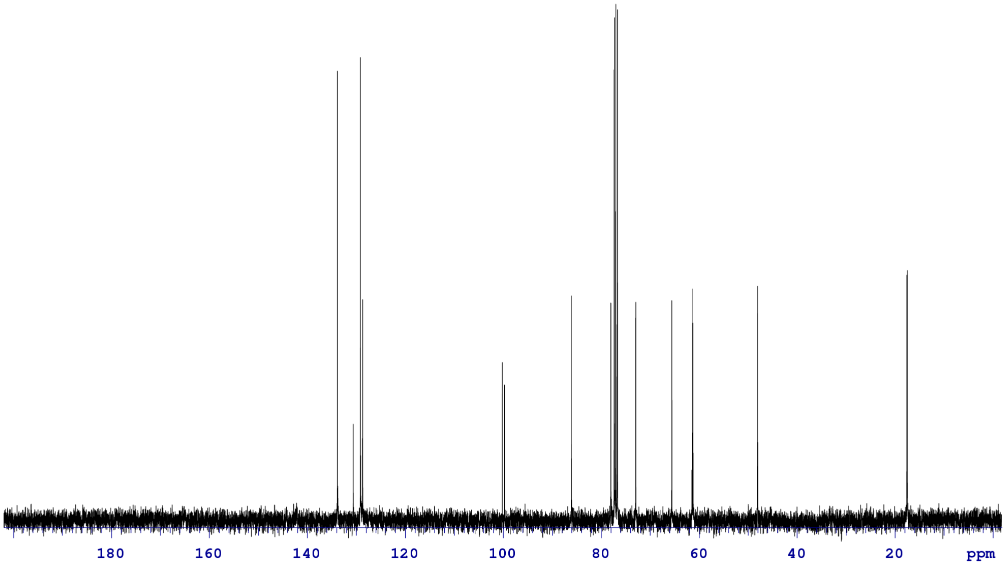

Sugar Silane (9)

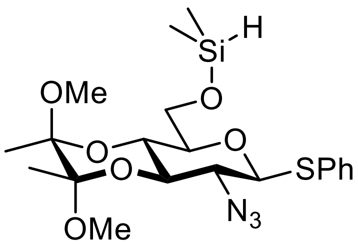

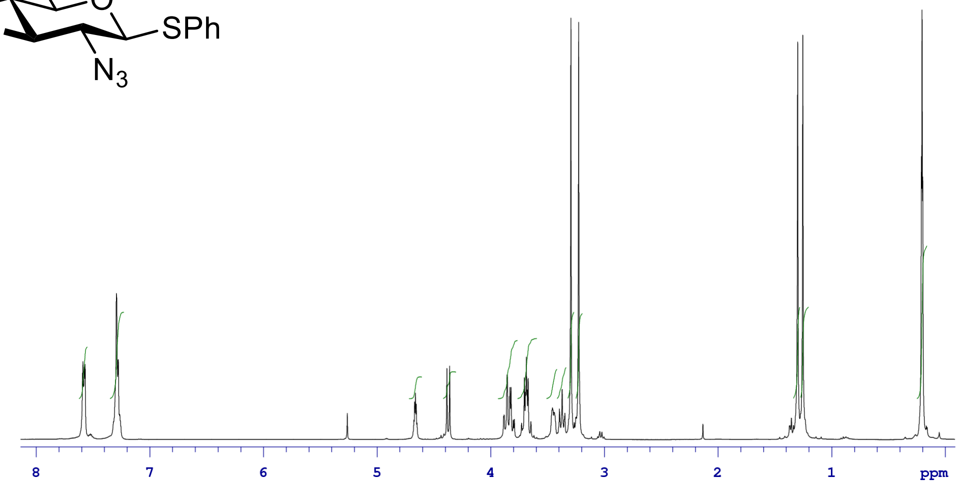

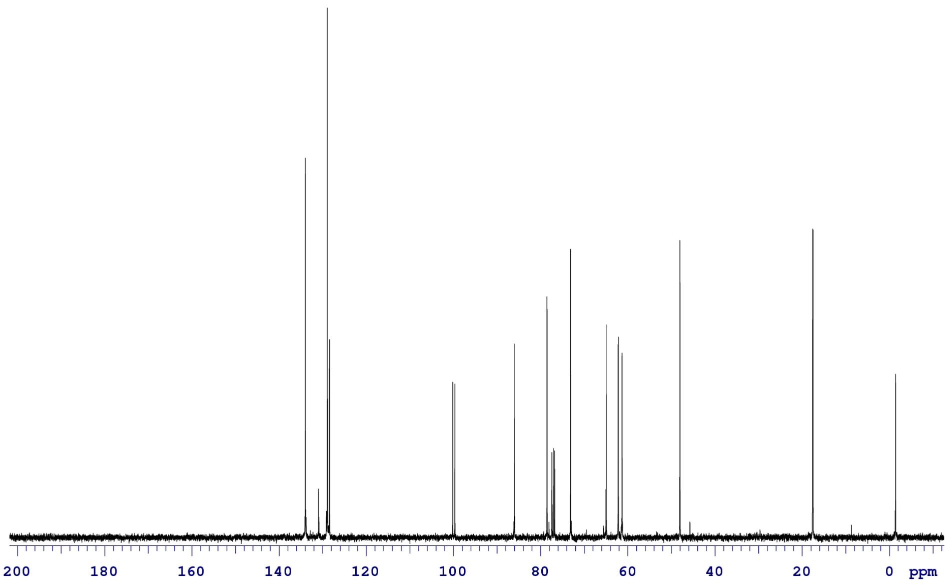

Phenyl 2-deoxy-3,4-*O*-(2’,3’-dimethoxybutane-2’,3’-diyl)-thio-α,β-D-glucopyranoside (7d)

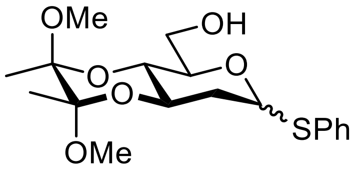

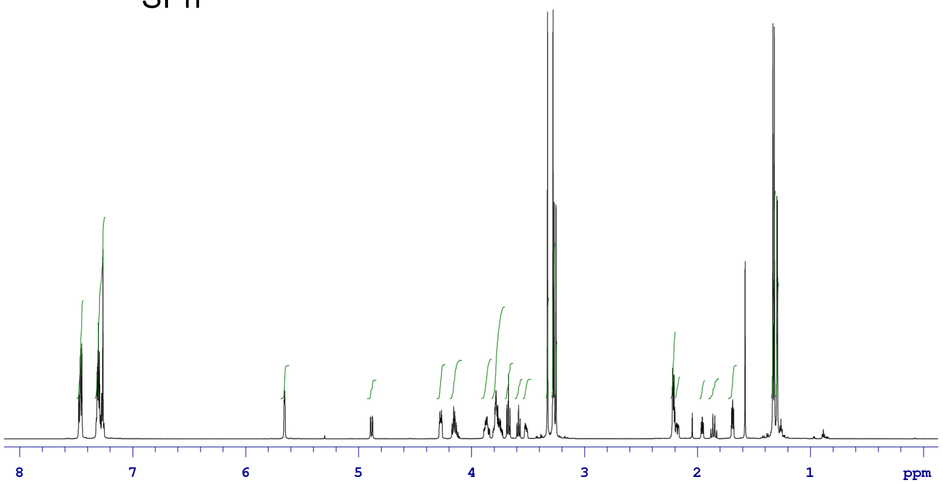

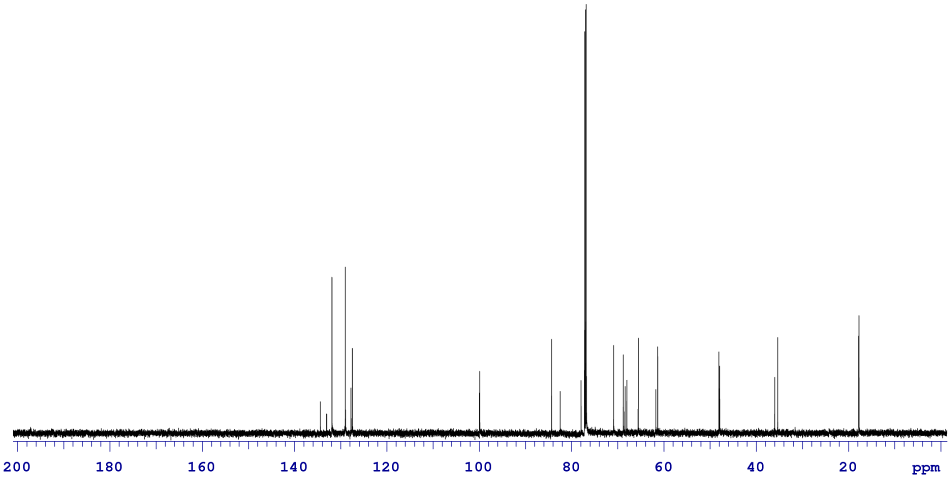

Sugar Silane (10)

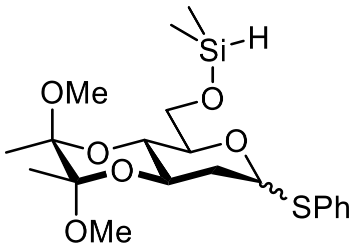

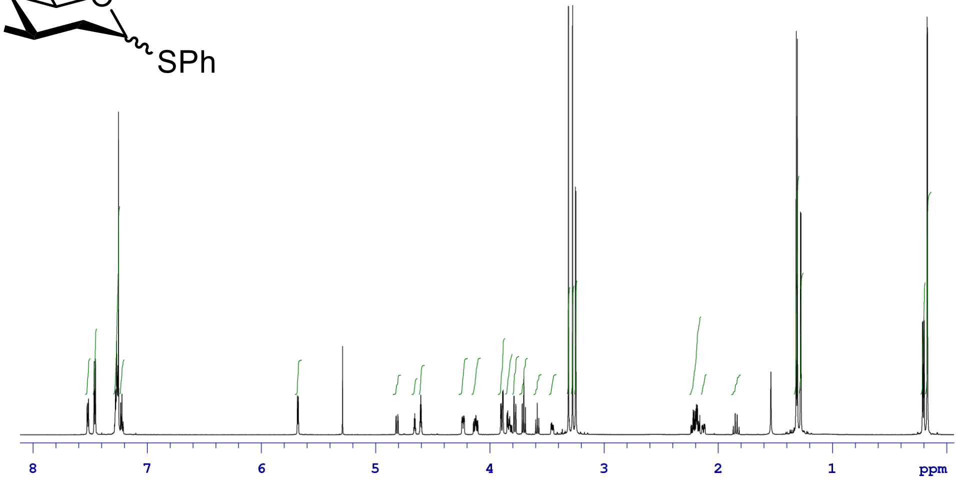

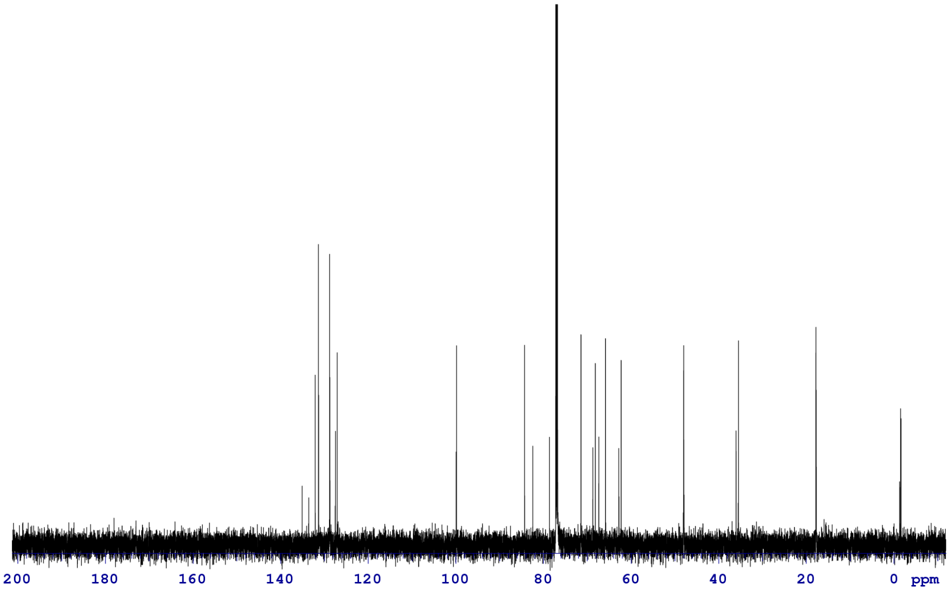

Sugar Silane 8a and Butanol Silyl-Linked (11a)

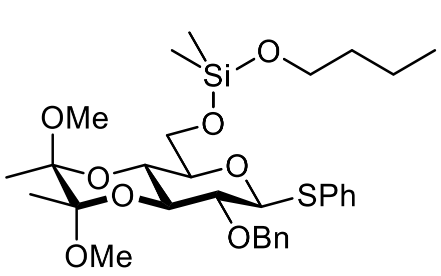

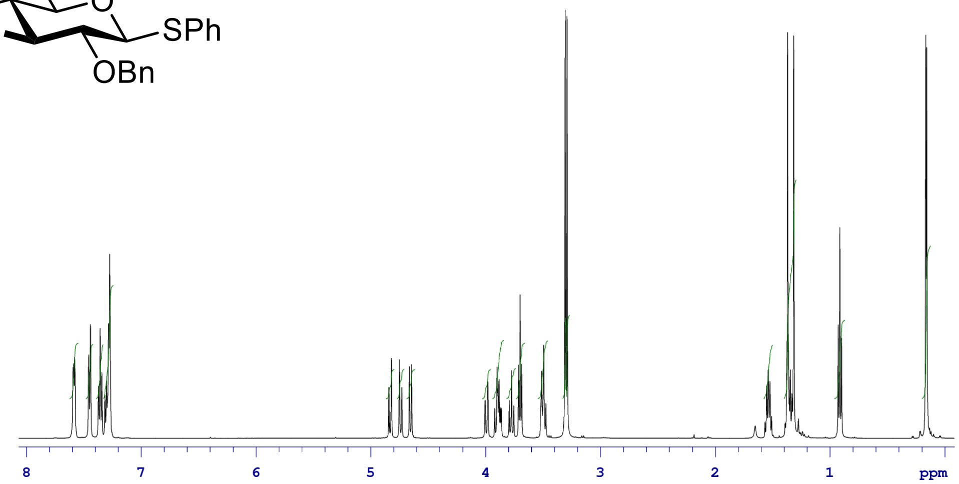

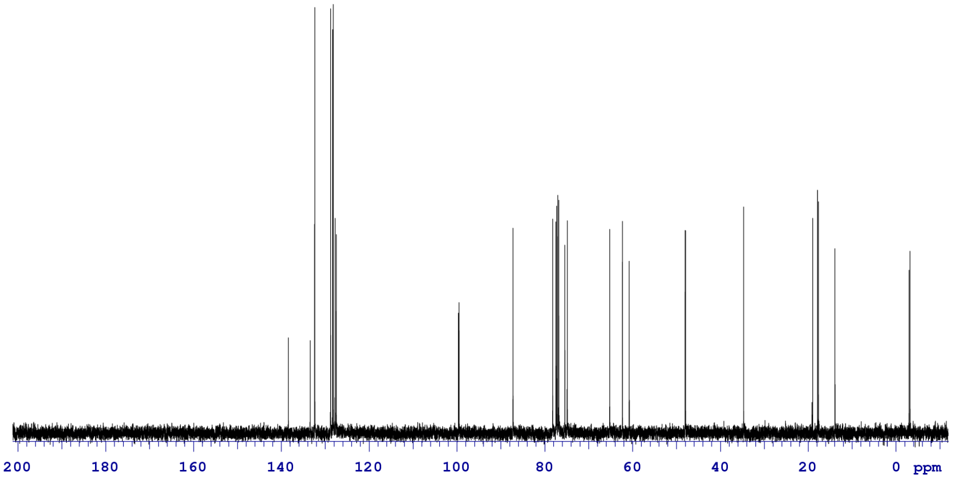

Sugar Silane 8a and Butanol Glycoside (12a)

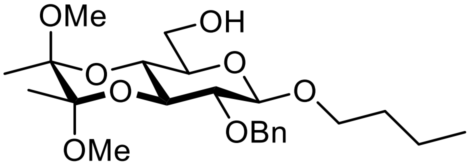

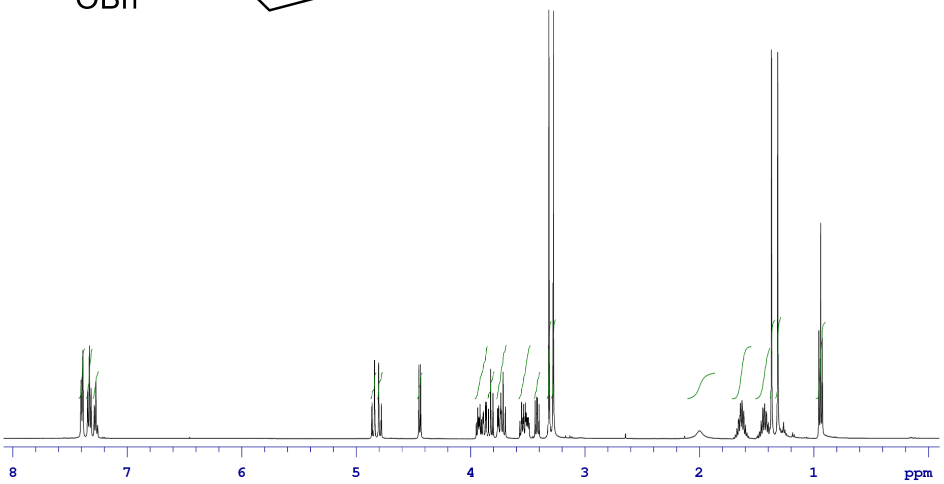

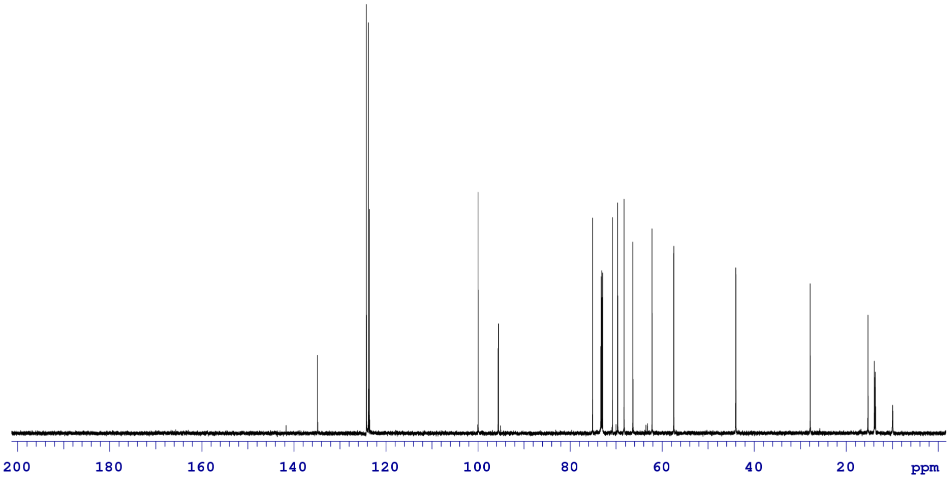

Sugar Silane 8a and Phenethyl Alcohol Silyl-Linked (11b)

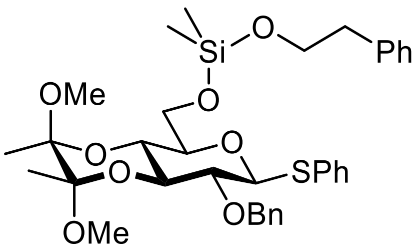

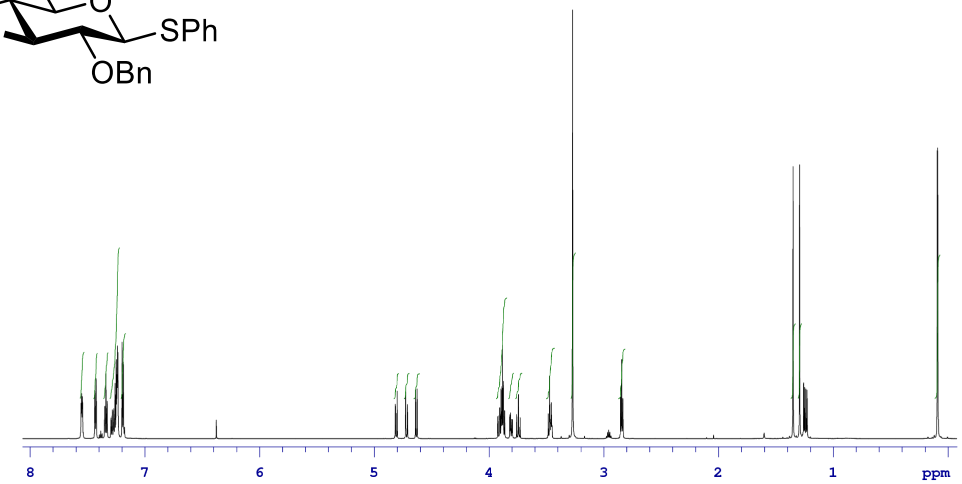

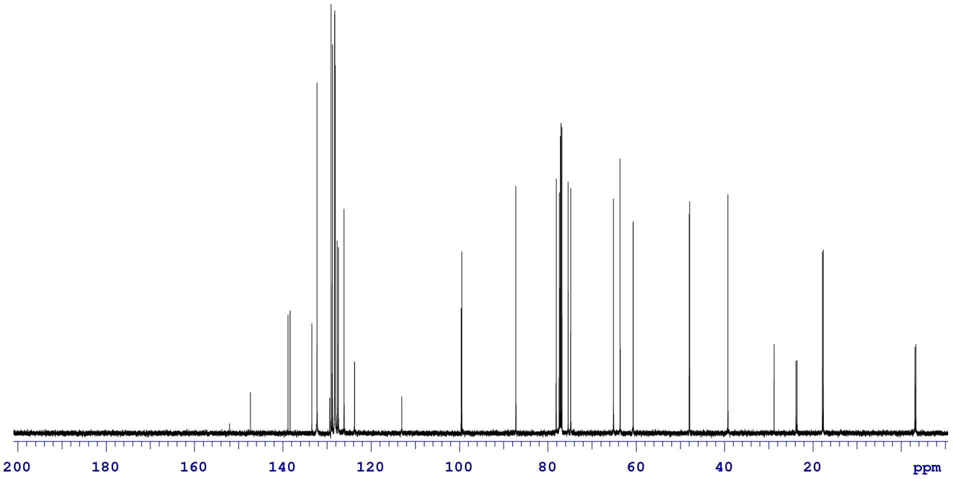

Sugar Silane 8a and Phenethyl Alcohol Glycoside (12b)

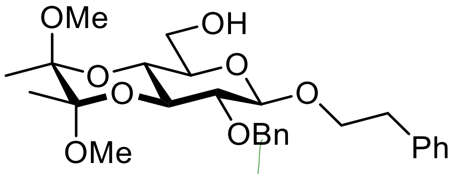

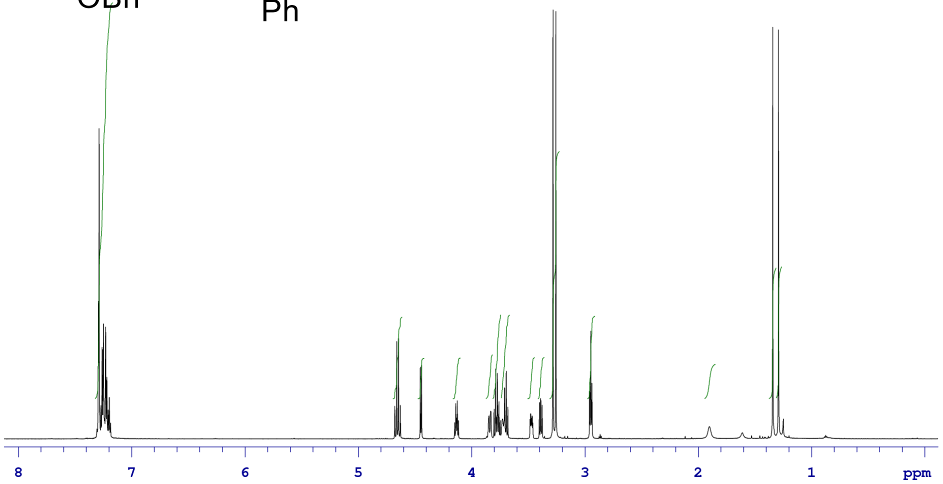

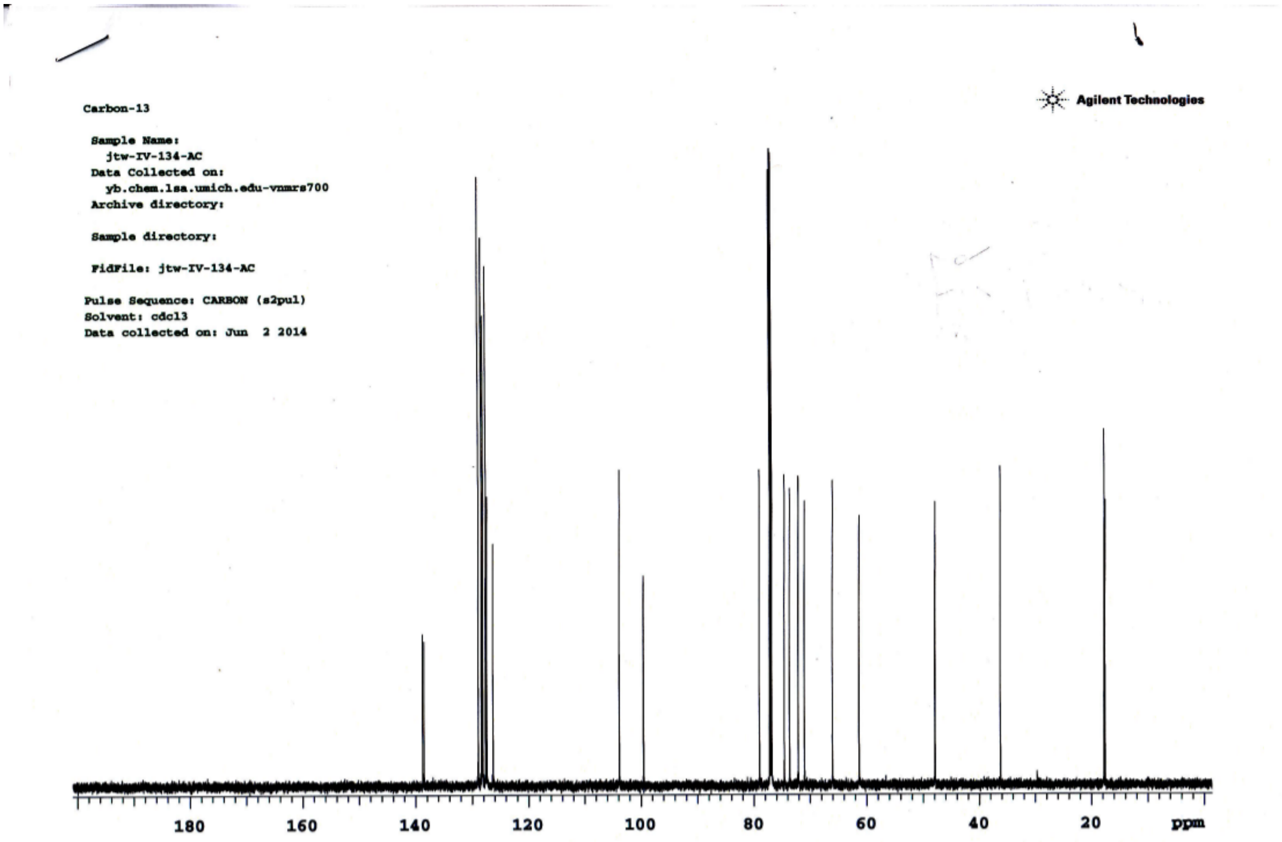

Sugar Silane 8a and Cyclohexanol Silyl-Linked (11c)

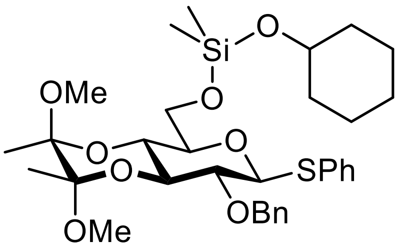

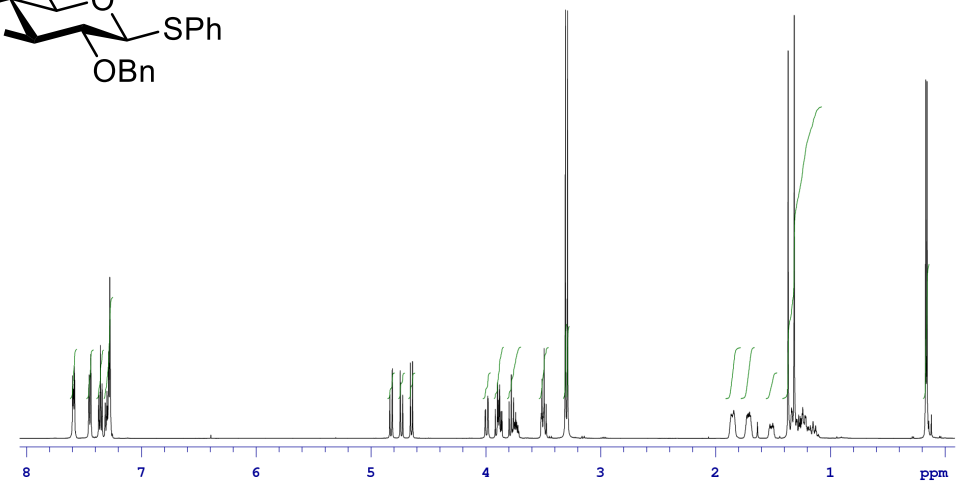

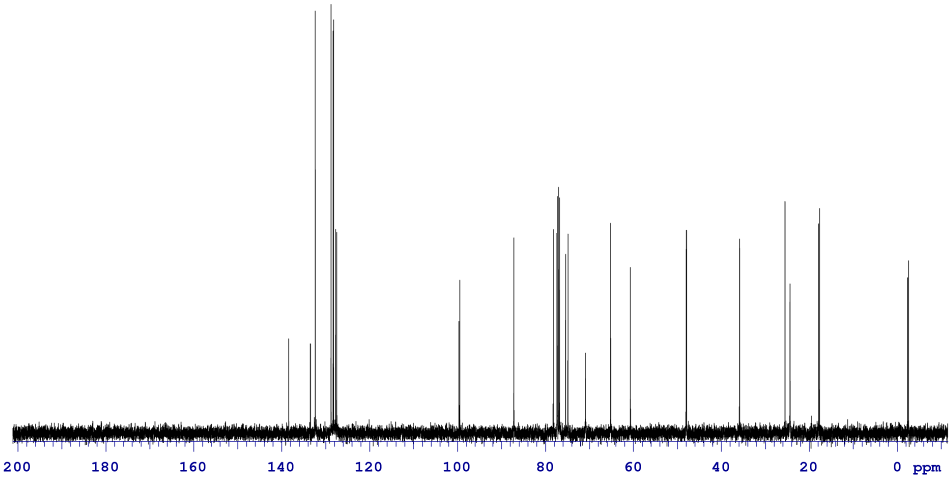

Sugar Silane 8a and Cyclohexanol Glycoside (12c)

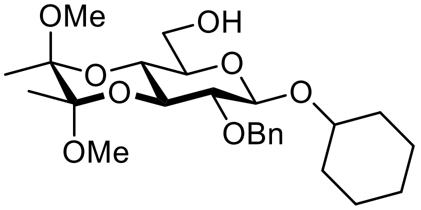

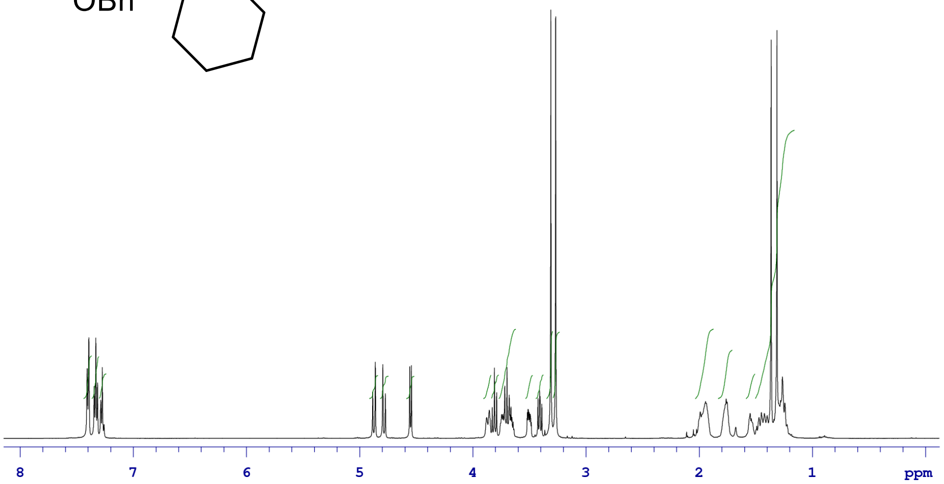

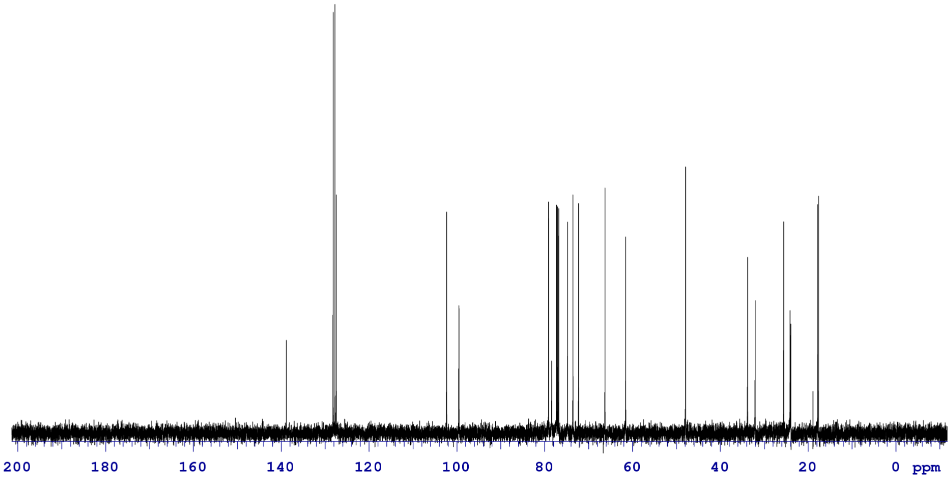

Sugar Silane 8a and Methyl 2,3,4-*O*-Tribenzyl-Glucopyranose Silyl-Linked (11d)

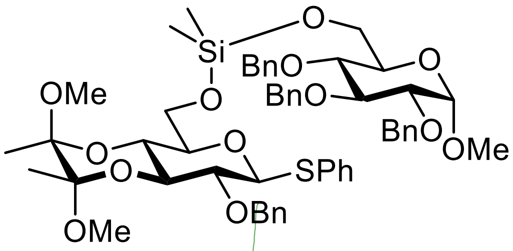

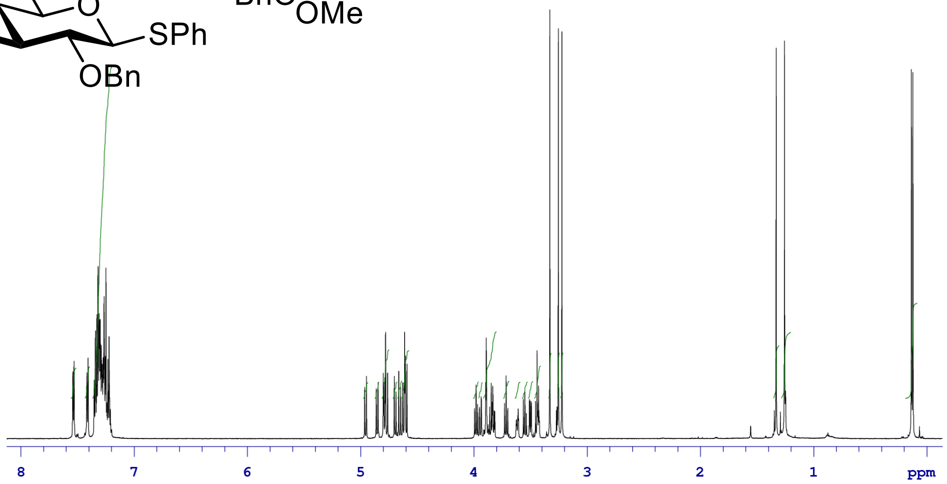

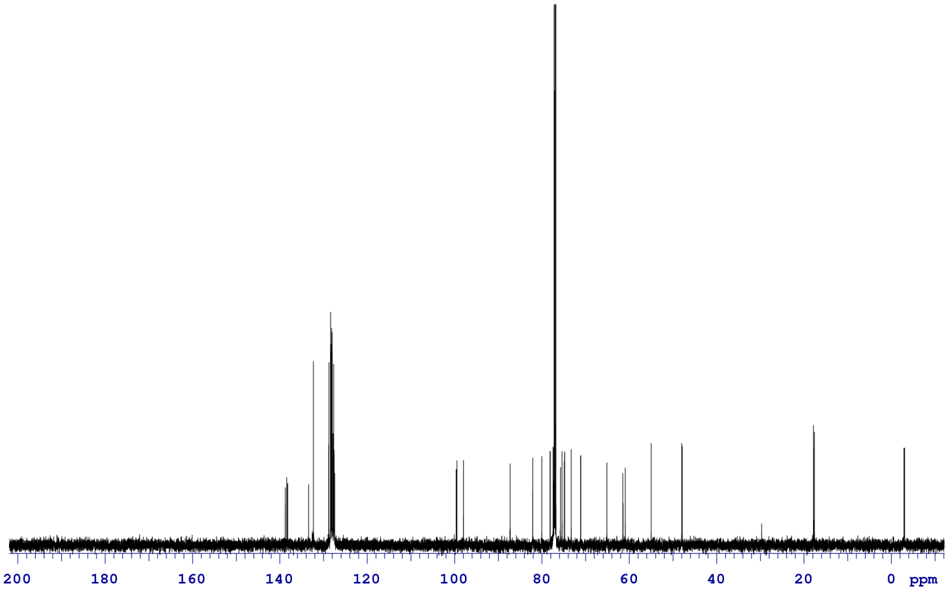

Sugar Silane 8a and Methyl 2,3,4-*O*-Tribenzyl-Glucopyranose Glycoside (12d)

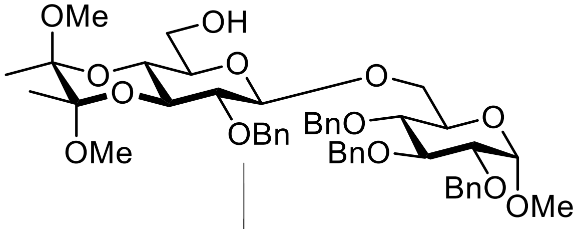

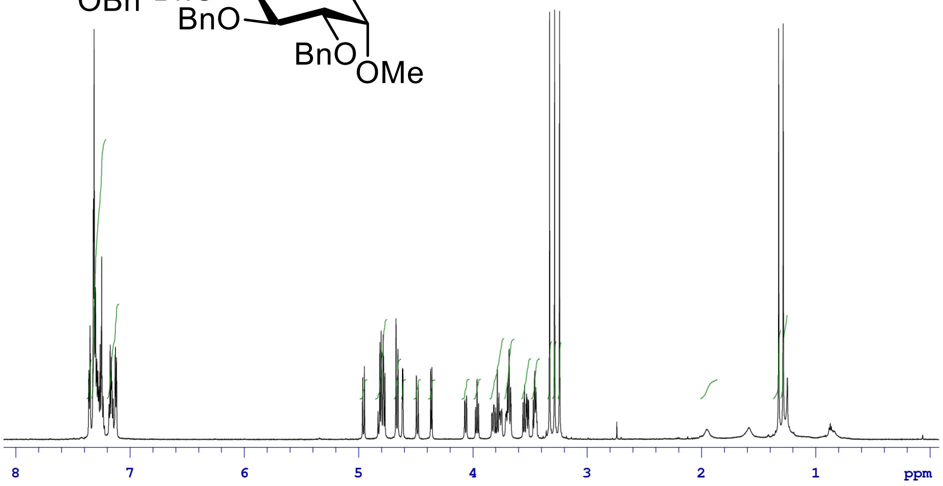

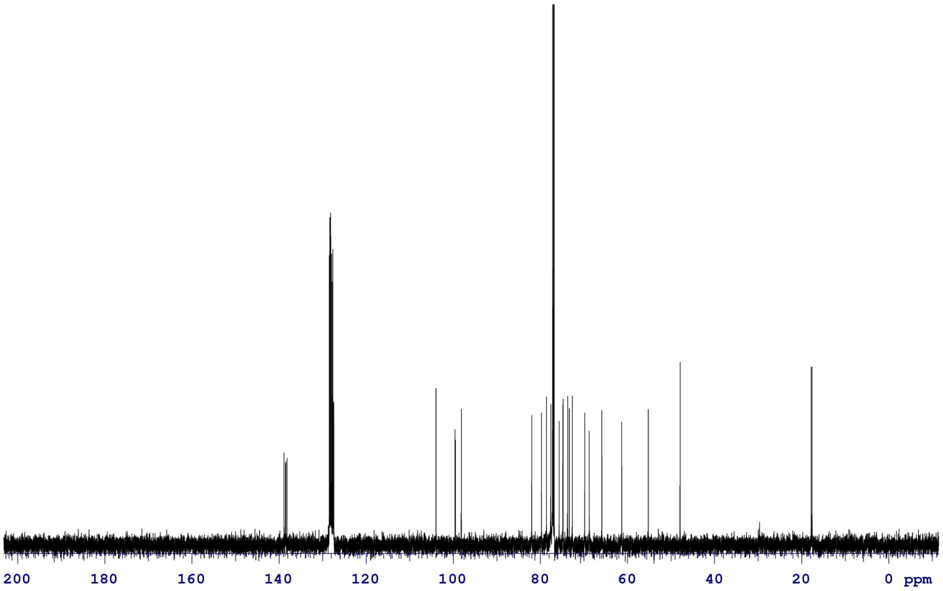

Sugar Silane 1b and 1,2:3,4-*O*-Diisopropylidene-D-Galactopyranose Glycoside (12e)

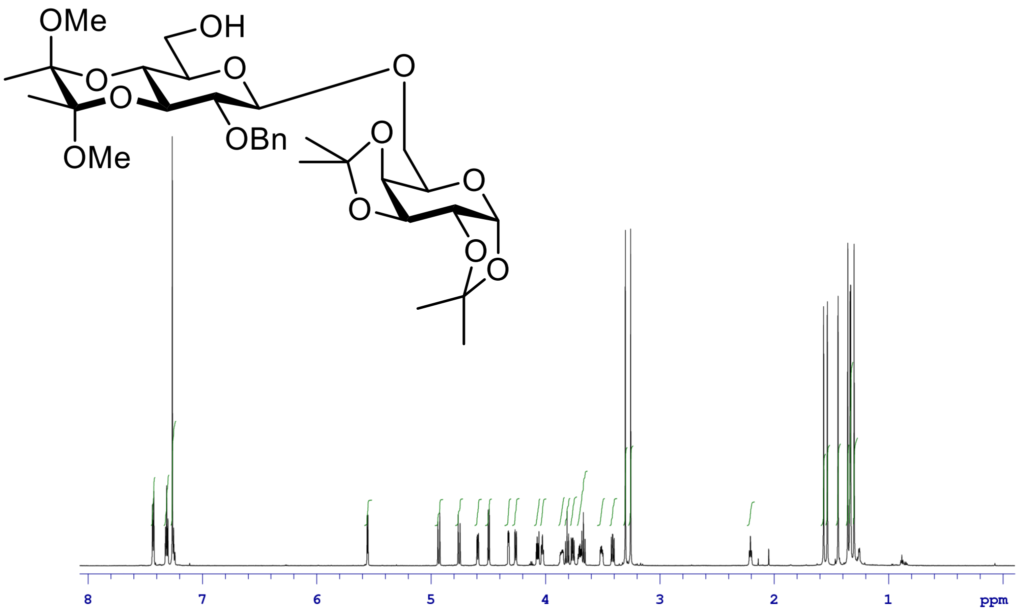

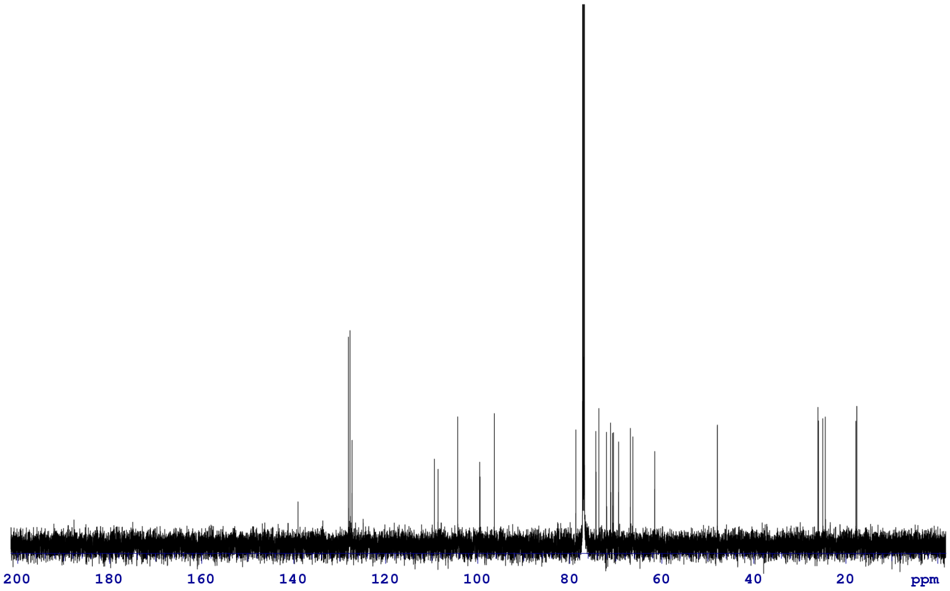

Sugar Silane 8a and Ketosteroid Silyl-Linked (11f)

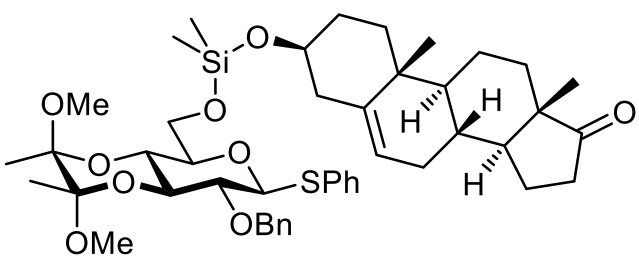

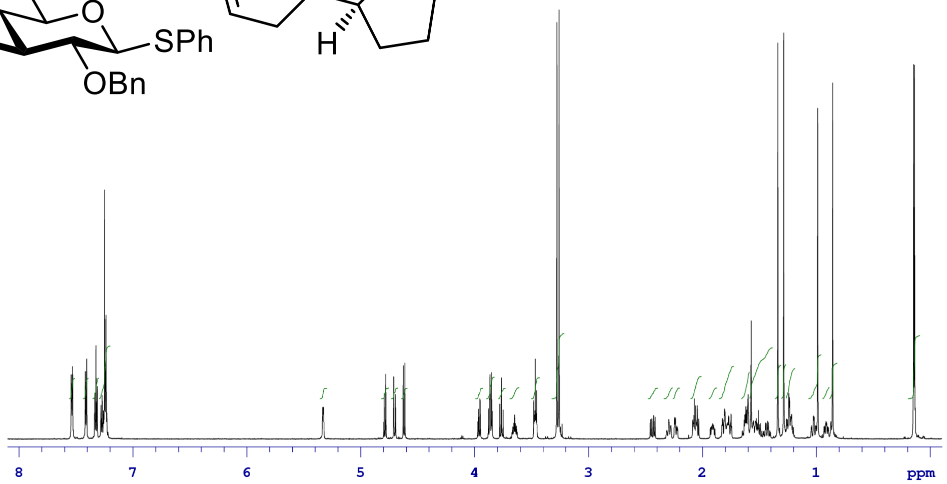

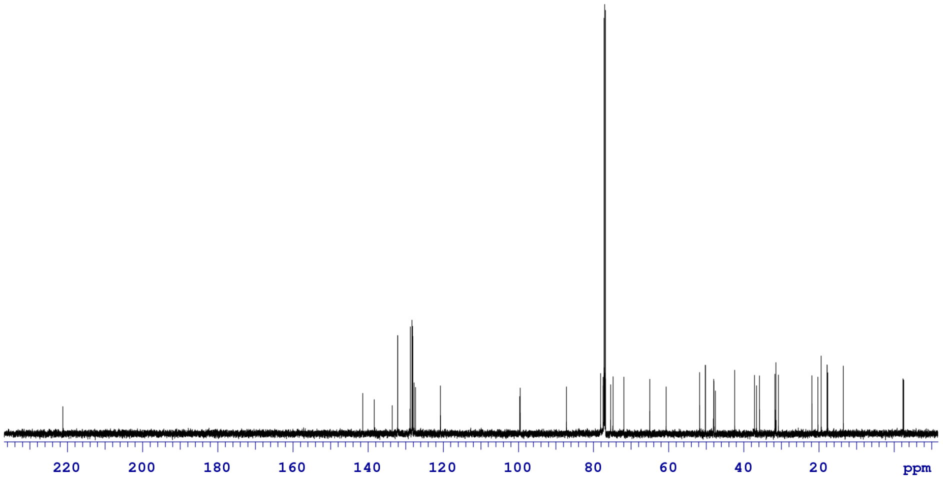

Sugar Silane 8a and Ketosteroid Glycoside (12f)

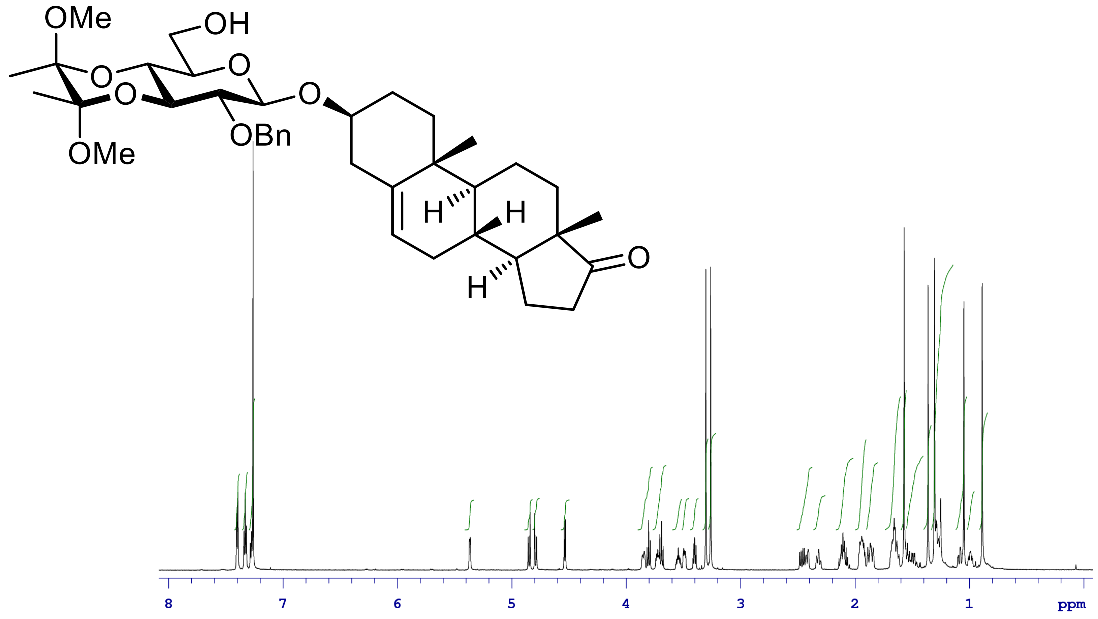

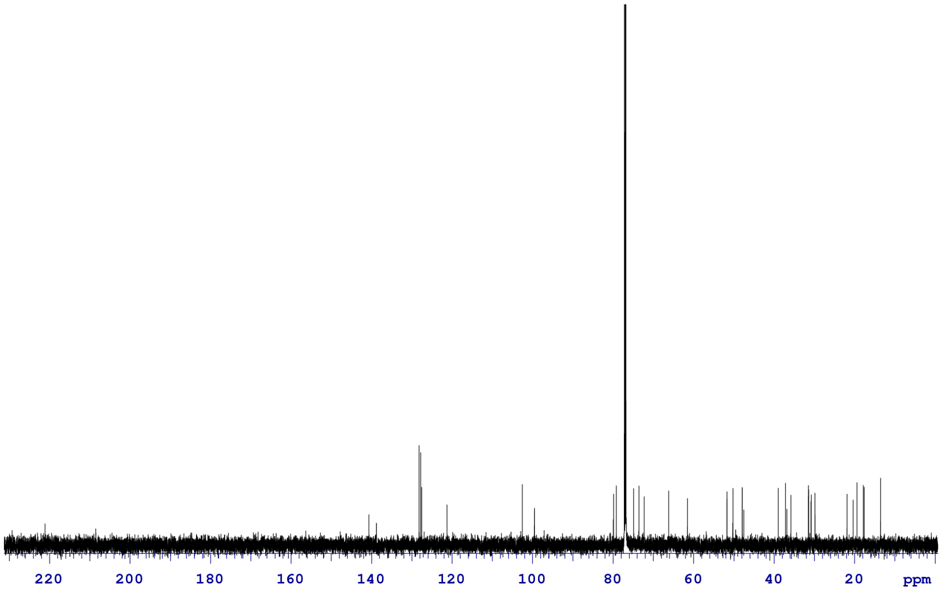

Sugar Silane 8b and Butanol Silyl-Linked (11g)

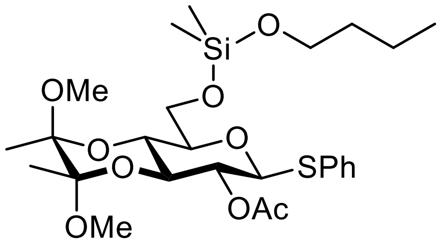

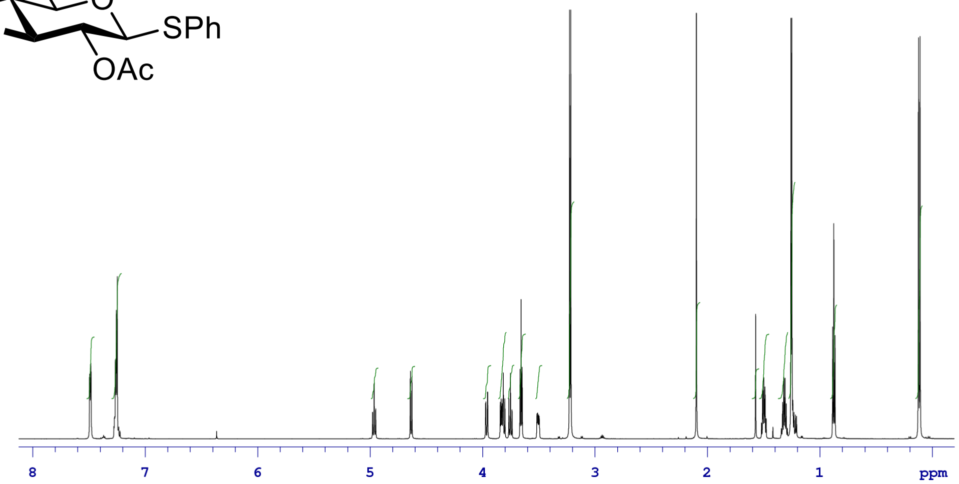

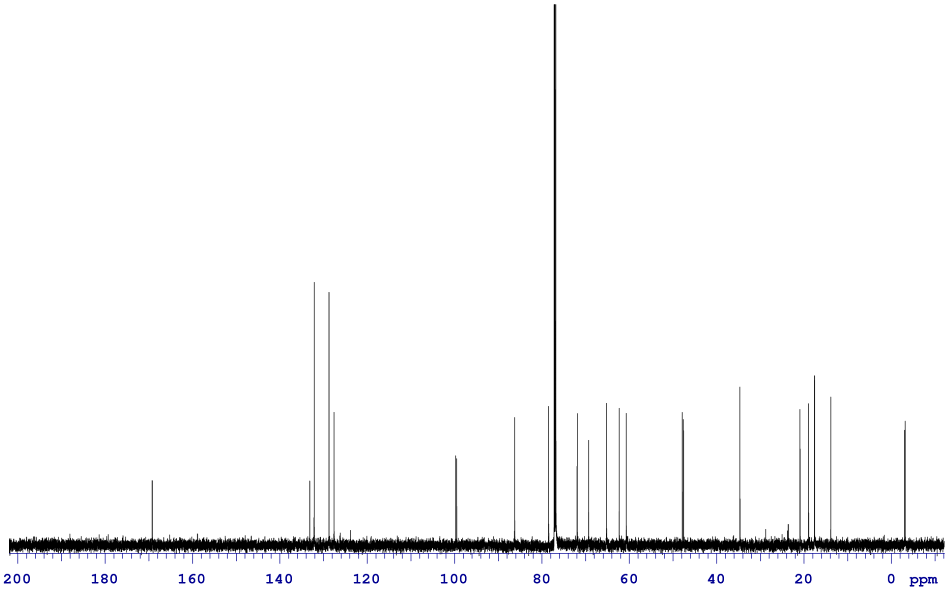

Sugar Silane 8b and Butanol Glycoside (12g)

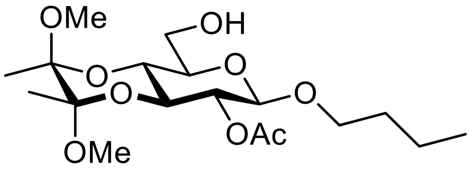

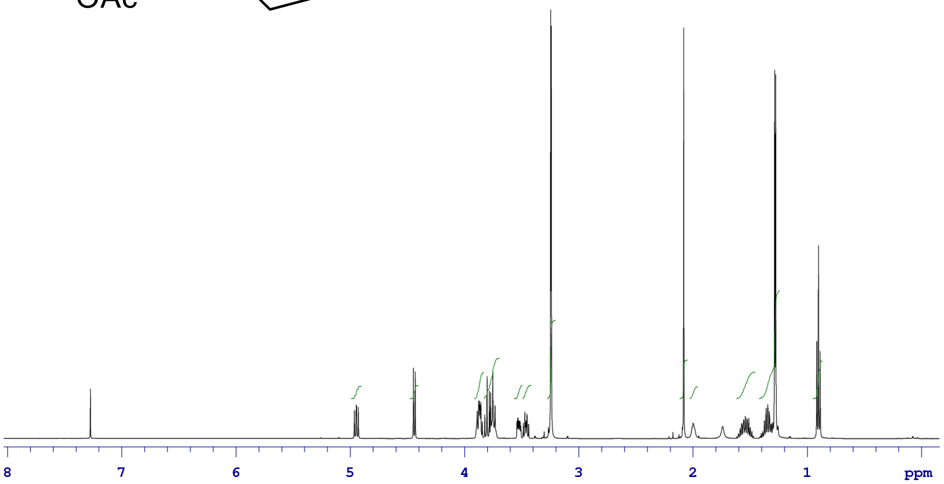

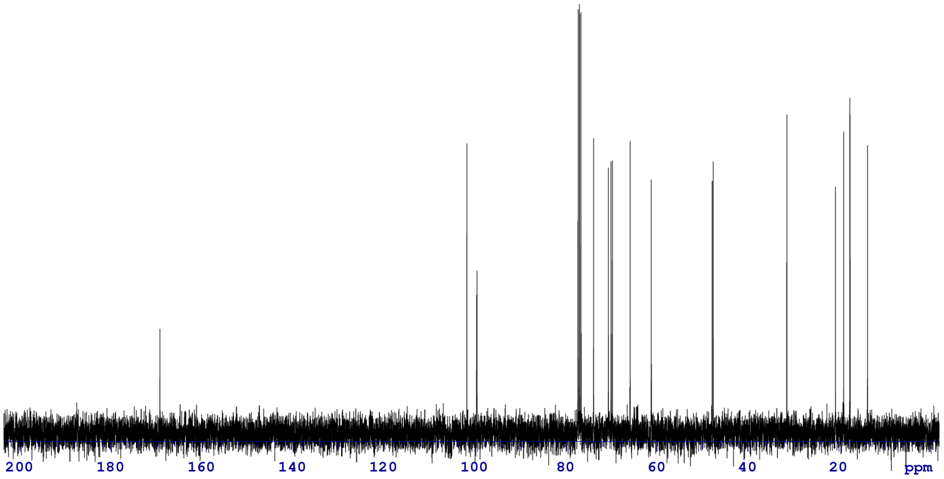

Sugar Silane 9 and Butanol Silyl-Linked (11h)

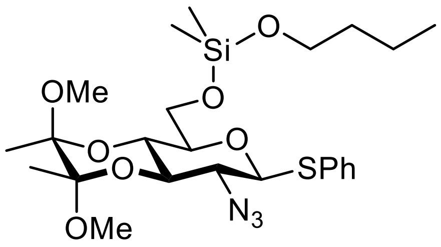

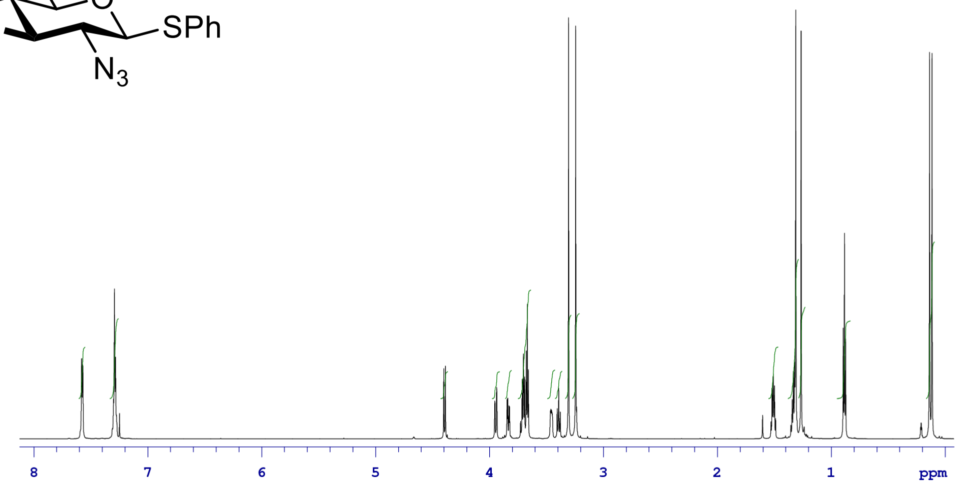

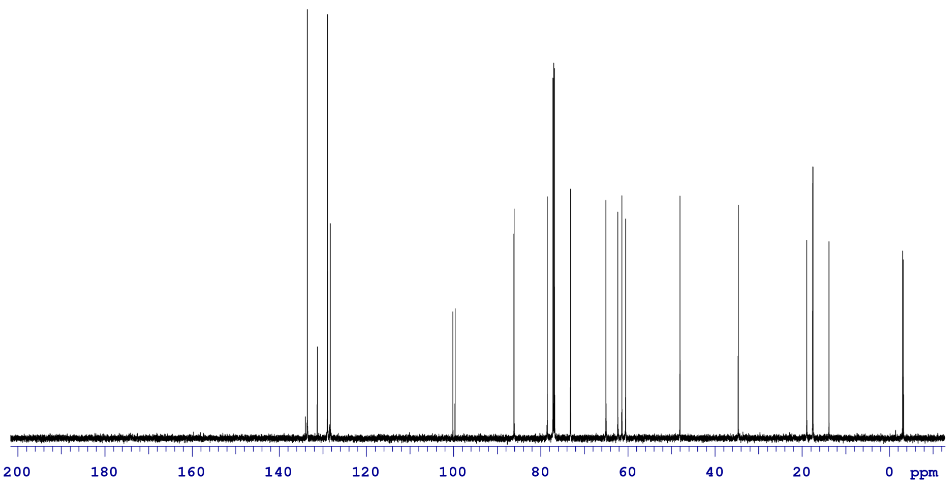

Sugar Silane 9 and Butanol Glycoside (12h)

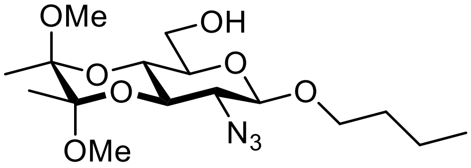

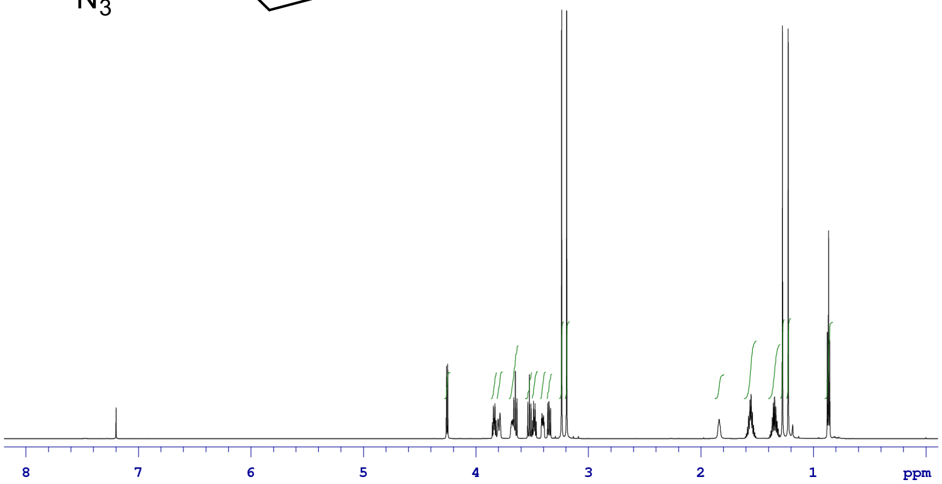

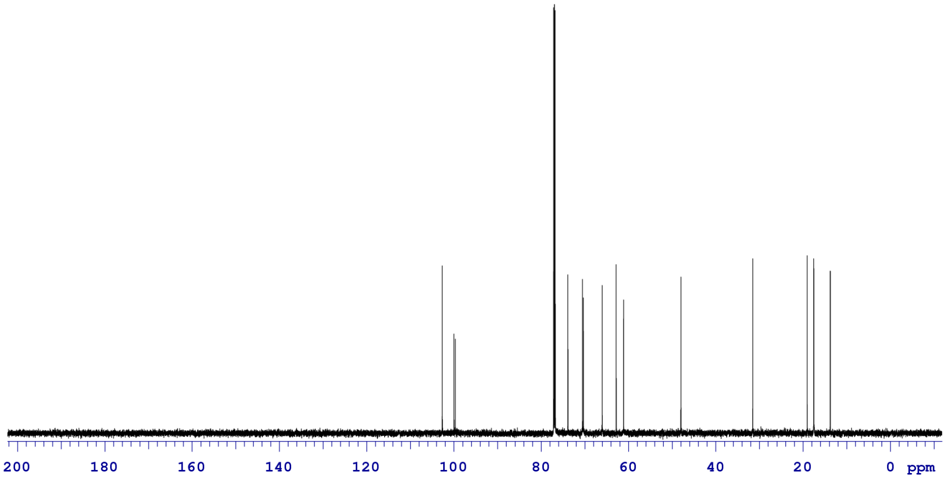

Sugar Silane 10 and Butanol Silyl-Linked (11i)

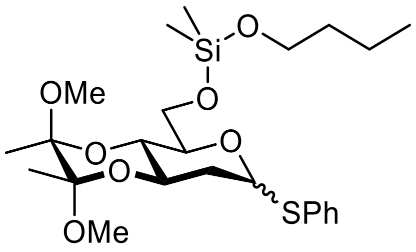

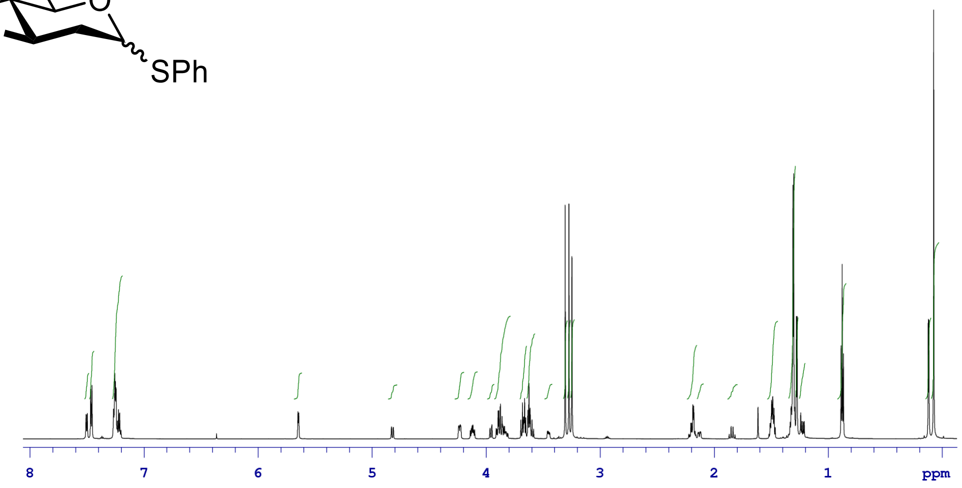

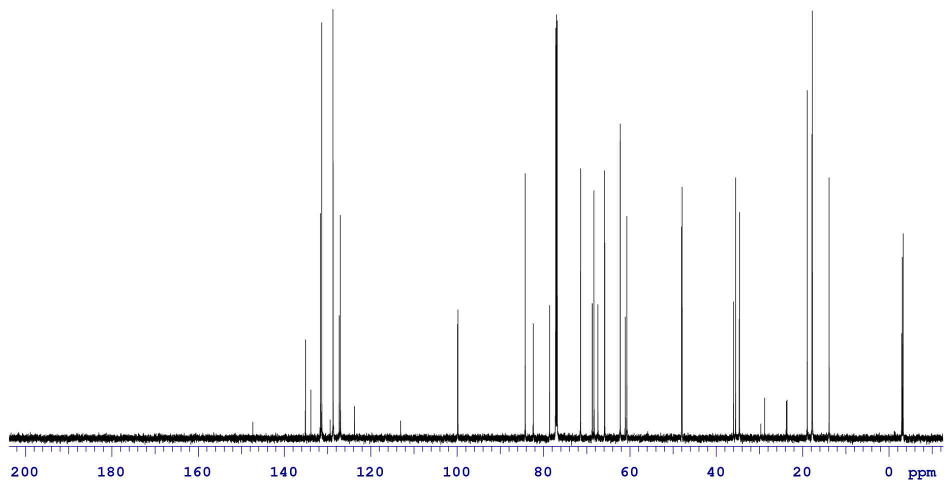

Sugar Silane 10 and Butanol Glycoside (12i)

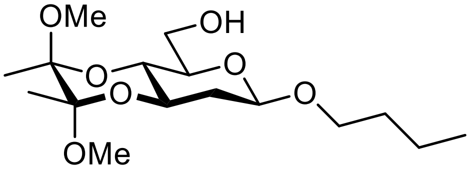

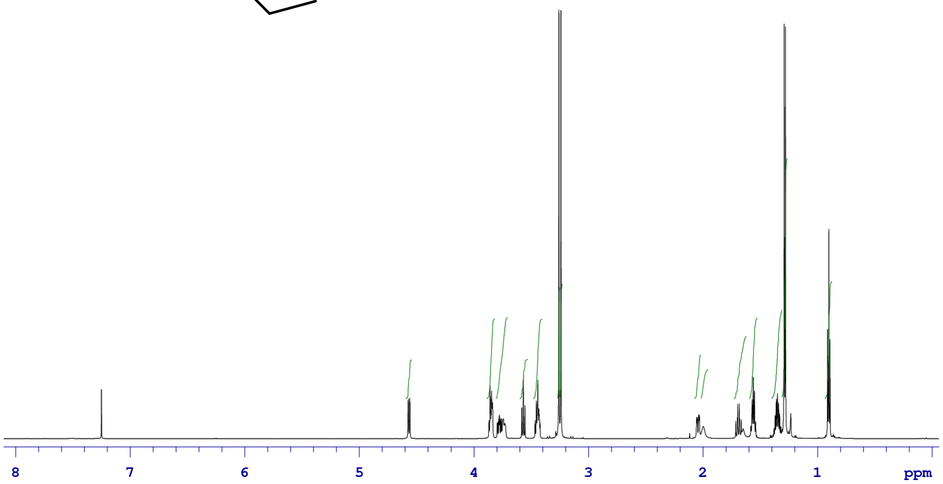

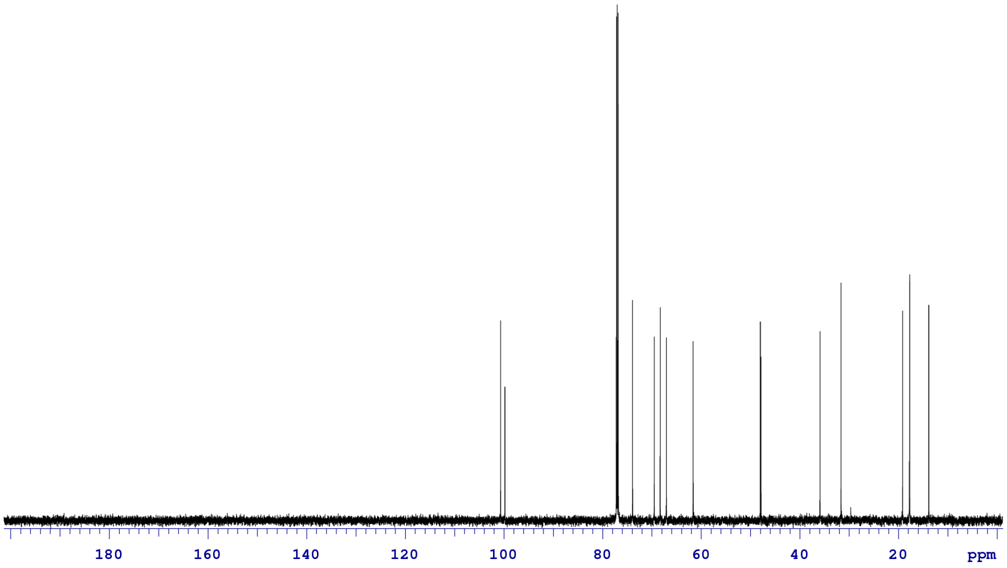

1,6-Anhydro Glucopyranoside (18)

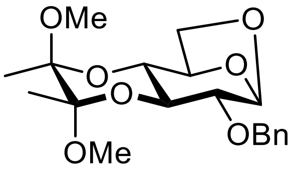

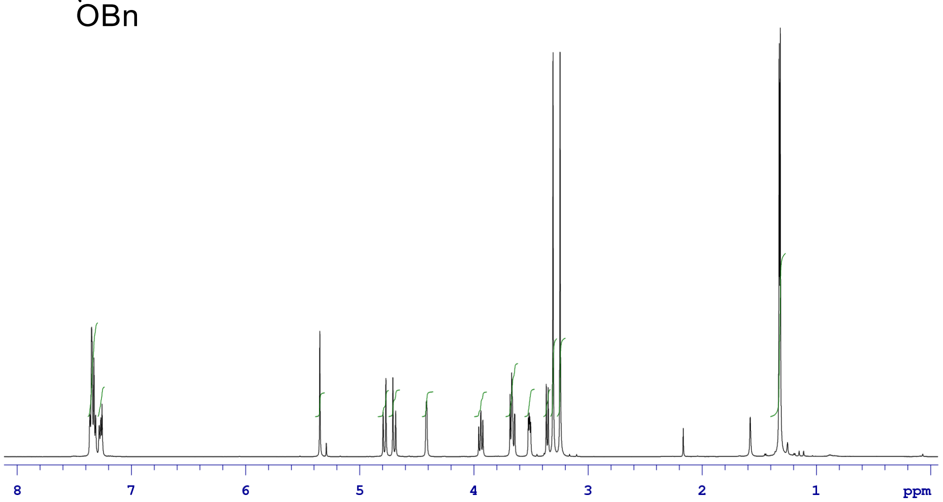

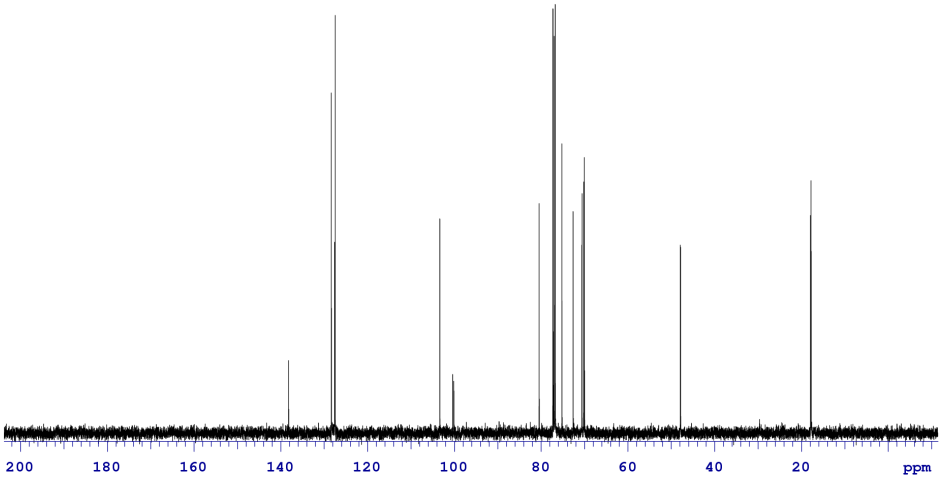

7a Silyl-Linked Dimer (19)

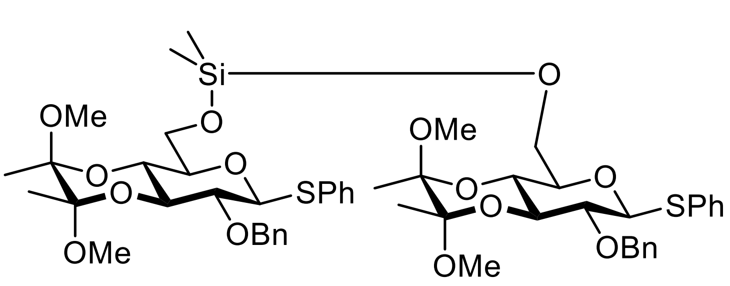

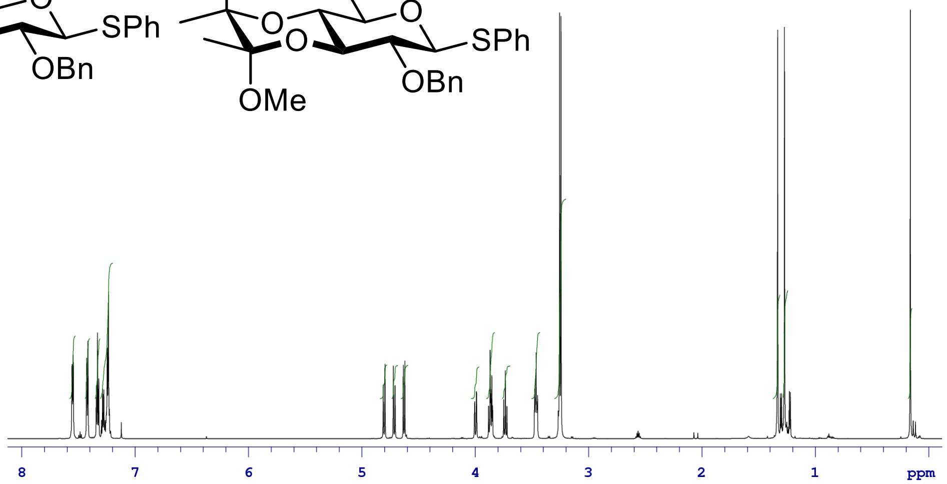

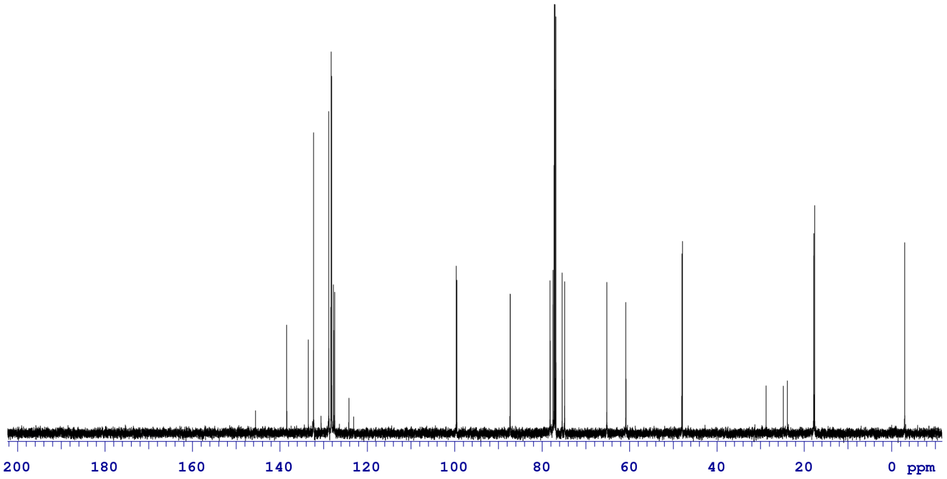

Supplement: Supplementary file 1 [file SC-006-C5SC00810G-s001.pdf]
